# Perovskite BaTaO_2_N: From Materials Synthesis to Solar Water Splitting

**DOI:** 10.1002/advs.202305179

**Published:** 2023-10-18

**Authors:** Mirabbos Hojamberdiev, Ronald Vargas, Fuxiang Zhang, Katsuya Teshima, Martin Lerch

**Affiliations:** ^1^ Institut für Chemie Technische Universität Berlin Straße des 17. Juni 135 10623 Berlin Germany; ^2^ Instituto Tecnológico de Chascomús (INTECH) – Consejo Nacional de Investigaciones Científicas y Técnicas (CONICET) Universidad Nacional de San Martín (UNSAM) Avenida Intendente Marino, Km 8,2, (B7130IWA) Chascomús Provincia de Buenos Aires Argentina; ^3^ Escuela de Bio y Nanotecnologías Universidad Nacional de San Martín (UNSAM) Avenida Intendente Marino, Km 8,2, (B7130IWA) Chascomús Provincia de Buenos Aires Argentina; ^4^ State Key Laboratory of Catalysis iChEM Dalian Institute of Chemical Physics Chinese Academy of Sciences Dalian National Laboratory for Clean Energy Dalian 116023 P.R. China; ^5^ Department of Materials Chemistry Shinshu University 4‐17‐1 Wakasato Nagano 3808553 Japan; ^6^ Research Initiative for Supra‐Materials Shinshu University 4‐17‐1 Wakasato Nagano 3808553 Japan

**Keywords:** BaTaO_2_N, crystal structure, oxynitride, photocatalysis, synthesis, water splitting

## Abstract

Barium tantalum oxynitride (BaTaO_2_N), as a member of an emerging class of perovskite oxynitrides, is regarded as a promising inorganic material for solar water splitting because of its small band gap, visible light absorption, and suitable band edge potentials for overall water splitting in the absence of an external bias. However, BaTaO_2_N still exhibits poor water‐splitting performance that is susceptible to its synthetic history, surface states, recombination process, and instability. This review provides a comprehensive summary of previous progress, current advances, existing challenges, and future perspectives of BaTaO_2_N for solar water splitting. A particular emphasis is given to highlighting the principles of photoelectrochemical (PEC) water splitting, classic and emerging photocatalysts for oxygen evolution reactions, and the crystal and electronic structures, dielectric, ferroelectric, and piezoelectric properties, synthesis routes, and thin‐film fabrication of BaTaO_2_N. Various strategies to achieve enhanced water‐splitting performance of BaTaO_2_N, such as reducing the surface and bulk defect density, engineering the crystal facets, tailoring the particle morphology, size, and porosity, cation doping, creating the solid solutions, forming the heterostructures and heterojunctions, designing the photoelectrochemical cells, and loading suitable cocatalysts are discussed. Also, the avenues for further investigation and the prospects of using BaTaO_2_N in solar water splitting are presented.

## Graphical abstract

This review provides a comprehensive summary of previous progress, current advances, existing challenges, and future perspectives of BaTaO_2_N for solar water splitting. For enhancing the water‐splitting performance of BaTaO_2_N, various strategies, such as reducing the defect density, engineering the crystal facets, cation doping, forming the heterostructures/heterojunctions, designing the photoelectrochemical cells, and loading suitable cocatalysts are presented.

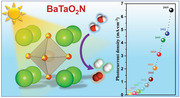

## 1 Photoelectrochemical (PEC) Water Splitting

**Figure 1:**
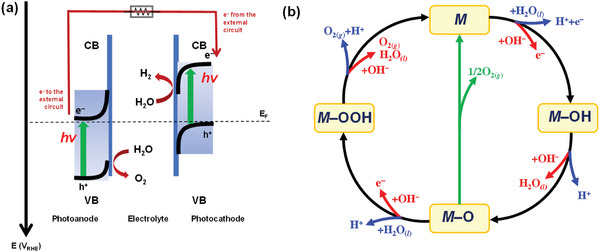
a) Working principle of the photoelectrochemical cell for water splitting using a photoanode and a photocathode. b) OER mechanism for acid (blue line) and alkaline (red line) conditions. The black and green lines indicate that the oxygen evolution involves the formation of a peroxide (M–OOH) intermediate and the direct reaction of two adjacent oxo (M–O) intermediates, respectively. Reproduced with permission.^[^ ^16^ ^]^ Copyright 2017, The Royal Society of Chemistry.

To tackle the rising global energy demand and to reduce greenhouse gas emissions from the combustion of diminishing fossil fuels, the development of renewable energy is indispensable. Hydrogen is regarded as a potential zero‐emission energy carrier with the highest gravimetric energy density (120 MJ kg^−1^) despite its low volumetric energy density (8 MJ L^−1^)^[^ ^1^ ^]^ and it can play a vital role in the successful implementation of the Paris Agreement,^[^ ^2^ ^]^ which is essential for the achievement of the 17 United Nations Sustainable Development Goals. However, the current global hydrogen production still heavily relies on fossil fuels (>95%), which is the cheapest option in most parts of the world.^[^ ^3^, ^4^ ^]^ Solar‐driven water splitting is one of the most environmentally benign chemical processes to convert abundant solar energy into storable and transportable green hydrogen.^[^ ^5^, ^6^, ^7^, ^8^ ^]^ Solar‐driven water splitting proceeds over cocatalyst‐assisted semiconductor according to three consecutive steps (**Figure** **1**a): i) the absorption of photons with higher energy values to excite electrons from the valence band to the conduction band, ii) the separation and transfer of photo‐excited charge carriers (electrons and holes) from the bulk to the surface of the semiconductor, and iii) the initiation of water redox reactions by the involvement of photo‐excited charge carriers^[^ ^9^ ^]^:

Hydrogen evolution reaction (HER):

(1) $$2H^{+}+2e^{-}\to H_{2}\left(\mathrm{acidic}\;\mathrm{media}\right)$$

(2) $$2H_{2}O+2e^{-}\to H_{2}+2OH^{-}\left(\mathrm{alkaline}\;\mathrm{media}\right)$$

Oxygen evolution reaction (OER):

(3) $$2H_{2}O\to O_{2}+4e^{-}+4H^{+}\left(\mathrm{acidic}\right)$$

(4) $$4OH^{-}\to O_{2}+4e^{-}+2H_{2}O\left(\mathrm{alkaline}\right)$$

Particularly, photoelectrochemical (PEC) water splitting is a powerful yet complex process, where the redox potentials for the decomposition of water determine the required band gap energy and band‐edge potentials for semiconductors to be used as photoanodes and photocathodes. However, to efficiently and sustainably split water into H_2_ and O_2_, several key criteria must be met simultaneously: i) sufficient voltage must be generated upon irradiation to split water, ii) the bulk band gap must be small enough to absorb a significant portion of the solar spectrum, iii) for the unbiased operation of a PEC cell, the band edge potentials at the surfaces must straddle the hydrogen and oxygen redox potentials (the conduction band minimum must be more negative than *E* ^0^(H^+^/H_2_) = 0 V versus RHE at pH 0 and the valence band maximum must be more positive than *E* ^0^(O_2_/H_2_O) = 1.23 V versus RHE at pH 0), iv) the system must be stable for a long period of reaction time, v) charge transfer from the semiconductor surface to the electrolyte must be facile to minimize energy losses, and vi) low‐cost and earth‐abundant elements must be used.^[^ ^10^ ^]^

In addition to these important requirements, the photoelectrode materials must exhibit at least >10% solar‐to‐hydrogen (STH) conversion efficiency for their commercial viability.^[^ ^11^ ^]^ The U.S. Department of Energy estimated the cost of green hydrogen produced by the PEC process to be US$5.7 kg^−1^ in 2020 (with a 20% STH efficiency) and to lower to US$2.1 kg^−1^ (with a 25% STH efficiency) in the more distant future.^[^ ^12^ ^]^ It has also been suggested that the STH efficiency of 25% and the photoelectrode lifetime of 10 years are required for the PEC systems to be economically consistent with fossil fuel‐based hydrogen production processes.^[^ ^13^ ^]^ Recently, monolithic systems and integrated PEC modules under light concentration reached STH efficiencies as high as 17.12%^[^ ^14^ ^]^ and 19%.^[^ ^15^ ^]^ However, the high capital and operational costs significantly hamper their economic viability. To date, no cost‐effective and highly efficient PEC systems have been developed to satisfy all those key criteria despite significant progress in this field. Thus, further studies are necessary to discover novel materials with unprecedented physicochemical and optoelectronic properties that can simultaneously meet those key criteria set for the design and application of the PEC system for green hydrogen production.

As one of the two key reactions of overall water splitting, the water oxidation reaction proceeds via the following general steps (Figure 1b)^[^ ^16^ ^]^: i) water dissociation and formation of surface‐bonded OH_ads_, ii) the further oxidation of OH_ads_ to O_ads_, and iii) the formation of OOH_ads_, which is a precursor for O_2_ evolution^[^ ^17^, ^18^ ^]^:

(5) $$H_{2}O⇄{\mathrm{OH}}_{\mathrm{ads}}+H^{+}+e^{-}$$

(6) $${\mathrm{OH}}_{\mathrm{ads}}⇄O_{\mathrm{ads}}+H^{+}+e^{-}$$

(7) $$H_{2}O+O_{\mathrm{ads}}⇄{\mathrm{OOH}}_{\mathrm{ads}}+H^{+}+e^{-}$$

(8) $${\mathrm{OOH}}_{\mathrm{ads}}⇄O_{2}+H^{+}+e^{-}$$

Using first‐principles periodic density functional theory (DFT) calculations, Man et al.^[^ ^19^ ^]^ proposed that the binding energies of reaction intermediates (e.g., HO*, O*, and HOO*) are responsible for the origin of OER activity over select oxide surfaces. When the binding energy of oxygen is quite high, the OER overpotential is limited by the formation of HOO* species, and otherwise, the formation of HO* species is dominant. Therefore, various strategies to stabilize HOO* species compared with HO* species were developed.^[^ ^20^ ^]^ The suitable photoanodes must satisfy the expected conditions: Δ*G*(OH_ads_) = C_O_ = 1.23 eV, Δ*G*(O_ads_) = 2 × C_O_ = 2.46 eV, and Δ*G*(OOH_ads_) = 3 × Co = 3.69 eV.^[^ ^18^ ^]^ The water oxidation reaction requires the transfer of four electrons and four protons, which needs high overpotential, with the simultaneous formation of the O–O bond in comparison to the two‐electron‐transfer water reduction reaction.^[^ ^21^ ^]^ Therefore, water oxidation is thermodynamically and kinetically more challenging than water reduction. As shown in Figure 1b, a sufficient amount of energy must be supplied in each step to drive the water oxidation reaction. Thus, it leads to high energy barriers and slow kinetics in addition to the uphill reaction thermodynamics.^[^ ^22^ ^]^ A sluggish water oxidation reaction is the rate‐determining step that governs the reaction rate of water splitting.^[^ ^23^ ^]^ The water oxidation reaction is generally enhanced by modifying the photoanodes with oxygen evolution cocatalysts that promote the efficient charge separation of photo‐excited electron‐hole pairs.^[^ ^24^ ^]^

According to the three basic stages of water splitting, the efficiency of the water oxidation reaction is mainly determined by the light absorption, the separation efficiency of photo‐excited charge carriers, and the surface catalytic reaction^[^ ^9^ ^]^:

(9) $$\eta =\eta_{\mathrm{absorption}}\times \eta_{\mathrm{separation}}\times \eta_{\mathrm{reaction}}$$

which can be controlled by modulating the physicochemical, optoelectronic, and surface properties of *n*‐type semiconductors. Also, the valence band edge potentials of photocatalysts must be more positively positioned than that of *E* ^0^(O_2_/H_2_O) and the water oxidation active sites must be sufficient on the photocatalyst surface to hinder the recombination of photo‐excited electrons and holes.^[^ ^25^ ^]^

## 2 Oxide‐Based Photocatalysts for PEC OER

**Figure 2:**
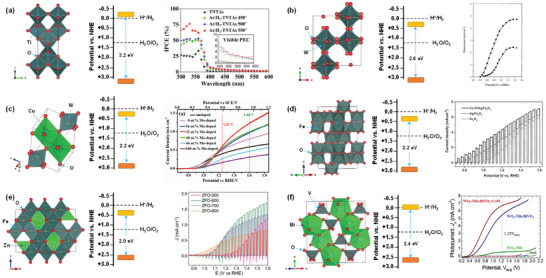
a) Crystal and band structures of TiO_2_ and IPCE spectra of reduced TiO_x_ nanotube arrays before and after Ar/H_2_ treatment at different temperatures. Reproduced with permission.^[^ ^30^ ^]^ Copyright 2017, WILEY‐VCH Verlag GmbH & Co. KGaA. b) Crystal and band structures of WO_3_ and photocurrent‐potential plots for a 2‐µm‐thick WO_3_ electrode illuminated with AM 1.5G simulated sunlight: in 1 M aq. HClO_4_ (curve A) and after the addition of 0.1 mol dm 0^−3^ of methanol (curve B). Reproduced with permission.^[^ ^36^ ^]^. Copyright 2001, American Chemical Society. c) Crystal and band structures of CuWO_4_ and linear potential sweep curves of the doped CuWO_4_ nanoflake photoanodes with different Mo doping concentrations. Reproduced with permission.^[^ ^39^ ^]^ Copyright 2019, Elsevier. d) Crystal and band structures of Fe_2_O_3_ and photoelectrochemical performance of Fe_2_O_3_, Ag/Fe_2_O_3_, and Co‐Pi/Ag/Fe_2_O_3_ photoelectrodes under chopped light illumination in 1 M NaOH (pH 13.6). Reproduced with permission.^[^ ^47^ ^]^ Copyright 2016, WILEY‐VCH Verlag GmbH & Co. KGaA. e) Crystal and band structures of ZnFe_2_O_4_ and linear scanning J–V curves of ZnFe_2_O_4_ photoanodes in 1 M NaOH under intermittent 1 sun illumination (100 mW cm^−2^). Reproduced with permission.^[^ ^51^ ^]^ Copyright 2018, WILEY‐VCH Verlag GmbH & Co. KGaA. f) Crystal and band structures of BiVO_4_ and I‐V characteristics of the optimized WO_3_‐nanorods (green), WO_3_‐nanorods/BiVO_4_ (blue) and WO_3_‐nanorods/BiVO_4_+CoPi (red) samples. Reproduced with permission.^[^ ^56^ ^]^ Copyright 2015, Springer Nature AG & Co. KGaA.

Since the first successful demonstration of solar‐induced unassisted water splitting over a TiO_2_ photoanode by Honda and Fujishima,^[^ ^26^ ^]^ a large number of studies have been conducted to maximize the STH efficiency of various heterogeneous oxide‐based semiconductors but few have shown relatively outstanding water oxidation performance and stability. The overview of some *n*‐type oxide semiconductors for PEC OER is shown in **Figure** **2** and **Table** **1**.^[^ ^27^ ^]^

**Table 1:** Overview of some *n*‐type oxide semiconductors for PEC OER.

Compound	Structure type and symmetry	Band gap	Theoretical STH efficiency and photocurrent density	Performance/efficiency	Limitations	Reference
TiO_2_	Tetragonal anatase, *I*4_1_/*amd*	3.2 eV	1.3%, 1.1 mA cm^−2^	75% IPCE	fast recombination rate, rapid backward reaction, and a large overpotential for HER, and bandgap limits the STH efficiency to ≈1%	[29, 30, 31]
WO_3_	Monoclinic, *P*2_1_/*n*	2.6 eV	4.8%, 3.9 mA cm^−2^	75% IPCE, 2.4 mA cm^−2^ at 1.23 V vs RHE	rapid recombination rate, slow charge transfer at semiconductor/electrolyte, a restricted light absorption up to 450 nm, instability at pH>5, and limited theoretical STH efficiency	[31, 36]
CuWO_4_	Triclinic distorted wolframite, *P* $\bar{1}$	2.2 eV	≈13%, 10.7 mA cm^−2^	>20% IPCE, ≈0.15‐0.16 mA cm^−2^, and 0.62 mA cm^−2^ at 1.23 V vs RHE	low light absorption coefficient and high bulk charge transfer resistance	[37, 38, 39, 40, 41, 42]
α‐Fe_2_O_3_	Trigonal corundum, *R* $\bar{3}c$	2.0‐2.2 eV	16.8%, 12.6 mA cm^−2^	≈18% IPCE, ≈6 mA cm^−2^ at 1.23 V vs RHE, ≈40% IPCE, and ≈0.55% STH	a low absorption coefficient, short excited‐state lifetime, short hole‐diffusion length, low charge carrier mobility, poor electric conductivity, and poor OER kinetics	[44, 45, 46, 47]
ZnFe_2_O_4_	Cubic spinel, *Fd* $\bar{3}m$	2.0 eV	20%, ≈11 mA cm^−2^	25% IPCE 1.0 mA cm^−2^ at 1.23 V vs RHE	rapid surface and bulk recombination rate and poor minority career	[49, 51]
BiVO_4_	Monoclinic scheelite, *C*2/*c*	2.4 eV	9.1%, 7.4 mA cm^−2^	6.72 mA cm^−2^ at 1.23 V vs RHE, ≈90% IPCE, 8.1% STH, and 1.75% ABPE at 0.6 V vs RHE	high recombination rate, poor charge transport properties, low carrier collection efficiency, inadequate water oxidation kinetics, and limited theoretical STH efficiency	[31, 56, 58]

TiO_2_ is one of the most widely studied oxide semiconductors because of its low cost, chemical stability, earth abundance, non‐corrosiveness, and non‐toxicity. TiO_2_ typically exists in three crystalline structures: anatase (tetragonal, *I*4_1_/*amd*), rutile (tetragonal, *P*4_2_/*mnm*), and brookite (orthorhombic, *Pbca*). In addition to other factors, the STH efficiency of TiO_2_ depends on its polymorphs. The photocatalytic overall water splitting reaction can take place on rutile but hardly on anatase and brookite and becomes feasible for anatase and brookite only under prolonged UV light irradiation.^[^ ^28^ ^]^ Recently, compact anatase TiO_2_ layers fabricated on the FTO by introducing a Ti interlayer and suboxide TiO_2_ nanotubes exhibited the highest incident photon‐to‐current efficiency (IPCE) value of 75% at 300 nm^[^ ^29^ ^]^ and 340 nm (Figure 2a),^[^ ^30^ ^]^ respectively. Although the anatase‐TiO_2_ polymorph has better electron mobility and conductivity in comparison to the rutile‐TiO_2_ and brookite‐TiO_2_ polymorphs, its theoretical STH efficiency and photocurrent density can only reach the maximum of 1.3% and 1.1 mA cm^−2^, respectively, because of wide optical bandgap energy of 3.2 eV.^[^ ^31^ ^]^ In addition to the low STH efficiency, a fast recombination rate of photo‐excited charge carriers, a rapid backward reaction (recombination of H_2_ and O_2_), and a large overpotential for the HER also hamper the practical application of TiO_2_ in solar water splitting despite considerable progress.^[^ ^32^, ^33^ ^]^

The monoclinic phase with space group *P*2_1_/*n* is the most stable polymorph of WO_3_ at room temperature and has a perovskite‐like structure without an *A*‐site ion. Theoretical studies on the correlation between the crystal structure and the band gap of WO_3_ revealed that the bandgap energy values of different WO_3_ polymorphs decrease in the following order: monoclinic > orthorhombic > triclinic > tetragonal ≥ cubic.^[^ ^34^ ^]^ Also, the structural modification by introducing the nitride ions into the lattice of the monoclinic WO_3_ phase led to the reduction of the bandgap energy from 2.6 eV to 1.9 eV.^[^ ^35^ ^]^ Highly transparent nanoporous WO_3_ films fabricated by layer‐by‐layer deposition of a colloidal solution of tungstic acid and annealing exhibited a maximum IPCE of 75% and a photocurrent density of 2.4 mA cm^−2^ at 1.23 V versus RHE under simulated solar irradiation (Figure 2b).^[^ ^36^ ^]^ However, the rapid recombination of photo‐excited charge carriers, slow charge transfer at the WO_3_/electrolyte, restricted light absorption up to 450 nm, instability at pH > 5, and a theoretical STH efficiency of 4.8% and a photocurrent density of 3.9 mA cm^−2^ still limit its application.^[^ ^31^ ^]^

To circumvent the drawbacks of WO_3_, triclinic CuWO_4_ with a distorted wolframite crystal structure (space group *P* $\bar{1}$) offers a smaller bandgap energy and limits the formation of soluble tungstates due to the strong covalency in the metal oxo bonds, yielding higher photocurrent in neutral pH under visible light irradiation.^[^ ^37^ ^]^ The CuWO_4_ photoanodes exhibited photocurrent densities of ≈0.15–0.16 mA cm^−2^ and 0.62 mA cm^−2^ at the thermodynamic potential for water oxidation (1.23 V versus RHE) under simulated solar irradiation (Figure 2c)^[^ ^37^, ^38^, ^39^ ^]^ and an IPCE efficiency of >20%.^[^ ^40^ ^]^ Despite its theoretical STH efficiency of ≈13% and photocurrent density of 10.7 mA cm^−2^, the PEC performance of CuWO_4_ is significantly hindered by its low light absorption coefficient and high bulk charge transfer resistance.^[^ ^41^, ^42^ ^]^

α‐Fe_2_O_3_ with a trigonal structure (space group *R* $\bar{3}c$) has been intensively explored as a promising photoanode material for PEC water splitting due to its small bandgap energy, suitable valence band edge potential to thermodynamically drive the water oxidation reaction, excellent stability under alkaline conditions, earth abundance, and environmentally friendliness.^[^ ^43^, ^44^ ^]^ With the reported bandgap energy of 2.0‐2.2 eV, α‐Fe_2_O_3_ has the potential to reach a theoretical maximum STH efficiency of 16.8% and a photocurrent density of 12.6 mA cm^−2^,^[^ ^45^ ^]^ which manifestly exceeds the STH benchmark efficiency of 10% required for practical applications. The α‐Fe_2_O_3_ nanowires‐based photoelectrode, which was fabricated by a chemical bath deposition method followed by hydrogen treatment and then loaded with ultrathin TiO_2_ overlayer and CoPi cocatalyst, exhibited a stable photocurrent density of ≈ 6 mA cm^−2^ at 1.23 V versus RHE over 100 h under AM 1.5G simulated sunlight and an IPCE value of ≈18% at 420 nm owing to its excellent light absorption and bulk charge separation capacity, the enhanced electrical conductivity, and the decreased surface recombination.^[^ ^46^ ^]^ A hematite nanosheets‐based electrode modified by Ag and CoPi nanoparticles showed an IPCE value of ≈40% at 420 nm and an STH efficiency of ≈0.55% due to the improved light harvesting and the facilitated charge transfer by Ag nanoparticles and the reduction of surface/bulk recombination and the stabilization of the photoelectrode surface by CoPi cocatalyst (Figure 2d).^[^ ^47^ ^]^ However, several limitations, such as low absorption coefficient due to an indirect band gap, short excited‐state lifetime (≈10^−12^ s), short hole‐diffusion length (2‐4 nm), low charge carrier mobility (≈10^–2^ to ≈10^–1^ cm^2^ V^−1^ s^−1^), poor electric conductivity, and poor OER kinetics still hinder achieving the practical maximum STH efficiency of α‐Fe_2_O_3_.^[^ ^44^, ^48^ ^]^

Cubic spinel ZnFe_2_O_4_ (space group *Fd* $\bar{3}m$) also received much interest due to its narrow bandgap energy (*E*_g_ = 1.9 eV), high energy level conduction band minimum, outstanding photochemical stability, low cost, and magnetic recyclability.^[^ ^49^, ^50^ ^]^ Interestingly, ZnFe_2_O_4_ with a relatively poor crystallinity but a higher spinel inversion degree (due to cation disorder) shows a superior efficiency in photo‐excited charge carrier separation and an improved majority charge carrier transport compared to ZnFe_2_O_4_ with higher crystallinity but a lower inversion degree.^[^ ^51^ ^]^ The optimization of these factors and the addition of a nickel‐iron cocatalyst overlayer resulted in a new benchmark photocurrent density of 1.0 mA cm^−2^ at 1.23 V versus RHE, and an IPCE value of about 25% was reached for ZnFe_2_O_4_ nanorod photoanode (Figure 2e).^[^ ^51^ ^]^ At potentials between 0.8 and 1.3 V versus RHE, ZnFe_2_O_4_ can exhibit a considerably higher charge transfer efficiency due to a slower surface charge recombination rate.^[^ ^52^ ^]^ Despite its maximum theoretical STH efficiency of about 20% and a photocurrent density of ≈11 mA cm^−2^,^[^ ^49^, ^51^ ^]^ the efficiency of ZnFe_2_O_4_ is substantially limited by a rapid surface and bulk recombination rate of photo‐excited charge carriers and poor minority career.

BiVO_4_ crystallizes in three different polymorphs: monoclinic scheelite, tetragonal scheelite, and tetragonal zircon. The monoclinic scheelite BiVO_4_ (space group *C*2/*c*) is an *n*‐type semiconductor with a direct bandgap energy of 2.4 eV and a valence band edge potential of ≈2.4 V versus RHE, which is sufficiently positive than *E* ^0^(O_2_/H_2_O) = 1.23 V versus RHE.^[^ ^53^, ^54^ ^]^ Therefore, the monoclinic scheelite BiVO_4_ shows the highest photocatalytic water oxidation activity among the three polymorphs.^[^ ^55^ ^]^ A photocurrent density of 6.72 mA cm^−2^ at 1.23 V versus RHE and an IPCE of ≈90% were achieved by the combination of BiVO_4_ with more conductive WO_3_ nanorods in the form of core‐shell heterojunction (Figure 2f).^[^ ^56^ ^]^ The high recombination rate of photo‐excited charge carriers was significantly reduced by creating a thin absorber layer of BiVO_4_, which was thinner than the carrier diffusion length. The tandem device constructed with a GaAs/InGaAsP solar cell exhibited an STH efficiency of 8.1%.^[^ ^56^ ^]^ The charge separation efficiency of 97.1% and charge transfer efficiency of 90.1% at 1.23 V versus RHE were obtained by engineering the hierarchical nanoporosity of BiVO_4_,^[^ ^57^ ^]^ and an applied bias photon‐to‐current conversion efficiency (ABPE) of 1.75% was found at a potential as low as 0.6 V versus RHE for nanoporous BiVO_4_ photoanodes.^[^ ^58^ ^]^ However, the water oxidation efficiency of BiVO_4_ is still limited by a high electron‐hole recombination rate, poor charge transport properties, a low carrier collection efficiency, inadequate water oxidation kinetics, and the bandgap energy limiting an AM 1.5G solar photocurrent density to 7.4 mA cm^−2^ and an STH efficiency to 9.1%.^[^ ^31^ ^]^

Despite their encouraging and relatively outstanding performance achieved so far, TiO_2_, WO_3_, CuWO_4_, Fe_2_O_3_, ZnFe_2_O_4_, and BiVO_4_ alone are unlikely to satisfy the criteria set for ensuring the practical application in solar to chemical energy conversion. This paved the way for the development of novel materials, including mixed‐anion compounds, for PEC applications.

## 3 Crystal and Electronic Structures of Perovskite BaTaO_2_N

**Figure 3:**
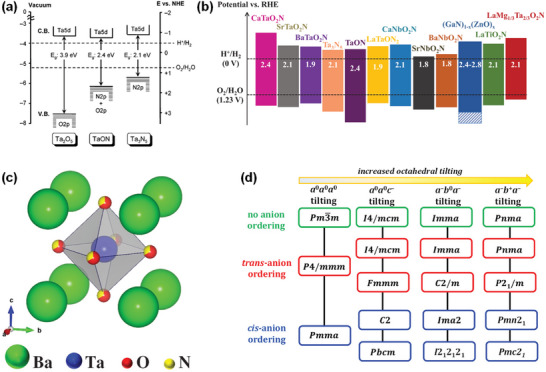
a) Schematic representation of the band structures of Ta_2_O_5_, TaON, and Ta_3_N_5_. Reproduced with permission.^[^ ^62^ ^]^ Copyright 2007, American Chemical Society. b) Bandgaps and energy diagrams of several types of typical (oxy)nitrides. Reproduced with permission.^[^ ^64^ ^]^ Copyright 2021, WILEY‐VCH Verlag GmbH & Co. KGaA. c) Crystal structure of BaTaO_2_N.^[^ ^70^ ^]^ d) Space groups stemming from *cis*‐ and *trans*‐anion ordering and octahedral tilting in mixed‐anion perovskites. Reproduced with permission.^[^ ^81^ ^]^ Copyright 2014, American Chemical Society.

Mixed‐anion compounds, containing more than one anionic species in a single phase, are an emerging class of advanced materials with the potential to contribute to solar fuel production in the future.^[^ ^59^ ^]^ Unlike single‐anion compounds, mixed‐anion compounds exhibit diverse structures, chemical and physical properties, and new functionalities because of different anionic characteristics, such as ionic radii, valency, electronegativity, and polarizability.^[^ ^60^ ^]^ Also, the combination of hetero‐anions can realize the structures of compounds that cannot be generally stabilized by homo‐anions. Especially, to develop photocatalytic materials that can efficiently function under visible light, anions less electronegative than oxygen can be simultaneously introduced. Having similar chemical, structural, and electronic properties, oxygen and nitrogen substitute each other in the anion site to form oxynitrides.^[^ ^61^ ^]^ In oxynitrides, the nitride anions (N^3−^) having atomic orbitals with potential energy higher than O 2p atomic orbitals of the oxide anions (O^2−^) shift the valence band maximum upward without affecting the conduction band minimum, leading to the increased covalency of metal‐anion bonds, the improved absorption property, and the decreased optical band gap (**Figure** **3**a).^[^ ^62^, ^63^ ^]^ Also, most *d* ^0^ transition metal oxynitrides can absorb photons with absorption band edges in the range of 500–760 nm and have theoretical STH conversion efficiencies in the range of 8–32% and suitable conduction and valence band edge potentials straddling the proton reduction and water oxidation reaction potentials, respectively, and can drive the overall water splitting reaction (Figure 3b).^[^ ^64^ ^]^

As a typical representative of the *AB*(O,N)_3_ perovskites, BaTaO_2_N is regarded as one of the promising photocatalysts for solar water splitting due to its absorption of visible light up to 660 nm, small bandgap energy (*E*_g_ = 1.9 eV), good stability under light irradiation in concentrated alkaline solutions, and nontoxicity.^[^ ^65^ ^]^ Moreover, the conduction band minimum and valence band maximum of BaTaO_2_N are located at –0.4 V and 1.5 V versus NHE at pH 0, respectively, which should theoretically drive the water‐splitting reaction in the absence of an external bias.^[^ ^66^ ^]^ BaTaO_2_N can generate a photocurrent density of about 18 mA·cm^−2^ under AM 1.5G simulated sunlight based on the assumption of an IPCE efficiency of 100% at < 660 nm.^[^ ^67^ ^]^ Incidentally, the STH conversion efficiency of BaTaO_2_N is about 24%.^[^ ^10^ ^]^ Its large dielectric constant can promote a facile separation of photo‐excited charge carriers.^[^ ^68^ ^]^

**Figure 4:**
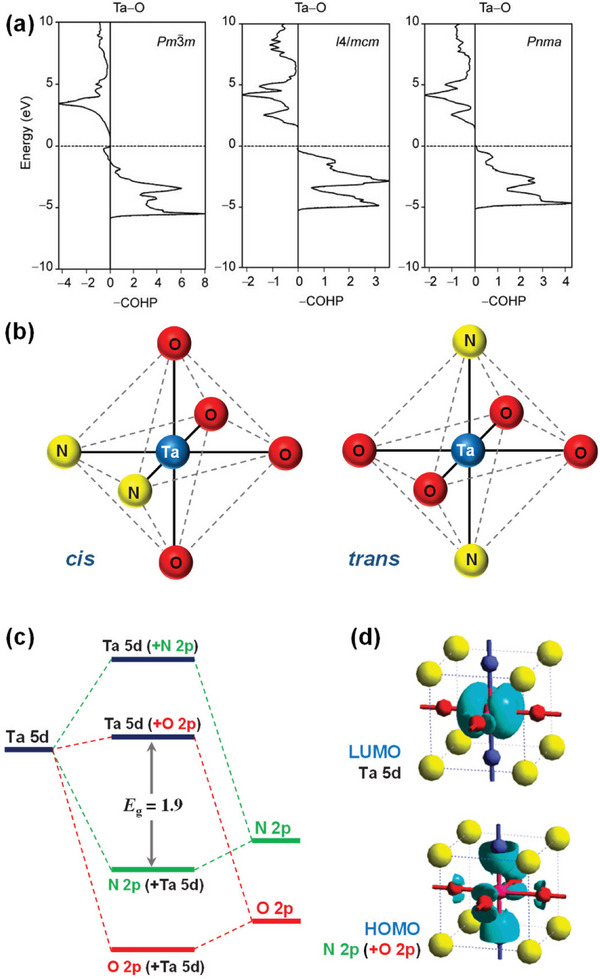
a) Crystal Orbital Hamilton Populations (COHP) for the Ta–O interactions in three different polymorphs of BaTaO_2_N. Reproduced with permission.^[^ ^78^ ^]^ Copyright 2008, WILEY‐VCH Verlag GmbH & Co. KGaA. b) Local *cis* and *trans* ordering of TaO_4_N_2_ octahedron in BaTaO_2_N. c) Schematic representation of the band structure of BaTaO_2_N and d) DFT‐calculated band structure of BaTaO_2_N. Reproduced with permission.^[^ ^62^ ^]^ Copyright 2007, American Chemical Society.

The BaTaO_2_N phase was first synthesized in 1986 by Marchand et al.,^[^ ^69^ ^]^ and its centrosymmetric cubic crystal structure with space group $Pm\bar{3}m$ (no. 221) and a random O/N distribution were determined by a powder time‐of‐flight neutron diffraction method (Figure 3c).^[^ ^70^ ^]^ Ba is at the origin (0,0,0), Ta is at the cube center (1/2, 1/2, 1/2), and three anions (one nitrogen and two oxygen) are randomly distributed at the face centers. The Ba atom is 12‐fold surrounded by anions with a Ba–(O,N) distance of 2.908 Å, and the Ta atom is octahedrally coordinated (Ta(O,N_6_) with a Ta–(O,N) distance of 2.056 Å.^[^ ^69^, ^70^ ^]^ Although the conventional diffraction analyses using neutron^[^ ^70^ ^]^ and X‐ray^[^ ^68^ ^]^ radiations similarly confirmed an average cubic perovskite structure with $Pm\bar{3}m$ symmetry, the extended X‐ray absorption fine structure (EXAFS),^[^ ^71^ ^]^ zone‐axis electron diffraction (ED),^[^ ^72^ ^]^ pair‐distribution‐function (PDF),^[^ ^73^ ^]^ solid‐state magic‐angle spinning (MAS) NMR spectroscopy,^[^ ^74^ ^]^ constant‐wavelength neutron diffraction,^[^ ^75^ ^]^ and X‐ray absorption near edge structure (XANES) spectroscopy^[^ ^76^ ^]^ analyses revealed short‐range structural distortions around the Ta atoms and widely distributed Ta–O/N bond distances in BaTaO_2_N. First‐principles investigation conducted by Xu and Jiang^[^ ^77^ ^]^ found the existence of both short‐range order in the intra‐octahedron O/N occupation and randomness of inter‐octahedron O/N occupation, which is consistent with the cubic symmetry. Their study also revealed that the electronic properties of BaTaO_2_N do not strongly depend on the O/N configuration, whereas the dielectric properties indicate a stronger dependence on the O/N configuration. Since local structure relaxation is expected due to the different ionic radii and valency of the oxygen and nitrogen ions, the local symmetry of BaTaO_2_N was, however, not well defined. The DFT analyses suggested that the local symmetry in the crystal is lower, resulting in the different Ta–O and Ta–N bond lengths, and it predicted the possibility of either orthorhombic, tetragonal, or monoclinic symmetry without contradicting the macroscopic cubic description of the crystal structure of BaTaO_2_N with space group $Pm\bar{3}m$ and found BaTaO_2_N to be most stable in the *Pmc*2_1_ (no. 26) structure type with an ordered anionic sublattice (Figure 3d).^[^ ^71^, ^78^, ^79^ ^]^ In contrast to the bonding of all occupied Ta–O states in *Pnma* and *I*4/*mcm*, antibonding interactions close to the Fermi level were observed in $Pm\bar{3}m$. This describes the increased bulk moduli of the cubic model because the Fermi level is forced even deeper into the antibonding region whenever pressure is applied (**Figure** **4**a).^[^ ^78^ ^]^ In fact, multiple possible orderings make it difficult to conduct systematic investigations using DFT calculations with predetermined elemental arrangements. According to Kaneko et al.,^[^ ^80^ ^]^ anion ordering in large supercells within perovskite‐type oxynitrides can be quickly predicted based on machine learning. Combined with the Metropolis Monte Carlo method, machine learning allows the exploration of the stable anion orderings of large supercells within perovskite‐type oxynitrides without costly DFT calculations.

The BaTaO_2_N stoichiometry is locally maintained with each Ta atom surrounded by two N and four O atoms in two possible local N/O orderings in the TaO_4_N_2_ octahedral structures: *cis* and *trans* configurations corresponding to the N−Ta−N bonds with 90° and 180° angles, respectively (Figure 4b).^[^ ^73^, ^79^ ^]^ Pair distribution function (PDF) analysis of the total neutron scattering reveals that although a long‐range anion order is not present in BaTaO_2_N, the TaO_4_N_2_ octahedra predominantly adopt a *cis*‐configuration with small Ta displacements toward the N atoms on a local scale.^[^ ^73^, ^81^, ^82^ ^]^ The *cis*‐chains propagate in all three dimensions in BaTaO_2_N, retaining the average cubic crystal symmetry.^[^ ^83^ ^]^ Nonetheless, the partial formation of a metastable *trans*‐type configuration by applying strain engineering can increase the N occupancy at axial sites.^[^ ^84^ ^]^ By applying an automated bonding analysis with Crystal Orbital Hamilton Populations (an orbital‐based technique), George et al.^[^ ^85^ ^]^ correlated the total energy of the systems with the strongest covalent interaction (Ta–N bond) of BaTaO_2_N and observed anti‐correlation with the bond energies of Ta–O, implying the importance of covalency of the Ta–N interactions in the anionic ordering of BaTaO_2_N. It was also found that the structure of BaTaO_2_N with a *cis*‐type local symmetry is much more stable than the corresponding *trans*‐type local symmetry.^[^ ^78^, ^79^ ^]^

A study on the influence of the cation size on the local atomic structure revealed that *cis*‐type N ordering and associated Ta displacements cooperate to stabilize local point charge dipole correlations among the TaO_4_N_2_ octahedra in BaTaO_2_N.^[^ ^86^ ^]^ Therefore, the Ta displacements significantly stabilize the structure of BaTaO_2_N, and the TaO_4_N_2_ tilting is minor. In the case of the Sr cation, the TaO_4_N_2_ octahedra are slightly tilted against each other, which is not observed for a larger Ba cation, promoting a stronger bonding in SrTaO_2_N than in BaTaO_2_N, and the occupied antibonding levels for the Ta−N bonds cannot be observed in BaTaO_2_N.^[^ ^87^ ^]^ Compared with an anion‐disordered cubic BaTaO_2_N ($Pm\bar{3}m$ (no. 221)), tetragonal BaTaO_2_N (*P*4/*mmm* (no. 123)) shows a site preference for oxide anions in the two opposite corners (along the *c*‐axis) of the TaO_4_N_2_ octahedra rather than the four‐square corners in the *ab* plane, leading to a distortion of the unit cell with the *c*‐axis being slightly longer than the *a*‐axis.^[^ ^88^ ^]^ Figure 4c,d shows a schematic representation of the band structure and DFT‐calculated band structure of BaTaO_2_N. The top of the valence band (highest occupied molecular orbital – HOMO) shifts upward due to the presence of hybridized N 2p and O 2p orbitals, while the bottom of the conduction band (lowest unoccupied molecular orbital – LUMO), which is composed of empty Ta 5d orbitals, remains unaffected.^[^ ^62^, ^89^ ^]^ By comparing the three different crystal structures of BaTaO_2_N, Bettine et al.^[^ ^90^ ^]^ found that the electronic and optical properties are strongly related to the TaO_4_N_2_ octahedral configurations, whereas the bandgap energy is influenced by the internal electric fields (polar versus non‐polar‐trans‐type orderings), which creates an asymmetry in the Ta–N bond lengths, and the minimum band gaps of the *P*4*mm*, *I*4/*mmm*, and *Pmma* structures were calculated to be 1.83 eV, 1.59 eV, and 1.49 eV, respectively. The density, separation, and transfer of charge carriers in oxynitrides depend on the nature and concentration of nonstoichiometric defects.^[^ ^91^ ^]^ Recently, Kousika and Thomas^[^ ^92^ ^]^ applied the Mott‐Littleton (*M*–*L*) method, which is commonly used for defect studies in molecular static calculations, rather than density functional theory (DFT) and molecular dynamics (MD) calculations to calculate the defect and migration energies of oxygen vacancies in BaTaO_2_N. It was found that BaTaO_2_N has a low oxygen vacancy defect energy (Δ*E*_M–L_ = 25.23 eV), which may reportedly improve its ionic conductivity, and the energy required for migration of oxygen for BaTaO_2_N is 2.42 eV. Although this study was mainly focused on calculating the defect and migration energies of oxygen vacancies, other prominent defects, such as nitrogen and cation vacancies, were not considered.

## 4 Dielectric, Ferroelectric, and Piezoelectric Properties of Perovskite BaTaO_2_N

**Figure 5:**
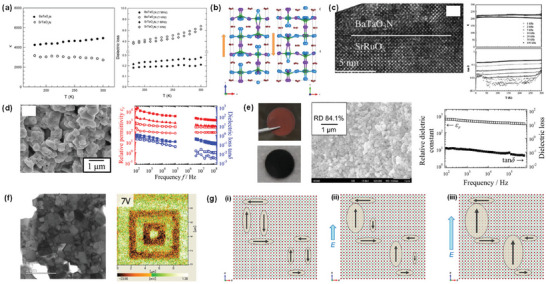
a) Temperature‐dependent dielectric permittivity and dielectric loss at 1 MHz and 1 kHz of BaTaO_2_N and SrTaO_2_N. Reproduced with permission.^[^ ^68^ ^]^ Copyright 2004 American Chemical Society. b) Displacements of atoms in BaTaO_2_N with opposite polarization. Reproduced with permission.^[^ ^96^ ^]^ Copyright 2012, American Chemical Society. c) high‐resolution TEM image of BaTaO_2_N/SrRuO_3_ interface and temperature‐dependent dielectric permittivity and dielectric loss of a 600‐nm‐thick BaTaO_2_N film. Reproduced with permission.^[^ ^97^ ^]^ Copyright 2007, American Chemical Society. d) SEM image of a BaTaO_2_N compact and relative permittivity and dielectric loss of BaTaO_2_N_0.85_ ceramics sintered at 1400 °C for 3 h. Reproduced with permission.^[^ ^99^ ^]^ Copyright 2016, Elsevier. e) Digital photographs, SEM image, and dielectric properties of BaTaO_2_N ceramics sintered by spark plasma method. Reproduced with permission.^[^ ^100^ ^]^ Copyright 2016, Elsevier. f) STEM and PFM images of BaTaO_2_N ceramics. Reproduced with permission.^[^ ^102^ ^]^ Copyright 2018, Elsevier. g) Polar nanoregions presenting the average $\mathrm{Pm}\bar{3}m$ cubic crystal lattice in which most of the O and N atoms are randomly distributed at 3*c* sites (i), polar nanoregions growing along the applied electric field (ii), and polarization saturated at applied electrical bias higher than ±60 V (iii). Reproduced with permission.^[^ ^103^ ^]^ Copyright 2019, American Chemical Society.

Along with the carrier concentration, a dielectric constant of semiconductors is a key parameter in determining the space charge layer, which can be applied to achieve the effective separation of photo‐excited charge carriers.^[^ ^93^ ^]^ Therefore, the dielectric properties of transition metal oxynitrides have been of particular interest. The frequency dependence of the dielectric response of BaTaO_2_N was studied using infrared reflection spectroscopy, and no infrared‐active polar soft mode, which is the origin of the ferroelectric property of oxides, was not observed, and the dielectric response was found not to reach very high values because the substitution of one nitrogen for oxygen cancels the tendency at displacement‐type instability.^[^ ^94^ ^]^ Despite its small octahedral tilt, BaTaO_2_N after the thermal treatment of the cold isostatically pressed sample at 1020 °C for 2 h exhibited an unexpectedly high bulk dielectric constant of ≈5000 at room temperature, which remained almost constant in the temperature range of 180–300 K (**Figure** **5**a).^[^ ^68^ ^]^ The high bulk dielectric constant was explained by the increased covalency of the Ta−N bonds, resulting in the off‐center displacement of the Ta cation through a second‐order Jahn−Teller distortion leading to a local polarization at each octahedron. Such high dielectric permittivity with a fairly weak temperature dependence was assumed to have a ferroelectric‐like behavior despite the absence of experimental evidence for its ferroelectricity. Further, different types of structural analysis were involved to understand the origin of the dielectric property of BaTaO_2_N. For instance, by applying extended X‐ray absorption fine‐structure (EXAFS) spectroscopy, Ravel et al.^[^ ^71^ ^]^ found that local structural distortions with very short correlation lengths are consistent with large dielectric permittivity of BaTaO_2_N. However, the difficulty with linking the dielectric property of BaTaO_2_N directly with the off‐center displacement of the Ta cation is that such compositional disorder (and associated polar behavior) is presumably frozen up until high temperature and hence not available to switch sign under the action of an applied electric field.^[^ ^72^ ^]^ Instead, Withers et al.^[^ ^72^ ^]^ correlated the relaxor‐type ferroelectric behavior of BaTaO_2_N with the formation of structurally frustrated one‐dimensional polar nanoregions (1D PNRs) along the ⟨001⟩ axis and suggested that the role of anion ordering is to set up random local strain fields suppressing transverse correlations of the inherent ⟨001⟩ chain dipoles and inhibiting the development of a long‐range ordered ferroelectric state. Similarly, Ziani et al.^[^ ^95^ ^]^ observed no paramount effect of anion ordering on the dielectric property of SrTaO_2_N based on a combined experimental and theoretical study. However, local anion ordering with *cis*‐type TaO_4_N_2_ was experimentally and theoretically confirmed for BaTaO_2_N. Using systematic first‐principles calculations, Hinuma et al.^[^ ^96^ ^]^ proposed a new mechanism that attributes the relaxor‐type ferroelectric behavior of BaTaO_2_N to the presence of low‐energy displacements having opposite polarization directions in the –Ta–N– coiled chain motif composed of the TaO_4_N_2_ octahedra with a *cis*‐type configuration (Figure 5b). By coupling the DFT calculations with the finite difference time domain (FDTD) simulations, Hafez et al.^[^ ^89^ ^]^ unraveled that BaTaO_2_N has large dielectric constants in the [001] direction based on the calculated real and imaginary parts of the diagonal components of the dielectric tensor ε^xx^, ε^yy^, and ε^zz^, which make it a promising dielectric material for various applications.

The BaTaO_2_N thin films grown epitaxially on (100)‐cut SrTiO_3_ by pulsed laser deposition exhibited dielectric permittivities ranging from 200 to 240 with a slight frequency dependence (Figure 5c).^[^ ^97^ ^]^ More than an order of magnitude difference noted in the dielectric permittivities of thin‐film (κ ≈ 220) and incompletely sintered ceramic (κ ≈5000) samples of BaTaO_2_N was attributed to the formation of boundary‐layer capacitors. Despite their differences in dielectric permittivity, both thin‐film and ceramic BaTaO_2_N samples do not undergo a structural or electrical phase transition, indicating the characteristics of ferroelectrics and relaxors. The X‐ray line broadening analysis indicated that BaTaO_2_N was formed with greater micro‐strains than its oxide analog KTaO_3_ due to the local atomic displacements, and the dielectric behavior of BaTaO_2_N was associated with the local polarization induced by geometry relaxation.^[^ ^98^ ^]^ The highly insulating BaTaO_2_N_0.85_ ceramic, which was obtained by Kikkawa and co‐workers^[^ ^99^ ^]^ via the synthesis of BaTaO_2_N by solid‐state reaction in NH_3_ at 1400 °C and further ammonolysis in the presence of BaCO_3_ additive, with a relative density of 73.0% showed real relative dielectric constants of *ε*_r_ = 620 at 10^2^ Hz and *ε*_r_ = 320 at 10^8^ Hz with the dielectric loss of less than 0.1, and it was found that porosity was necessary to eliminate the electronic contribution (Figure 5d). To avoid the partial loss of nitrogen from the perovskite lattice, the same research group^[^ ^100^ ^]^ applied a spark plasma sintering method with BaCN_2_ additive to synthesize a stoichiometric and electrically insulating BaTaO_2_N ceramic with a relative density of 79.8%, which showed relative dielectric constants in the range of *ε*_r_ = 320–650 and dielectric loss values in the range of tanδ = 0.04‐0.19 at room temperature (Figure 5e). Takeuchi et al.^[^ ^101^ ^]^ fabricated a thick BaTaO_2_N film with a relative density of 89% using an electrophoretic deposition and high‐temperature sintering with a B_2_O_3_ additive. The nitrogen loss was fully recovered after annealing in NH_3_, and the dielectric constant of 320 and the low dielectric loss of 0.05 at 1 MHz were achieved after nitridation for 100 h. Using piezoresponse force microscopy (PFM), Kikkawa and co‐workers also observed a piezoresponse for the polished slice of a dense BaTaO_2_N ceramic with a relative density of 93%, which increased up to 7 V and gradually decreased with time but persisted even after 150 min at 6 V (Figure 5f).^[^ ^102^ ^]^ Interestingly, highly porous BaTaO_2_N showed no piezoresponse because its porous surface was hydrolyzed. A weak piezoresponse was observed for an electrically conductive slice obtained from the deep inside of a dense BaTaO_2_N ceramic, suggesting that such a difference is linked to the existence of ferroelectricity. Cubic crystals of BaTaO_2_N with a size of up to 3.1 µm, grown in molten BaCN_2_, exhibited a much more highly insulating behavior than its ceramic counterpart and a ferroelectric piezoresponse that is attributable to the polar nanoregions induced by the polar linkages between *cis*‐type TaO_4_N_2_ octahedra (Figure 5g).^[^ ^103^ ^]^ This polarization switching of the self‐standing oxynitride perovskite without an electrical leakage was observed for the first time. According to Kikkawa and Masubuchi,^[^ ^104^ ^]^ ororhombic non‐centrosymmetric BaTaO_2_N with space group *Pmc*2_1_ can exhibit a second‐harmonic generat possibly have a ferroelectric domain structure. Despite the absence of polarization at room temperature due to the different arrangement directions of the *Pmc*2_1_ domain, it can still exhibit ferroelectric polarization after its poling treatment under an electric field exceeding the coercive field. In this case, the ferroelectricity of BaTaO_2_N can be explained in a classical way.^[^ ^105^ ^]^

The crystal and electronic structures of the BaTaO_2_N perovskite, presented in the previous two sections, provide valuable insight into its fundamental properties. By understanding its unique atomic arrangement and electronic configuration, a foundation for exploring the dielectric, ferroelectric, and piezoelectric properties of this material is established. In the next section, we delve into the synthesis techniques used to synthesize BaTaO_2_N perovskite, with the aim of taking advantage of its structural and electronic characteristics to achieve desirable functionalities. By combining knowledge of its crystal structure with the understanding of its dielectric, ferroelectric, and piezoelectric responses, we aim to further unlock the potential of BaTaO_2_N perovskite for various applications.

## 5 Synthesis of Perovskite BaTaO_2_N

### 5.1 High‐Temperature Synthesis of BaTaO_2_N

**Figure 6:**
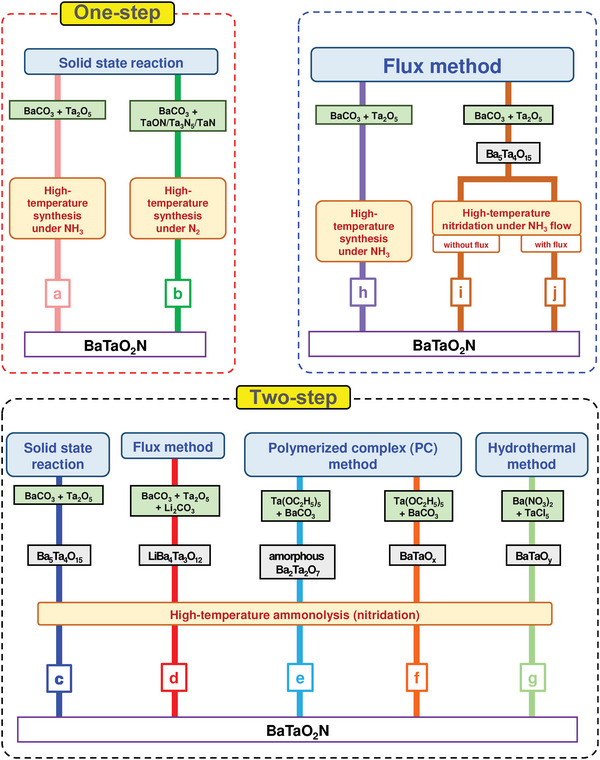
High‐temperature synthesis routes of perovskite BaTaO_2_N.

In addition to its dielectric, ferroelectric, and piezoelectric properties discussed above, the optical property,^[^ ^68^ ^]^ electrical conductivity,^[^ ^68^ ^]^ CO_2_ reduction,^[^ ^106^ ^]^ solar water splitting,^[^ ^107^ ^]^ photocatalytic degradation of organic water pollutants,^[^ ^108^ ^]^ and photobiocatalytic H_2_ evolution^[^ ^109^ ^]^ of perovskite BaTaO_2_N were also explored. Among them, photocatalytic and photoelectrochemical water splitting has been of particular research interest in the past decades. The photocatalytic activity and photoelectrochemical performance of BaTaO_2_N are known to be susceptible to its synthetic history. In the last three decades, various synthetic approaches, including one‐ and two‐step synthesis routes, have been developed to synthesize BaTaO_2_N. Perovskite BaTaO_2_N was first synthesized by a solid‐state reaction using BaCO_3_ and Ta_2_O_5_ at 1000 °C for a prolonged period of time under an NH_3_ flow by Marchand and co‐workers (**Figure** **6**a)^[^ ^69^, ^70^, ^110^, ^111^ ^]^:

(10) $$2\mathrm{BaC}O_{3}+{\mathrm{Ta}}_{2}O_{5}+2NH_{3}\to 2\mathrm{BaTa}O_{2}N+2CO_{2}+3H_{2}O$$

In addition to its nitriding role, NH_3_ also acts as a reducing agent, and the N_2_ atmosphere is, therefore, essential to prevent the reduction of Ta^5+^. As the tantalum and nitrogen sources, TaON, Ta_3_N_5_, or TaN were used to synthesize BaTaO_2_N under the N_2_ atmosphere (Figure 6b).^[^ ^112^, ^113^, ^114^ ^]^ Clarke et al.^[^ ^112^ ^]^ explored the use of high‐temperature conproportionation of binary oxides, nitrides, and oxynitrides under pure nitrogen gas in a radio‐frequency induction furnace to limit the oxide content of the final products and synthesized BaTaO_2_N using TaON at 1500 °C for 3 h under 1 atm N_2_. This method allowed approaching the thermodynamic limit with regard to crystallographic O/N order/disorder and concluded that the O/N disorder is thermodynamically favored in the perovskite structures (e.g., BaTaO_2_N) at high temperatures, while the O/N order is favored in the lower dimensionality K_2_NiF_4_‐type structures (e.g., Ba_2_TaO_3_N). Nie et al.^[^ ^113^ ^]^ succeeded in the synthesis of phase‐pure BaTaO_2_N with a homogeneous particle size distribution and a slightly off‐stoichiometric composition (BaTaO_1.86_N_0.49_□_0.65_) at 1200 °C for 5 h under NH_3_‐free atmosphere using BaCO_3_ and TaN as starting materials. By involving Ta_3_N_5_, Sun et al.^[^ ^114^ ^]^ further reduced the synthesis temperature and synthesized nearly single‐phase BaTaO_2_N particles with high homogeneity at 850 °C for 10 h under the N_2_ atmosphere.

BaTaO_2_N was also synthesized by a two‐step method, where i) the corresponding oxide precursor was first synthesized and ii) then subjected to high‐temperature ammonolysis under an NH_3_ flow for a prolonged period of time (e.g., 20 h, 30 h, etc.).^[^ ^67^, ^115^ ^]^ The oxide‐to‐oxynitride conversion also leads to the formation of porous structures through the lattice condensation process caused by the partial replacement of O^2−^ with N^3−^ in the anionic network.^[^ ^116^ ^]^ Unlike BaTaO_2_N, its crystalline oxide precursor does not have stoichiometry due to the larger Ba^2+^ ions, and Ba‐rich Ba_5_Ta_4_O_15_ has been routinely used as the oxide precursor to synthesize BaTaO_2_N by a two‐step method (Figure 6c).^[^ ^67^, ^117^ ^]^ Dong et al.^[^ ^118^ ^]^ significantly reduced the defect density and increased the surface area of BaTaO_2_N by involving Ba‐rich LiBa_4_Ta_3_O_12_ grown by a KCl flux method as a precursor (Figure 6d). However, the high‐temperature ammonolysis of a nonstoichiometric oxide leads to the formation of a byproduct (BaO), which must be removed after completing the high‐temperature ammonolysis, along with BaTaO_2_N due to the exclusion of one of the five Ba atoms from the unit cell of BaTaO_2_N.

(11) $${\mathrm{Ba}}_{5}{\mathrm{Ta}}_{4}O_{15}+4NH_{3}\to 4\mathrm{BaTa}O_{2}N+\mathrm{BaO}+6H_{2}O$$

In order to avoid the formation of BaO, BaTaO_2_N was alternatively synthesized by the high‐temperature ammonolysis of amorphous Ba_2_Ta_2_O_7_ (Figure 6e),^[^ ^65^ ^]^ BaTaO_x_ synthesized by a polymerized complex method (Figure 6f),^[^ ^115^ ^]^ and highly reactive BaTaO_y_ synthesized by a hydrothermal method (Figure 6g)^[^ ^119^ ^]^ with a stoichiometric Ba/Ta ratio of unity.

Generally, NH_3_ dissociates into hydrogen and nitrogen at temperatures as low as 500 °C,^[^ ^120^ ^]^ and the decomposition rate of NH_3_ depends on the nature of the surfaces where the NH_3_ molecules are adsorbed and the diffusion of NH_3_ on the surface.^[^ ^121^, ^122^ ^]^ Then, the formed hydrogen converts into a molecular state and removes oxygen as water vapor, while nitrogen forms an “active” species to be incorporated into the oxide lattice. Nitrogen in a molecular state has negligible activity during ammonolysis at <1000 °C because of its high dissociation enthalpy (Δ*H*_diss._ = 995 kJ mol^−1^ at 1027 °C). As the temperature increases, the ammonolysis of the oxide precursors becomes more prevalent with a greater formation rate of inert N_2_. Therefore, the synthesis of oxynitrides is only feasible with nonequilibrium conditions and proper control of the gas‐phase composition. Consequently, this forces a constant replenishing of NH_3_ and active nitrogen through either recirculation or regulation of gas flow. Hence, Brophy et al.^[^ ^123^ ^]^ studied the effects of key variables, including temperature, gas flow velocity, sample position, furnace configuration, etc., on the reaction rate and purity of BaTaO_2_N and found that the oxide precursors must be placed closer to the gas inlet to maximize the amount of active nitrogen and non‐dissociated NH_3_.

**Figure 7:**
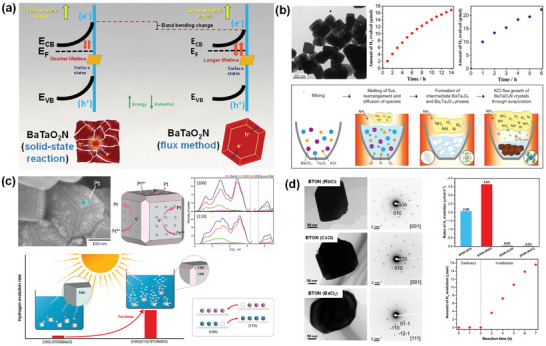
a) Schematic representation of the band structures of BaTaO_2_N synthesized by solid‐state reaction and flux method. Reproduced with permission.^[^ ^124^ ^]^ Copyright 2021, American Chemical Society. b) TEM image, formation mechanism, and visible‐light‐driven photocatalytic reaction time courses of H_2_ and O_2_ evolution of cube‐like BaTaO_2_N crystals. Reproduced with permission.^[^ ^129^ ^]^ Copyright 2015, American Chemical Society. c) SEM image and schematic representation of photodeposited Pt nanoparticles on the {100} facets of BaTaO_2_N, densities of states of different energy levels for {100} and {110} facets of BaTaO_2_N, hydrogen evolution rates of BaTaO_2_N with coexposed {100} and {110} and only {100} facets and diagram of electron−hole transfer in {100} and {110} facet junction. Reproduced with permission.^[^ ^131^ ^]^ Copyright 2019, American Chemical Society. TEM images, SAED patterns, and H_2_ evolution rates of i) BaTaO_2_N (RbCl), ii) BaTaO_2_N (CsCl), and iii) BaTaO_2_N (BaCl_2_), and BaTaO_2_N (BaCl_2_). Reproduced with permission.^[^ ^132^ ^]^ Copyright 2020, American Chemical Society.

The ammonolysis of the oxide precursors is the most commonly used method, where NH_3_ acts as a reduction agent and a nitrogen source. The oxide‐to‐BaTaO_2_N conversion process takes place at a high temperature under an NH_3_ flow for an extended period of time, generating various bulk and surface defects. Such defects are known to act as the recombination hubs, hindering the efficient separation and transfer processes of photo‐excited electrons and holes. In our previous works, we drastically reduced the synthesis time and defect density of BaTaO_2_N by applying an NH_3_‐assisted flux method, where NH_3_ was supplied by flowing from one end to the other of the horizontal tube furnace. In such a system, the transfer distance of active nitriding species (NH_2_, NH, N, etc.) becomes too far from a synthesis mixture even at a high gas flow rate, maximizing the generation of N_2_ and H_2_ before active nitriding species reach the surface of the synthesis mixture. Consequently, an insufficient supply of active nitriding species results in a higher defect density in BaTaO_2_N due to prolonged high‐temperature ammonolysis. In our recent work,^[^ ^124^ ^]^ we specifically localized an NH_3_ delivery system just above the synthesis mixture using a small‐diameter gas‐supplying horizontal tube to reduce the defect density of BaTaO_2_N, which generally results from long high‐temperature ammonolysis, by a fresh supply of more active nitriding species and non‐dissociated NH_3_. As a result, single‐phase BaTaO_2_N with low defect density and high crystallinity was synthesized at 950 °C for ≥6 and ≥4 h by solid‐state reaction and flux method, respectively, indicating the advantage of flux method over solid‐state reaction in this system (**Figure** **7**a).

However, the ammonolysis of the oxide precursors at high temperatures for an extended period of time leads to a self‐decomposition. The anion vacancies act as recombination centers for photo‐excited charge carriers and generate a large band bending at the solid‐liquid interface, forming a Schottky‐type barrier that hinders the prompt migration of electrons from the bulk to the surface reaction sites, which reduces the photocatalytic water splitting activity.^[^ ^62^ ^]^ The photocatalytic activity can be enhanced through the synthesis of semiconductor materials with a large surface area, high dispersion, high crystallinity, low defect density, defined morphology, and specifically exposed facets, allowing to reduce the number of recombination sites and increasing the photocatalytically active sites. Particularly, one‐ and two‐dimensional crystals can significantly enhance photocatalytic activity by increasing the active sites on the surface and decreasing the travel distance of photo‐excited charge carriers.^[^ ^125^ ^]^ However, the morphology control and the nanostructure fabrication of BaTaO_2_N are still challenging. Highly crystalline photocatalysts are preferred because the density of defects, which act as trapping sites and recombination centers for photo‐excited electrons and holes, can be reduced by improving the crystallinity. Also, the photocatalyst particles must be reasonably small (a few hundred nanometers) so that electrons and holes generated in the bulk of semiconductor material can more easily reach the surface.

The flux method (also known as the molten salt method) is one of the crystal growth techniques, allowing the growth of highly crystalline crystals free from thermal and mechanical constraints at lower temperatures, and supersaturation is the driving force for crystal growth. The flux growth is classified into three groups depending on the method of obtaining supersaturation: i) cooling the solution, ii) evaporating the flux, or iii) using a temperature gradient. It has been applied to grow high‐quality crystals with different morphologies, sizes, and surface features in supersaturated nonaqueous high‐temperature solutions.^[^ ^126^, ^127^, ^128^ ^]^ The flux method is not only beneficial for obtaining highly crystalline transition metal oxynitrides but also for improving the kinetics of the nitridation (Figure 6h–j). One of our previous studies on the influence of different types of fluxes, including KCl, KI, KF, MgCl_2_, CaCl_2_, SrCl_2_, BaCl_2_·2H_2_O, K_2_SO_4_, K_2_MoO_4_, and K_2_CO_3_, on the formation of BaTaO_2_N crystals revealed that the KCl flux could specifically induce the formation of highly crystalline cube‐like crystals of BaTaO_2_N with an average size of 125 nm and exposed {100} and {110} facets (Figure 7b). According to the results obtained from time‐ and temperature‐dependent experiments, the growth of cube‐like BaTaO_2_N crystals had possibly the following reaction steps^[^ ^129^, ^130^ ^]^: i) the decomposition of BaCO_3_ and dissolution of BaO and Ta_2_O_5_ in the KCl flux, ii) reactant diffusion through the molten KCl flux, iii) nucleation and growth of plate‐like BaTa_2_O_6_ and Ba_5_Ta_4_O_15_ crystals, iv) the dissolution of the BaTa_2_O_6_ and Ba_5_Ta_4_O_15_ crystals, and v) the crystallization and growth of cube‐like BaTaO_2_N crystals under an NH_3_ flow, as expressed by the following reactions:

(12) $${\mathrm{BaCO}}_{3}\to \mathrm{BaO}+{\mathrm{CO}}_{2}$$

(13) $$\mathrm{BaO}+{\mathrm{Ta}}_{2}O_{5}\to {\mathrm{BaTa}}_{2}O_{6}$$

(14) $$2\mathrm{BaT}a_{2}O_{6}+3\mathrm{BaO}\to {\mathrm{Ba}}_{5}{\mathrm{Ta}}_{4}O_{15}$$

(15) $$2Ba_{5}{\mathrm{Ta}}_{4}O_{15}+2\mathrm{BaT}a_{2}O_{6}+12N\to 12\mathrm{BaTa}O_{2}N+9O_{2}$$

Although cube‐like BaTaO_2_N crystals were still synthesized via the formation of intermediate oxide phases, the flux method significantly reduced the synthesis time to 10 h, and a molten KCl flux facilitated the growth of BaTaO_2_N crystals by creating a highly reactive environment because of its high mobility and volatility characteristics. In comparison to BaTaO_2_N modified with 1.5 wt% IrO_2_ and 0.3 wt% Pt nanoparticles,^[^ ^107^ ^]^ cube‐like BaTaO_2_N crystals modified with Pt and CoO_x_ nanoparticles showed relatively higher H_2_ and O_2_ evolution rates, respectively, due to the reduced defect density and higher crystallinity achieved by an NH_3_‐assisted flux method using KCl (Figure 7b).

**Figure 8:**
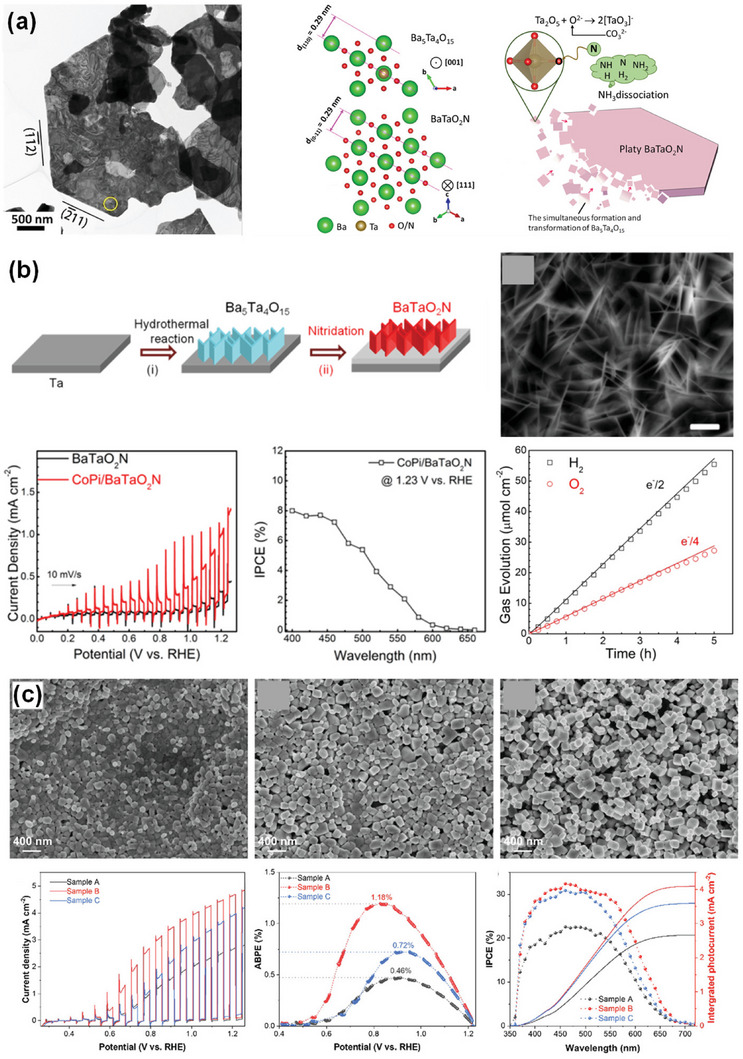
a) TEM image of BaTaO_2_N grown using K_2_CO_3_−KCl binary flux with a molar ratio of 20/80 at 950 °C for 8 h, crystal structures of Ba_5_Ta_4_O_15_ viewed from the [00$\bar{1}$] direction (top) and BaTaO_2_N viewed from the [111] direction (bottom), and schematic illustration of the formation mechanism of platy BaTaO_2_N crystals. Reproduced with permission.^[^ ^134^ ^]^ Copyright 2020, American Chemical Society. b) Synthesis procedure and top‐view SEM image of BaTaO_2_N film, current−potential curves of bare and CoPi‐covered BaTaO_2_N electrodes, IPCE of CoPi/BaTaO_2_N measured at 1.23 V versus RHE, and H_2_ and O_2_ evolution from a CoPi/BaTaO_2_N photoanode at 1.23 V versus RHE in a 0.5 M potassium phosphate solution (pH 13) under AM 1.5G simulated sunlight. Reproduced with permission.^[^ ^66^ ^]^ Copyright 2016, American Chemical Society. c) SEM images of BaTaO_2_N nanoparticle films with different Ba:Ta atomic ratios on Nb substrates, LSV curves of BaTaO_2_N photoanodes with NiCoFe‐Bi co‐catalyst in 1 M KOH (pH 13.6) under simulated AM 1.5G simulated sunlight, ABPE of the BaTaO_2_N photoanodes, and IPCE spectra of the BaTaO_2_N photoanodes at 1.23 V versus RHE and the corresponding integrated photocurrent over the standard AM 1.5G solar spectrum. Reproduced with permission.^[^ ^148^ ^]^ Copyright 2022, Elsevier.

In comparison to the NaCl flux, which led to the formation of an almost perfect cubic structure with six isotropic {100} facets, the KCl flux resulted in a similar cubic‐like structure with exposed smooth edges belonging to the {110} facets at the intersection of the {100} facets. It was revealed that the photo‐excited electrons and holes separately transfer to the {100} and {110} facets of BaTaO_2_N because of the different energy levels of the {100} and {110} facets, enhancing the charge separation through the inter‐crystalline charge separation between the two facets (Figure 7c).^[^ ^131^ ^]^ The {100} facet‐dominant BaTaO_2_N crystals were grown by a flux method using RbCl and CsCl, whereas the tetrahedral‐shaped BaTaO_2_N crystals with exposed {111} and {100} facets were grown using the BaCl_2_∙2H_2_O flux (Figure 7d).^[^ ^132^ ^]^ As a result, the BaTaO_2_N crystals grown using the RbCl flux exhibited a significantly higher photocatalytic H_2_ evolution rate in comparison to those grown using the CsCl and BaCl_2_∙2H_2_O fluxes. The BaTaO_2_N crystals with differently exposed facets were formed because of changes in the electrostatic forces induced by the involved fluxes. The flux exhibits an ionic state at higher temperatures, in which the cations and anions are adsorbed on the surfaces of the precursor particles to form an electric double layer between the precursor particles and the molten salts. The Cl^−^ anions exhibit larger negative adsorption energy than the alkali cations, resulting in more facile adsorption on the surface, which induces a negative surface charge on BaTaO_2_N. These surface negative charges are then neutralized by the adsorption of alkali cations on the surface. The strength of the repulsion force between the particles due to the surface negative charges is therefore determined by the amount of adsorbed alkali cations. However, the adsorption energy of the alkali cation decreases as the size of the alkali cation increases, implying that smaller cations can be adsorbed more easily on the BaTaO_2_N surface to decrease the repulsion force and promote the aggregation of BaTaO_2_N particles. As Cs^+^ (0.18 nm) is slightly larger than Rb^+^ (0.17 nm), the BaTaO_2_N particles grown in the CsCl flux have a greater repulsion force than those grown in the RbCl flux. The increased repulsion force suppresses the aggregation of the BaTaO_2_N particles, leading to the formation of additional edge steps in the BaTaO_2_N (CsCl) samples. In the case of the BaTaO_2_N (BaCl_2_) samples, Ba^2+^ (0.16 nm) is smaller than both Rb^+^ (0.17 nm) and Cs^+^ (0.18 nm), thereby resulting in the increased adsorption of Ba^2+^ cations on the surfaces of the BaTaO_2_N particles. Since barium is one of the constituent elements of BaTaO_2_N, excess Ba^2+^ adsorption on the surfaces of the growing BaTaO_2_N particles can affect the atomic arrangement of BaTaO_2_N, leading to the exposure of different crystal facets.^[^ ^132^ ^]^ Due to its cubic crystal structure, BaTaO_2_N commonly crystallizes into a cubic shape even when Ba_5_Ta_4_O_15_ with plate‐like structures^[^ ^129^ ^]^ is involved as an intermediate oxide phase. Nevertheless, because of a small lattice mismatch (0.7%) in the atomic arrangements of the Ba_5_Ta_4_O_15_ (001) plane and the BaTaO_2_N (111) plane, the plate‐like submicron‐sized structures of BaTaO_2_N were directly synthesized by a KCl flux‐mediated ammonolysis.^[^ ^133^ ^]^ Similarly, Luo et al.^[^ ^134^ ^]^ also synthesized platy BaTaO_2_N crystals with well‐developed {111} facets via the simultaneous formation and transformation of Ba_5_Ta_4_O_15_ using the K_2_CO_3_/KCl binary flux (**Figure** **8**a). An amorphous layer is often formed on the surface of BaTaO_2_N during the high‐temperature ammonolysis process, which promotes the recombination of photo‐excited electrons and holes, thus reducing its performance. Therefore, the post‐synthesis thermal treatment of BaTaO_2_N particles in an Ar or H_2_ atmosphere and the necking treatment were found to be advantageous for enhancing its performance.^[^ ^65^, ^124^, ^135^ ^]^ Although the flux method is one of the promising synthesis routes for BaTaO_2_N with a lower defect density, the flux residue must be removed in order to reduce its negative impact on the performance of BaTaO_2_N.

### 5.2 Other Methods Applied for the Synthesis of Perovskite BaTaO_2_N

High‐temperature synthesis of perovskite oxynitrides under an NH_3_ flow for a prolonged period of time causes the reduction of transition metal cations to lower oxidation state species for charge compensation of the nitrogen vacancies. Those reduced species form a donor level just below the conduction band minimum, which may act as recombination centers for photo‐excited electrons and holes, thus decreasing its performance. Accordingly, much effort has been made to reduce the defect density of transition metal oxynitrides by developing novel strategies.^[^ ^136^ ^]^ Particularly, low‐temperature routes that use amide, urea, and azide as nitrogen sources instead of ammonia gas have been demonstrated to be alternative methods for the synthesis of transition metal oxynitrides with a lower defect density. The use of NaNH_2_ with different NaNH_2_/Ta molar ratios as a flux‐nitrogen source promoted the synthesis of BaTaO_2_N even at 493 K for 20 h in a tightly sealed autoclave, which is ≈500 K lower than the temperature applied generally to synthesize BaTaO_2_N.^[^ ^137^ ^]^ However, the nature of an explosive reaction was not described in detail. The highest photocatalytic H_2_ and O_2_ evolution rates were observed for BaTaO_2_N synthesized with the NaNH_2_/Ta molar ratio of 3 because of its higher crystallinity and lower defect density associated with the reduced Ta^3+^ species. Odahara et al.^[^ ^138^ ^]^ applied a similar synthesis approach using Ba(OH)_2_, TaCl_5_, and NaNH_2_, which caused an explosive reaction and resulted in snuff powders as the products. Nevertheless, the addition of hexane reduced the risk of explosion during the mixing of starting materials and enabled to control of this exothermic reaction, leading to the formation of BaTaO_2_N:

(16) $$\mathrm{Ba}{\left(\mathrm{OH}\right)}_{2}+{\mathrm{TaCl}}_{6}+5\mathrm{NaN}H_{2}\to {\mathrm{BaTaO}}_{2}N+5\mathrm{NaCl}+4NH_{3}$$

Cordes et al.^[^ ^139^ ^]^ synthesized BaTaO_2_N by an ammonothermal method at temperatures of 900 K and maximum pressures of up to 300 MPa in high‐pressure custom‐built autoclaves using Ta and Ba metals and NaN_3_ and NaOH as mineralizers. Intermediates with amide, imide, and hydroxide groups were assumed to be the most likely reactive species in the formation reaction of BaTaO_2_N. Conventional ammonothermal synthesis of oxynitrides requires an alloy precursor prepared by arc melting,^[^ ^140^ ^]^ which can lead to the volatilization of low‐boiling‐point components. Thus, a direct synthesis by a one‐pot method is advantageous. Yoshimura and co‐workers^[^ ^141^ ^]^ successfully synthesized BaTaO_2_N by an ammonothermal method at >600 for 20 h under a pressure of 100 MPa using supercritical ammonia, NaOH as an oxygen source, and NaNH_2_ as a basic mineralizer to improve the solubility of starting materials. However, perovskite oxynitrides are unstable during high‐temperature synthesis and release a portion of their lattice nitrogen at >900 °C. Therefore, the use of nitrogen‐rich starting materials to intermittently supply nitrogen to the reaction system can assist to avoid a partial nitrogen loss from the formed oxynitrides during the high‐temperature synthesis.

Alkaline earth metal cyanamides are compounds containing alkaline earth metals and nitrogen together as their constituents like oxynitride perovskites. They are expected to melt at temperatures lower than the temperature for the nitrogen release from perovskite oxynitrides. Since the partial decomposition of SrTaO_2_N begins at 950 °C, which is ≈100 °C higher than the decomposition temperature of BaTaO_2_N, SrTaO_2_N was chosen to be partially soluble in the melt of metal cyanamide and recrystallizes in cooling. Therefore, the molten BaCN_2_ partially dissolved SrTaO_2_N and formed a solid‐solution precipitate Sr_1−x_Ba_x_TaO_2_N from the melt upon cooling. The obtained products had a compositional gradient from a strontium‐rich interior to a barium‐rich exterior in their crystals.^[^ ^142^ ^]^ Hosono et al.^[^ ^143^ ^]^ found that *t*‐BaNCN is more stable than *r*‐BaNCN, and it melts at 1183 K, making it a promising flux for the crystal growth and sintering of various (oxy)nitrides. Alkaline earth metal carbodiimides are ionic crystals comprising metal cations and symmetric [N = C = N]^2−^ anions, which are different from asymmetric cyanamide anions ([N = C (three bonds N]^2−^). These compounds can act as fluxes for the synthesis of perovskite oxynitrides. Kikkawa and co‐workers^[^ ^103^ ^]^ synthesized small cubic crystals (3.1 µm) of perovskite BaTaO_2_N via the formation of a Ruddlesden−Popper‐type oxynitride from the reaction between BaTaO_2_N powder and molten BaCN_2_ flux. On the other hand, BaCN_2_ was also used as a starting material along with Ta_2_O_5_ to synthesize highly dispersed BaTaO_2_N particles with a size of 100–850 nm at ≈900 °C for 5 h because of the partial dissolution of BaTaO_2_N in the BaCN_2_ melt.^[^ ^144^ ^]^ Further, to prevent the thermal decomposition and the partial nitrogen loss, spark plasma sintering with (100 MPa) and without pressure was applied to rapidly synthesize BaTaO_2_N powder for 3 and 10 min using BaCN_2_ and urea as additive and nitrogen sources, respectively.^[^ ^100^, ^145^ ^]^ The BaTaO_2_N grains were bonded through dissolution and precipitation at their surfaces in the BaCN_2_ melt, and a minor Ba‐rich compound, possibly Ruddlesden‐Popper‐type Ba_2_TaO_3_N, was identified at the boundaries of the BaTaO_2_N grains.^[^ ^100^ ^]^ BaTaO_2_N synthesized by a pressureless spark plasma sintering technique exhibited a very high room‐temperature relative permittivity up to 9550 with a dielectric loss down to 0.001 at 100 Hz due to its O/N ordering in the *cis* configuration and high purity (97.8%).^[^ ^145^ ^]^ Gomathi et al.^[^ ^146^ ^]^ also employed urea as a nitrogen source to synthesize BaTaO_2_N nanoparticles with a size of about 60 nm at 1226 K for 2 h. NH_3_ is yielded upon urea decomposition at 523 K and can react with starting materials to form oxynitrides. By varying the amount of urea, the nitrogen stoichiometry can, therefore, be controlled since urea gives a nitrogen content close to the theoretical value. However, urea generally decomposes at a relatively low temperature that is far below the temperature required for the formation of perovskite oxynitrides and the ammonolysis of their oxide precursors. Therefore, it is still challenging to precisely control the synthesis process, purity, and stoichiometry of BaTaO_2_N. The presented synthesis approaches generally result in BaTaO_2_N powders, which need to be further deposited on substrates for PEC OER. The PEC‐OER performance depends on the thickness, morphology, crystallinity, grain boundaries, etc. of the BaTaO_2_N films.

## 6 Fabrication of BaTaO_2_N films

**Figure 9:**
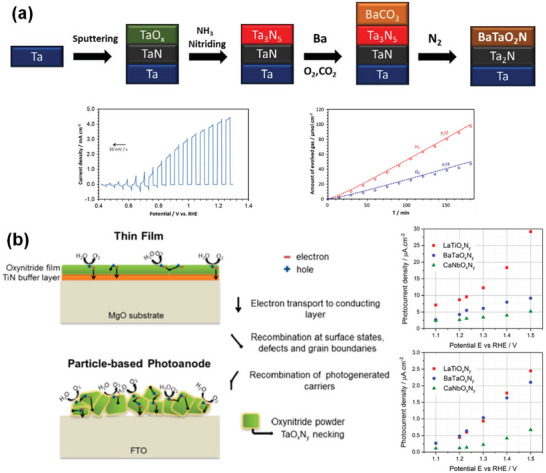
a) Schematic representation of thin‐film fabrication, I–V curves of a BaTaO_2_N/Ta_2_N/Ta thin‐film electrode, and H_2_ and O_2_ evolution from a CoO_x_‐deposited BaTaO_2_N/Ta_2_N/Ta photoanode held at 1.0 V versus RHE in an aqueous 0.1 M potassium phosphate solution (pH 13) under AM 1.5G simulated sunlight. Reproduced with permission.^[^ ^149^ ^]^ Copyright 2020, WILEY‐VCH Verlag GmbH & Co. KGaA. b) Schematic representation of thin film‐ and particle‐based oxynitride photoanodes and their photoelectrochemical performance. Reproduced with permission.^[^ ^151^ ^]^ Copyright 2019, American Chemical Society.

The design of devices for practical applications typically requires high‐quality thin films. The fabrication of thin films is an attractive approach for the synthesis of dense and highly crystalline perovskite oxynitrides. The epitaxial thin film of BaTaO_2_N was grown on a 100‐cut SrTiO_3_ single‐crystal substrate with a conducting buffer layer of SrRuO_3_ by a pulsed laser deposition technique at 760 °C in a mixed gas atmosphere of 100 mTorr N_2_/O_2_.^[^ ^97^ ^]^ The dielectric permittivity of the fabricated thin film was much different from the value reported for incompletely sintered ceramics of BaTaO_2_N with considerable porosity (45%).^[^ ^68^ ^]^ It was found that the epitaxial strain effect can cause a tetragonal distortion of the unit cell of perovskite BaTaO_2_N with a negligible volume change. The nanostructured thin films of BaTaO_2_N were formed on Ta substrates by the ammonolysis of hydrothermally grown Ba_5_Ta_4_O_15_ nanosheet layers at 1000 °C for 2 h.^[^ ^66^ ^]^ The fabricated BaTaO_2_N thin film deposited with a CoPi layer exhibited a photocurrent density of ≈0.75 mA cm^−2^ at 1.23 V versus RHE and generated O_2_ for 5 h with a Faradaic efficiency of > 90% without significant deactivation under AM 1.5G simulated sunlight due to the high crystallinity of the formed film (Figure 8b). Although the branching structure of the BaTaO_2_N film could promote the transfer of photo‐excited holes to the electrode surface by shortening the diffusion distance, the pores of the fabricated film would hamper the efficient transfer of photo‐excited electrons to the back contact. Therefore, the photocurrent at low applied potential was not significantly enhanced. The BaTaO_2_N thin film was fabricated by the ammonolysis of the oxide precursor film, which was deposited on the alumina substrate by the dip coating of a gel made by a polymerizable complex method, at 950 °C for 15 h.^[^ ^147^ ^]^ The BaTaO_2_N thin film was fabricated by depositing BaF_2_ and Ta_2_O_5_ on an Nb foil by a dual‐source electron‐beam deposition, followed by ammonolysis at 1273 K for 10 h under an NH_3_ flow.^[^ ^148^ ^]^ With increasing the Ba:Ta ratio, the size of nanoparticles in the BaTaO_2_N film increased and the shape of nanoparticles became well‐defined, indicating the improved crystallinity of less densely packed BaTaO_2_N nanoparticles. The BaTaO_2_N nanoparticle photoanode achieved a photocurrent density of 4.7 mA cm^−2^ at 1.23 V versus RHE under AM 1.5G simulated sunlight and a maximum ABPE of 1.18% (Figure 8c). The BaTaO_2_N/Ta_2_N/Ta thin film was fabricated by depositing the TaN, Ta_3_N_5_, and BaCO_3_ layers on the Ta metal substrate by radio frequency (FR) sputtering and heat treatment at 1173–1273 K for 0.5 to 1 h under N_2_ atmosphere.^[^ ^149^ ^]^ CoO_x_‐deposited BaTaO_2_N/Ta_2_N/Ta electrodes produced a photocurrent density of 4.6 mA cm^−2^ at 1.23 V versus RHE and exhibited a 9% IPCE at 600 nm during water oxidation under AM 1.5G simulated sunlight due to the remarkable conductivity of Ta_2_N, promoting an efficient transfer of electrons between BaTaO_2_N and Ti layers, and both H_2_ and O_2_ were produced with a Faradaic efficiency of almost 100% (**Figure** **9**a). In contrast, the layer of densely‐packed polyhedral crystals of BaTaO_2_N was directly grown on the Ta metal substrate by a flux‐coating technique, and no intermediate TaO_x_ byproduct layer was found, indicating high‐quality crystal layers with interfaces.^[^ ^150^ ^]^

Lippert and co‐workers^[^ ^151^ ^]^ comparatively studied the photoelectrochemical performance of oxynitride, including BaTaO_x_N_y_, thin film‐ and particle‐based photoelectrodes fabricated by conventional pulsed laser deposition (PLD) method and electrophoretic deposition, respectively. Interestingly, they found that the particle‐based photoelectrodes could exhibit a higher photocurrent density due to the improved absorption properties and a larger electrochemical surface area (Figure 9b). The thin film‐based photoelectrodes could surpass the particle‐based photoelectrodes because of higher crystallinity and good electrical contact between grains, facilitating the separation and transfer of photo‐excited charge carriers. The growth of crystalline and fully dense films on three‐dimensional nanostructures was suggested to widen the electrochemical surface area of the films while preserving the bulk properties. One of the challenging issues of oxynitride‐based semiconductors is physicochemical degradation at the interface with water, where the electrochemical reaction takes place. To understand the degradation mechanism and to find strategies to mitigate such detrimental effects, Pergolesi et al.^[^ ^152^ ^]^ suggested that the study of solid–liquid interface can benefit enormously from the use of thin films with well‐defined and atomically flat surfaces for synchrotron‐based surface‐sensitive X‐Ray scattering methods and neutron reflectometry. Particularly, synchrotron radiation‐based soft X‐ray angle‐resolved photoemission spectroscopy (SX‐APRES) enables the probing of the *k*‐resolved electronic structure in the subsurface region despite atmospheric surface contamination and provides the opportunity to explore materials, surfaces, and interfaces as well as their reactivity and evolution. The anion arrangement in oxynitrides significantly influences their optical and electronic properties. However, it is difficult to assess anion arrangement in thin films. Yamamoto et al.^[^ ^153^ ^]^ demonstrated inverse photoelectron holography, which is an atomic‐resolution holography technique, to directly measure the local structure around atoms of light elements. The obtained holograms of anions in the SrTaO_2_N thin films showed a remarkable difference between O and N holograms, revealing a *trans*‐type anion ordering. The simulated hologram agrees well with the experimentally obtained data. To achieve high efficiency in BaTaO_2_N film, it is necessary to select an appropriate deposition technique and optimize the deposition parameters.

## 7 Control of Particle Morphology, Size, Porosity, and Surface Properties of BaTaO_2_N

**Figure 10:**
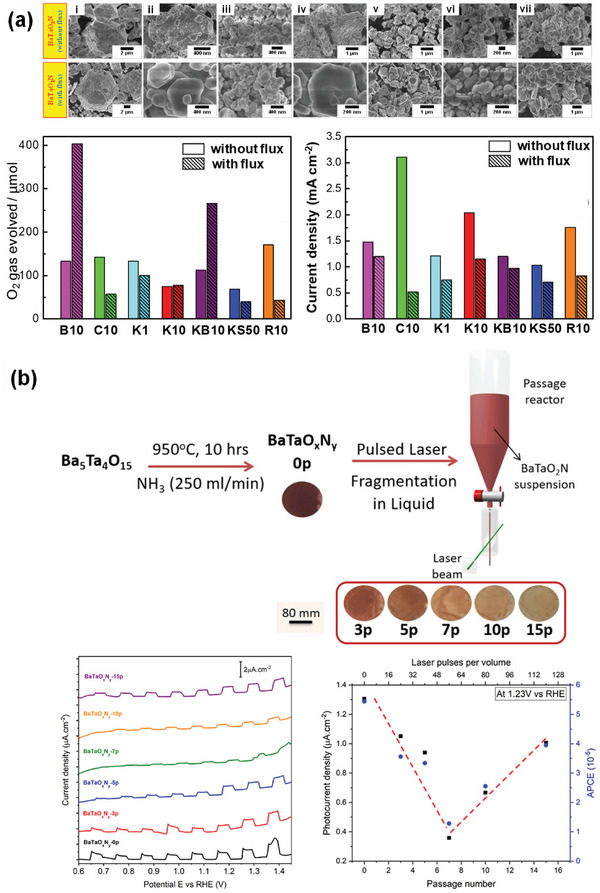
a) SEM images of BaTaO_2_N crystal structures obtained by ammonolysis of the flux‐grown Ba_5_Ta_4_O_15_ crystals, grown using BaCl_2_ and a 10 mol% sol. conc. (*B10*), CsCl and a 10 mol% sol. conc. (*C10*), KCl and a 1 mol% sol. conc. (*K1*), KCl and a 10 mol% sol. conc. (*K10*), KCl + BaCl_2_ and a 10 mol% sol. conc. (*KB10*), K_2_SO_4_ and a 50 mol% sol. conc. (*KS50*), and RbCl and a 10 mol% sol. conc. (*R10*), at 950 °C for 20 h with (*bottom row*) and without (*top row*) KCl flux, and the amount of photocatalytically evolved oxygen (first 3 h) and photocurrent density at 1.2 V versus RHE. Reproduced with permission.^[^ ^117^ ^]^ Copyright 2019, The Royal Society of Chemistry. b) Schematic representation of the synthesis of BaTaO_x_N_y_ powders and laser fragmentation process, potentiodynamic scans for the BaTaO_x_N_y_‐zp photoanodes in NaOH electrolyte under chopped light illumination, and photocurrent density and APCE values at 1.23 V versus RHE as a function of the number of passages applied for fragmentation. Reproduced with permission.^[^ ^156^ ^]^ Copyright 2020, Elsevier.

Controlling the particle morphology, size, and dimension, porosity, and surface properties is essential for improving the water‐splitting performance of BaTaO_2_N. Previously, the particle morphology, size, and porosity of BaTaO_2_N were modulated by the ammonolysis of the Ba_5_Ta_4_O_15_ precursor, synthesized by a flux method using BaCl_2_, KCl, RbCl, CsCl, KCl+BaCl_2_, and K_2_SO_4_ with different solute concentrations, with and without KCl flux.^[^ ^117^ ^]^ The flux method enabled to control the morphology, size, and dimension of Ba_5_Ta_4_O_15_ crystals. It was found that the ammonolysis of Ba_5_Ta_4_O_15_ with the KCl flux could lead to the formation of the dense crystals of BaTaO_2_N, whereas the ammonolysis of Ba_5_Ta_4_O_15_ without the KCl flux resulted in the porous structures of BaTaO_2_N (Figure 6i). Among the samples with different morphologies, sizes, and porosities, the BaTaO_2_N crystal structures obtained by a flux‐free ammonolysis of Ba_5_Ta_4_O_15_ crystals grown using CsCl at a 10 mol% solute concentration (sample C10) exhibited the highest photocurrent density of ≈3.11 mA cm^−2^ at 1.2 V versus RHE due to their average size of 1–2 µm and a porous surface. A further increase and a decrease in the particle size led to a reduction in the photocurrent density due to the existence of a long transfer distance to reach the surface for photo‐excited charge carriers and the surface degradation by excess high‐temperature nitridation, increasing the bulk and surface recombination rates, respectively. Within the first 3 hours of the photocatalytic reaction, BaTaO_2_N obtained by a flux‐inclusive ammonolysis of Ba_5_Ta_4_O_15_ crystals grown using BaCl_2_ at a 10 mol% solute concentration (sample B10K) showed a higher O_2_ evolution rate of 134.64 µmol h^−1^ (404 µmol) because of its larger particle size, predominantly exposed surfaces with a similar nature, high crystallinity, reduced surface defect density, and fewer grain boundaries (**Figure** **10**a).

According to Anbalagan and Thomas,^[^ ^154^ ^]^ the sintering of particles with a diameter of <10 nm requires no external sintering aids, including the addition of barium sources, and can lead to the formation of a cluster of tantalum and oxygen atoms at the interface of the BaTaO_2_N particles. Another study based on molecular dynamics on size and temperature‐dependent specific heat capacity and diffusion constants of ultra‐small BaTaO_2_N nanoparticles revealed that specific heat capacity increases with temperature, but with additional features that become pronounced with a size reduction due to the surface contribution and the formation of vacancies.^[^ ^155^ ^]^ The diffusion constant decreases with increasing the size of BaTaO_2_N nanoparticles. These affect the defect chemistry, phase and interface stability, and sintering dynamics of BaTaO_2_N nanoparticles. Haydous et al.^[^ ^156^ ^]^ studied the effect of downstream laser fragmentation, a novel method to increase surface area, on the specific surface area and photoelectrochemical performance of barium tantalum oxynitride (Figure 10b). Despite the increase in surface area, the performance of BaTaO_2_N photoanodes did not improve significantly and the absorbed photon‐to‐current conversion efficiency (APCE) decreased within the first fragmentation passages due mainly to the presence of grain boundaries in the fragmented BaTaO_2_N particles, which became recombination hubs for photo‐excited electrons and holes. Also, the laser fragmentation resulted in the loss of N content and reduced crystallinity of BaTaO_2_N, which significantly affected the optical properties and photoelectrochemical performance. In general, the surface area becomes greater when the particle size is minimized. The reduced particle size may give a quantum size effect, resulting in a broader band gap. Therefore, a high degree of crystallinity is often preferred over a large surface area for uphill water splitting reaction because of the recombination of photo‐excited charge carriers in the defects.^[^ ^157^ ^]^

The water‐splitting performance of photocatalysts is sensitive to the surface local structures. Recent studies on the effect of different exposed surfaces found that the BaTaO_2_N crystals with well‐developed {111} facets^[^ ^134^ ^]^ and co‐exposed {100} and {110} facets^[^ ^131^ ^]^ could exhibit a significantly enhanced photocatalytic activity for H_2_ evolution in comparison to the BaTaO_2_N crystals with only {100} facets. Hence, a number of theoretical studies have been conducted to understand the impact of the surface local structures on various properties of BaTaO_2_N. The influence of different surface terminations on the electronic, optical, and photocatalytic properties of *trans*‐ and *cis*‐BaTaO_2_N was studied using DFT calculations by Zhou et al.^[^ ^158^ ^]^ The calculated work functions showed that the Ba‐terminated surfaces have smaller work functions than the Ta‐terminated ones. This indicates that the former is more favorable to promoting the separation of photo‐excited charge carriers. The hydrogen evolution reaction proceeds more easily on the surfaces terminated with Ta, O, and N atoms, while the oxygen evolution reaction proceeds more readily on the surfaces terminated with N atoms than on the surfaces terminated with O atoms. In another work,^[^ ^159^ ^]^ they also found that the water dissociation prefers to occur on the BaNbO_2_N surface terminated with N atoms than on the surface terminated with O atoms. Dissociative water adsorption on surfaces with the (100) and (001) terminations is thermodynamically favorable. Therefore, the exposed O atoms (*cis*‐(100)‐Nb_2_O_3_N, *trans*‐(100)‐NbO_2_, and *trans*‐(001)‐BaO) are the most favorable sites for HER, while *cis*‐(001)‐BaO is the most suitable for OER.

**Figure 11:**
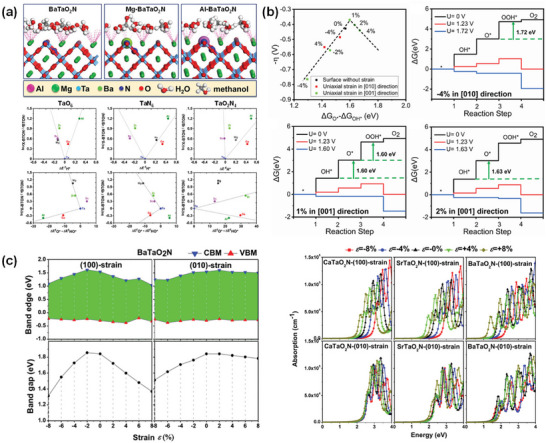
a) Close contacts of TaO_4_N_2_‐terminated (110) surfaces of BaTaO_2_N, Mg‐BaTaO_2_N, and Al‐BaTaO_2_N, (*top*) ln[(*r*_XBTON_)/(*r*_BTON_)] versus (Δ*E* ^r^_H*_) plots for H_2_ evolution of pristine and cation‐doped BaTaO_2_N photocatalysts with surfaces terminated with TaO_6_, TaN_6_, and TaO_4_N_2_ octahedra. Adsorbed intermediate is H*, and (*bottom*) ln[(*r*_XBTON_)/(*r*_BTON_)] versus (Δ*E* ^r^_O*_ – Δ*E* ^r^_HO*_) plot for O_2_ evolution of pristine and cation‐doped BaTaO_2_N photocatalysts with surfaces terminated with TaO_6_, TaN_6_, and TaO_4_N_2_ octahedra. Adsorbed intermediates are O* and HO*. Reproduced with permission.^[^ ^133^ ^]^ Copyright 2022, American Chemical Society. b) Volcano plot of the free‐energy difference of (Δ*G*_O*_ – Δ*G*_OH*_) and the OER theoretical overpotential (η) for TaO_2_N‐terminated (100) full O‐covered surfaces. Gibbs free energy diagrams for the full O‐covered TaO_2_N‐terminated (100) surface with 4% compressive uniaxial strain in the [010] direction, 1% tensile uniaxial strain in the [001] direction, and 2% tensile uniaxial strain in the [001] direction. Reproduced with permission.^[^ ^160^ ^]^ Copyright 2021, American Chemical Society. c) Effect of strain on band edges and band gaps of ATaO_2_N (A = Ca, Sr, and Ba) and their optical absorption under strain. Reproduced with permission.^[^ ^161^ ^]^ Copyright 2019, Springer Nature AG & Co. KGaA.

Molecular dynamics (MD) simulation was involved to gain insights into the respective effect of doping with various aliovalent cations on the adsorption energy of water molecules and formed intermediates (H* for H_2_ evolution and HO*, O*, and HOO* for O_2_ evolution) on the predominant (110) surface of BaTaO_2_N (BTON) terminated with TaO_6_, TaN_6_, and TaO_4_N_2_ octahedra (**Figure** **11**a).^[^ ^133^ ^]^ The kinetic V‐type plots in H_2_ evolution and the linear energy−performance correlations in O_2_ evolution were observed using the relative kinetic analysis (ln[(*r*_XBTON_)/(*r*_BTON_)] versus ‐(*E*_XBTON_−*E*_BTON_)) plot. The experimental photocatalytic reaction rates were satisfactorily described using the adsorption energies of intermediates (H* for HER and HO* and O* for OER) estimated by MD calculations, rationalizing the effects of aliovalent cation doping (Al^3+^, Ga^3+^, Mg^2+^, Sc^3+^, and Zr^4+^) and surface chemical termination (TaO_6_, TaN_6_, and TaO_4_N_2_) of BaTaO_2_N. A satisfactory match for all dopants was obtained for the BaTaO_2_N surface terminated with the TaO_6_ octahedra. Nevertheless, the BaTaO_2_N:Zr surface terminated with the TaO_4_N_2_ octahedra and the BaTaO_2_N:Mg surface terminated with the TaN_6_ and TaO_4_N_2_ octahedra showed trend deviations possibly due to simplifications in the MD simulation.

Strain engineering has also been demonstrated to be one of the effective methods in modulating the properties of perovskite oxynitrides. Castelli and co‐workers^[^ ^160^ ^]^ explored the effect of strain engineering on the OER for the different surface terminations of BaTaO_2_N and found that 1% tensile uniaxial strain in the [001] direction could lower the theoretical overpotential from 0.43 V to 0.37 V for the (100) facet TaO_2_N‐terminated surface under (photo)electrochemical conditions (Figure 11b). Zhao et al.^[^ ^161^ ^]^ investigated the effect of tensile and compressive strains on the electronic and optical properties of BaTaO_2_N based on the first‐principles calculations. Their findings indicated that the optical properties of BaTaO_2_N were more sensitive to strains in the^[^ ^100^ ^]^ direction than in the [010] direction, and a pronounced redshift of the absorption edge and the reduction of bandgap energy were observed under tensile strain (Figure 11c). Precise control of the morphology, dimension, particle size, porosity, and surface properties of BaTaO_2_N, as discussed in this section, lays the foundation for further advances in the properties and efficiency of BaTaO_2_N.

## 8 Cation Doping of BaTaO_2_N

Doping is one of the effective strategies to modulate the electronic structure, optical properties, electrical conductivity, charge density, charge mobility, and charge separation and transfer, leading to enhanced water‐splitting efficiency.^[^ ^9^, ^157^ ^]^ Earlier studies have also confirmed that the defect density can be reduced by doping but at the expense of visible light absorption.^[^ ^133^, ^162^ ^]^ The physicochemical and photophysical properties were tuned and the surface local structure and anion ordering were tailored by an intentional introduction of foreign cations with different radii and valences into the *A*‐site or *B*‐site of BaTaO_2_N without altering its perovskite structure.^[^ ^163^ ^]^ Particularly, dopants with different valences act as either electron donors (higher valency) or acceptors (lower valency) and change the carrier concentration.

### 8.1 Monovalent Cation Doping

**Figure 12:**
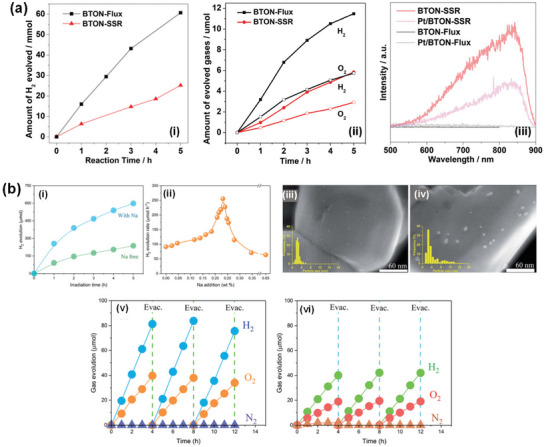
a) Photocatalytic reaction time courses for H_2_ evolution in the presence of methanol (i) and Z‐scheme overall water splitting (ii) of BaTaO_2_N synthesized by flux method and solid‐state reaction and iii) cathodoluminescence (CL) spectra of BTON‐SSR (red), 0.5 wt% Pt‐BTON‐SSR (pink), BTON‐Flux (black), and 0.5 wt% Pt‐BTON‐Flux (grey). Reproduced with permission.^[^ ^118^ ^]^ Copyright 2017, The Royal Society of Chemistry. b) Photocatalytic reaction time courses for H_2_ evolution over 0.28 wt% Pt/BaTaO_2_N with and without 0.23 wt% Na (i). Photocatalytic H_2_ evolution rates over Na‐containing 0.28 wt% Pt/BaTaO_2_N as a function of Na addition (ii). STEM images and particle size distributions of (iii) 0.23 wt% Na‐containing and iv) Na‐free 0.28 wt% Pt/BaTaO_2_N samples after hydrogen reduction at 523 K for 1 h. Effect of Pt loading amount (with constant Na ion level of 0.23 wt%) on photocatalytic reaction time courses for H_2_ and O_2_ evolution over a mixture of v) Na‐containing and vi) Na‐free 0.3 wt% Pt/BaTaO_2_N and H^+^–Cs^+^–0.5 wt% PtO_x_/WO_3_. Reproduced with permission.^[^ ^164^ ^]^ Copyright 2021, The Royal Society of Chemistry.

**Figure 13:**
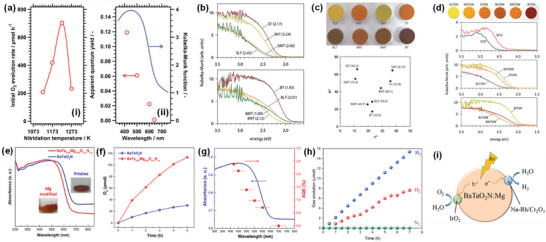
a) Oxygen evolution rate for CoO_x_‐loaded BaTaO_2_N obtained from ammonolysis of (Na_1/4_Ba_3/4_)(Zn_1/4_Ta_3/4_)O_3_ blended with BaCO_3_ and NaCl in aqueous AgNO_3_ solution under visible light irradiation as a function of nitridation temperature (i) and action spectrum for oxygen evolution reaction using CoO_x_‐loaded BaTaO_2_N ammonolyzed at 1223 K along with DRS of oxynitride (ii). Reproduced with permission.^[^ ^165^ ^]^ Copyright 2020, The Royal Society of Chemistry. b) Diffuse‐reflectance absorption spectra of SrTaO_2_N (ST), SrLi_0.2_Ta_0.8_O_2.8_N_0.2_ (SLT), SrNa_0.2_Ta_0.8_O_2.8_N_0.2_ (SNT), SrMg_0.2_Ta_0.8_O_2.6_N_0.4_ (SMT), BaTaO_2_N (BT), BaLi_0.2_Ta_0.8_O_2.8_N_0.2_ (BLT), BaNa_0.2_Ta_0.8_O_2.8_N_0.2_ (BNT), and BaMg_0.2_Ta_0.8_O_2.6_N_0.4_ (BMT). The band gaps, in eV, are shown in parentheses. c) Digital photographs and color coordinates (a*, b*) of SrTaO_2_N (ST), SrLi_0.2_Ta_0.8_O_2.8_N_0.2_ (SLT), SrNa_0.2_Ta_0.8_O_2.8_N_0.2_ (SNT), SrMg_0.2_Ta_0.8_O_2.6_N_0.4_ (SMT), BaTaO_2_N (BT), BaLi_0.2_Ta_0.8_O_2.8_N_0.2_ (BLT), BaNa_0.2_Ta_0.8_O_2.8_N_0.2_ (BNT), and BaMg_0.2_Ta_0.8_O_2.6_N_0.4_ (BMT). Reproduced with permission.^[^ ^166^ ^]^ Copyright 2016, The Royal Society of Chemistry. d) UV−vis spectra for A_5_Ta_4_O_15_, ATaO_2_N, and AM_0.2_Ta_0.8_O_2.8_N_0.2_ (A = Sr, Ba; M = Li, Na) and digital photographs of the sample powders: STO:Sr_5_Ta_4_O_15_, BTO: Ba_5_Ta_4_O_15_, STON: SrTaO_2_N, SLTON: SrLi_0.2_Ta_0.8_O_2.8_N_0.2_, SNTON: SrNa_0.2_Ta_0.8_O_2.8_N_0.2_, BTON: BaTaO_2_N, BLTON: BaLi_0.2_Ta_0.8_O_2.8_N_0.2_, BNTON: BaNa_0.2_Ta_0.8_O_2.8_N_0.2_. Reproduced with permission.^[^ ^167^ ^]^ Copyright 2015, American Chemical Society. e) UV–vis absorption spectra of BaTaO_2_N and BaTa_0.95_Mg_0.05_O_2+x_N_1‐y_, f) Photocatalytic oxygen production of BaTaO_2_N and BaTa_0.95_Mg_0.05_O_2+x_N_1‐y_ under visible light illumination, and g) action spectra of BaTa_0.95_Mg_0.05_O_2+x_N_1‐y_ for O_2_ production. Reproduced with permission.^[^ ^168^ ^]^ Copyright 2020, Elsevier. h) Overall water splitting performance of IrO_2_/Cr_2_O_3_/Na‐Rh/BaTaO_2_N:Mg (Ba/Ta/Mg = 2.5:1:0.1). Reaction time courses of gas evolution over IrO_2_/Cr_2_O_3_/Na‐Rh/BaTaO_2_N:Mg from water under visible light and i) schematic representation of its mechanism. Reproduced with permission.^[^ ^169^ ^]^ Copyright 2022, American Chemical Society.

Despite having ionic radii with sizes close to that of barium in the twelve‐coordinated *A*‐site of perovskite BaTaO_2_N, monovalent alkali‐metal cations are prone to sublimation during high‐temperature synthesis and ammonolysis under an NH_3_ flow. Nevertheless, small amounts of lithium,^[^ ^118^ ^]^ sodium,^[^ ^164^ ^]^ and potassium^[^ ^129^ ^]^ were found in BaTaO_2_N synthesized by the ammonolysis of LiBa_4_Ta_3_O_12_ and a flux method using the KCl and RbCl fluxes, respectively. In the time course of the Z‐scheme overall water splitting reaction over BaTaO_2_N synthesized by the ammonolysis of LiBa_4_Ta_3_O_12_, the evolution rates of H_2_ and O_2_ in the first hour were 3.1 and 1.55 µmol, respectively, confirming the overall water splitting with the stoichiometric H_2_/O_2_ ratio of 2:1 due to the efficient transfer of photo‐excited charge carriers to the surface (**Figure** **12**a).^[^ ^118^ ^]^ A high degree of cocatalyst dispersion and intimate contact with the photocatalyst are important for improving photocatalytic activity. Interestingly, the addition of a small number of Na ions, as a promoter, to the Pt‐loaded BaTaO_2_N enhanced the photocatalytic H_2_ evolution (about 600 µmol within 5 h) under visible light irradiation, resulting in an apparent quantum yield for the H_2_ evolution reaction of ≈1% at 420 nm, and during Z‐scheme overall water splitting, a mixture of Na‐containing Pt/BaTaO_2_N and H^+^–Cs^+^‐modified PtO_x_/WO_3_ evolved stoichiometric amounts of H_2_ and O_2_ from an aqueous NaI solution under visible light up to 12 h (Figure 12b).^[^ ^164^ ^]^ The highest degree of H_2_ evolution activity was obtained for BaTaO_2_N with 0.28 wt% Pt and 0.23 wt% Na. The presence of Na improved the dispersion and structural stability of the Pt cocatalyst, resulting in more efficient electron extraction from BaTaO_2_N, and no Na was incorporated into the BaTaO_2_N lattice. 0.47 at% K was unintentionally introduced from the KCl flux into cube‐like BaTaO_2_N crystals synthesized by an NH_3_‐assisted direct flux method.^[^ ^129^ ^]^ Although cube‐like BaTaO_2_N crystals exhibited the evolution rates of 10.14 µmol H_2_ and 22.18 µmol O_2_ within 6 hours of the photocatalytic half‐reactions, the influence of potassium on the photocatalytic activity of BaTaO_2_N was not investigated. The incorporation of Na^+^ and Zn^2+^ with similar ionic radii to control the crystal structure and composition of the precursor oxide (Na_1/4_Ba_3/4_)(Zn_1/4_Ta_3/4_)O_3_ improved the OER activity of BaTaO_2_N (699 µmol h^−1^), with an apparent quantum yield of 11.9% at 420 nm (**Figure** **13**a).^[^ ^165^ ^]^ However, the exact structures of alkali metal species and their impacts on the local crystal structure, anion ordering, and photocatalytic water splitting activity of BaTaO_2_N were not studied in detail. Moon et al.^[^ ^166^ ^]^ developed a novel synthetic strategy, which accomplishes cation intercalation with concomitant anion substitution, for developing new oxynitrides. The partial replacement of (Ta,N) in BaTaO_2_N by (Li,O) or (Na,O) modified its electronic structure. The compositional variation (BaLi_0.2_Ta_0.8_O_2.8_N_0.2_ and BaNa_0.2_Ta_0.8_O_2.8_N_0.2_) could control the lattice iconicity, optical band gap, and color, which also play an important role in solar water splitting (Figure 13b–d).^[^ ^166^, ^167^ ^]^

### 8.2 Divalent Cation Doping

**Figure 14:**
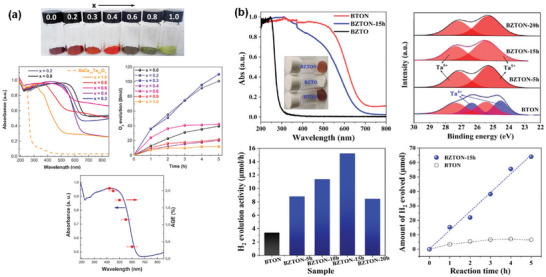
a) Digital photographs, UV‐Vis absorption spectra, visible‐light‐driven photocatalytic oxygen evolution activity of BaCa_x/3_Ta_1‐x/3_O_2+y_N_1‐y_ powders, and action spectra (apparent quantum efficiency (AQE) versus excitation wavelength) of BaCa_0.10_Ta_0.90_O_2.27_N_0.73_ (x = 0.3), 2 wt% CoO_z_ was loaded as a cocatalyst and monochromic light was generated by filtering the output of lamp using bandpass filters. Reproduced with permission.^[^ ^162^ ^]^ Copyright 2018, Elsevier. b) UV–Vis absorption spectra of BTON and BZTON‐15 h, Ta4f XPS spectra of BTON and BZTON samples nitrided for different time, comparison of visible‐light‐driven photocatalytic hydrogen evolution rates, and photocatalytic hydrogen evolution activities of BTON and BZTON‐15 h as a function of reaction time. Reproduced with permission.^[^ ^171^ ^]^ Copyright 2021, Elsevier.

The effect of divalent cation doping on the photocatalytic activity of BaTaO_2_N was also explored. Particularly, Mg^2+^ doping was found to be beneficial to shorten the Ta–O/N bonds or to strengthen the covalence bonding networks between Ta and O/N, which is favorable for inhibiting the formation of Ta^4+^‐related defects and improving the charge separation (Figure 13e–g).^[^ ^168^ ^]^ This is because hybridizations between Ta 5d and O/N 2p orbitals have major contributions to the conduction and valence bands of BaTaO_2_N near the Fermi level. As a result, an apparent quantum efficiency of 2.59% at 420 ± 20 nm was achieved for Mg^2+^‐doped BaTaO_2_N. Also, a 5% Mg^2+^ doping altered the electronic, optical, and surface properties and significantly enhanced the photocatalytic O_2_ evolution reaction rate (503.6 µmol) of BaTaO_2_N crystals grown by a flux method.^[^ ^133^ ^]^ The Mg doping along with Cr_2_O_3_/(Na)Rh and IrO_2_ co‐catalyst loading led to the one‐step excitation overall water splitting with an apparent quantum yield of 0.08% at 420 nm and an STH conversion efficiency of 4 × 10^−4^% for BaTaO_2_N synthesized by an RbCl flux‐assisted ammonolysis (Figure 13h,i).^[^ ^169^ ^]^ Mg was found to promote a charge transfer to the loaded co‐catalysts. Moon et al.^[^ ^166^ ^]^ found that among various magnesium sources, such as oxide, halides, hydroxide, and carbonate, MgCl_2_ was the most suitable one to successfully intercalate Mg^2+^ into the layered oxide precursor Ba_5_Ta_4_O_15_, which was then ammonolyzed at 930 °C for 48 h to obtain BaMg_0.2_Ta_0.8_O_2.6_N_0.4_. By incorporating and increasing the amount of Ca^2+^ in the BaTaO_2_N lattice, Xu and co‐workers^[^ ^162^ ^]^ observed the enlargement of optical bandgap energy and the decrease of defect density and nitrogen content. The formation of defects, such as Ta^4+^ species, was effectively suppressed, the photocatalytic activity for water oxidation was significantly enhanced, and the stability against photocatalytic self‐decomposition was largely improved upon partial substitution of Ca^2+^ in BaTaO_2_N due to the efficient charge separation and longer lifetime of photo‐excited electrons (**Figure** **14**a). Nearly 3‐fold enhancement in oxygen evolution was reached for BaCa_0.10_Ta_0.90_O_2.27_N_0.73_ in comparison to pristine BaTaO_2_N, and an apparent quantum efficiency of ≈2.1% at 420 ± 20 nm was achieved for Ca^2+^‐doped BaTaO_2_N. Castelli and co‐workers^[^ ^160^ ^]^ theoretically investigated the effect of divalent cation (Ca^2+^ or Sr^2+^) doping and strain engineering on the OER for differently terminated surfaces of BaTaO_2_N. The smallest theoretical overpotential (0.53 V) was obtained for Ca^2+^‐doped TaON‐terminated BaTaO_2_N (001) clean surface, with 4% tensile uniaxial strain in the [010] direction (Figure 11b). A simple crossover from the two‐dimensional (2D) to the three‐dimensional (3D) correlated disorder of O and N atoms on a cubic lattice was discovered within Ba_1–_ *_x_* Sr*_x_* TaO_2_N (0 ≤*x* ≤1) by Johnston et al.^[^ ^170^ ^]^ The Ba_1–_ *_x_* Sr*_x_* TaO_2_N with cubic *Pm* $\bar{3}$ *m* structure at *x* = 0–0.2 is consistent with a 3D distribution of disordered *cis*‐chains, while neutron occupancies reveal that a long‐range 2D confinement of anion chain layers is present across the tetragonal *P*4/*mmm* symmetry at *x* = 0.4–1. The local structures of ATaO_2_N (A = Ba, Sr, and Ca) were investigated by Page and co‐workers^[^ ^86^ ^]^ using a combination of experimental and theoretical approaches, including neutron total scattering, density functional theory (DFT), and ab initio molecular dynamics (AIMD) simulations. Their study shows that the local *cis* ordering and Ta off‐centering can play decreasing roles in overall lattice stability, overshadowed by the stabilizing effects of octahedral tilting, by replacing Ba^2+^ with Sr^2+^ or Ca^2+^ in BaTaO_2_N. The findings indicate that the anion order may have a larger influence on local dipoles and dipole ordering in such perovskite systems. Zn^2+^ with an effective ionic radius of 0.68 Å, which is close to that of Ta (0.64 Å), was substituted in the crystal lattice site of Ta in BaTaO_2_N by the synthesis and ammonolysis of the Ba(Zn_1/3_Ta_2/3_)O_3_ precursor.^[^ ^171^ ^]^ The reduction and sublimation of Zn^2+^ during the ammonolysis process and the effective charge balance by Zn^2+^ resulted in an extremely low concentration of Ta^4+^‐associated defects, efficient charge separation, and improved photocatalytic HER rate of about 65 µmol within 5 h (Figure 14b).

### 8.3 Trivalent Cation Doping

Trivalent cation (Al^3+^, Ga^3+^, and Sc^3+^) doping was found to be useful for shifting the respective conduction band potential (*E*_CB_) of BaTaO_2_N to more negative values.^[^ ^133^ ^]^ The photocatalytic HER rate increased significantly from 6.59 µmol H_2_ h^−1^ (pristine BaTaO_2_N) to 21.92, 10.27, and 12.12 µmol H_2_ h^−1^ by doping 5% Al^3+^, Ga^3+^, and Sc^3+^, respectively, revealing the advantageous effect of trivalent dopants. In contrast, the photocatalytic OER rate decreased from 316.3 µmol (pristine BaTaO_2_N) to 252.5 and 188.0 µmol when 5% Ga^3+^ and Al^3+^ were substituted in BaTaO_2_N, whereas a 5% Sc^3+^ dopant increased the photocatalytic OER rate within 5 h. Interestingly, 1% La^3+^ doping resulted in the HER rate that is three times lower than that of pristine BaTaO_2_N.^[^ ^172^ ^]^

Based on synchrotron X‐ray powder diffraction analyses, Kim and Woodward^[^ ^173^ ^]^ found that BaSc_0.05_Ta_0.95_O_2.1_N_0.9_ has a simple cubic symmetry similar to BaTaO_2_N, whereas LaMg_1/3_Ta_2/3_O_2_N and LaMg_1/2_Ta_1/2_O_5/2_N_1/2_ are isostructural to the oxide La_2_Mg(Mg_1/3_Ta_2/3_)O_6_ (space group *P*21/*n*). The impedance spectroscopy analysis revealed an interesting capacitor geometry in BaSc_0.05_Ta_0.95_O_2.1_N_0.9_ in which the semiconducting oxynitride grains are separated by the insulating secondary phases. Although the Rietveld refinement implies the presence of secondary phases like BaSc_2_O_4_, Sc_2_O_3_, or ScN, no impurity phases were detected in the synchrotron XRPD measurements. The diffusion of Sc‐rich phase out of bulk to form amorphous shells surrounding crystalline BaTaO_2_N grains was proposed as a plausible model for such behavior.

**Figure 15:**
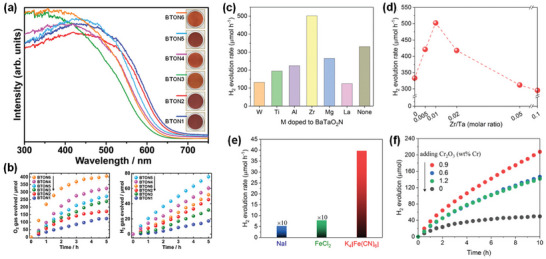
a) UV‐Vis diffuse reflectance spectra and b) reaction time courses for photocatalytic O_2_ and H_2_ evolution over BaTaO_2_N powders with no substituent (BTON1), 5% Al^3+^ (BTON2), 5% Mg^2+^ (BTON3), 2.5% Al^3+^ + 2.5% Mg^2+^ (BTON4), 3.5% Al^3+^ + 1.5% Mg^2+^ (BTON5), and 1.5% Al^3+^ + 3.5% Mg^2+^ (BTON6) loaded with CoO_x_ and Pt nanoparticles under visible light irradiation. Reproduced with permission.^[^ ^174^ ^]^ Copyright 2022, The Royal Society of Chemistry. Photocatalytic H_2_ evolution rates of Na–Pt/BaTaO_2_N doped with various metal cations with an M/Ta ratio of 0.01 (c) and Na–Pt/BaTaO_2_N:Zr with different Zr/Ta molar ratios (d) in an aqueous solution containing methanol (15 vol%). e) Photocatalytic H_2_ evolution activities of Cr_2_O_3_/Na‐Pt/BaTaO_2_N:Zr0.01 (100 mg) in aqueous solutions of NaI, FeCl_2_, and K_4_[Fe(CN)_6_] (150 mL, 2 mM each) with pH values of 6–7, 2.3, and 6, respectively. f) Reaction time courses of H_2_ evolution over Cr_2_O_3_/0.23 wt% Na 0.3 wt% Pt/BaTaO_2_N:Zr0.01 with varying amounts of Cr in an aqueous 50 mM sodium phosphate buffer solution at pH 6 (150 mL) containing 6 mM K_4_[Fe(CN)_6_]. Reproduced with permission.^[^ ^172^ ^]^ Copyright 2023, The Royal Society of Chemistry.

Partial Al^3+^–Mg^2+^ dual substitution (5%) was applied to engineer structural defects and to modulate optoelectronic and surface properties and photocatalytic activity of BaTaO_2_N.^[^ ^174^ ^]^ The optical absorption edge of BaTaO_2_N shifted to shorter wavelengths after (co)substitution of Al^3+^ and/or Mg^2+^ for Ta^5+^, leading to the increase in the optical bandgap energy. This effect was more pronounced in the BaTaO_2_N samples with higher levels of Mg^2+^ dopant because a large number of O^2−^ were substituted for N^3−^ to compensate charge balance. The initial photocatalytic reaction rates for O_2_ and H_2_ evolution confirmed the enhancement of the photocatalytic performance of BaTaO_2_N due to the dual substitution of Al^3+^ and Mg^2+^ for Ta^5+^ (**Figure** **15**a). Especially, BaTaO_2_N modified with 1.48% Al^3+^ + 3.51% Mg^2+^ generated the highest quantity of O_2_ (178.66 µmol h^−1^) with an apparent quantum efficiency of 0.18% at 420 nm, while BaTaO_2_N modified with 3.47% Al^3+^ + 1.52% Mg^2+^ produced the highest quantity of H_2_ (18.94 µmol h^−1^) with an apparent quantum efficiency of 0.64% at 420 nm. The enhancement achieved by partial Al^3+^–Mg^2+^ dual substitution is related to the changes in the defect density, dynamics of charge carriers, electronic band structure, improvement in water and methanol adsorption, and a favorable shift in the band energy levels with respect to water reduction and oxidation potentials.

### 8.4 Tetravalent Cation Doping

Tetravalent cation (Zr^4+^ and Ti^4+^) doping lowered the photoelectrochemical performance of BaTaO_2_N photoanodes due to the decreased donor density and reduced titanium species (Ti^3+^), which generated anion defects simultaneously, increasing the donor density but facilitated the recombination rate between photo‐excited electrons and holes through the redox cycle.^[^ ^175^ ^]^ The stabilization of the +4 oxidation state of titanium can be achieved by the inductive effect of doped rare‐earth elements that share some electrons with the closest *TM*–O,N bond to enhance its covalency.^[^ ^176^ ^]^ In contrast, 5% Zr‐doped BaTaO_2_N showed a high photocatalytic activity in both O_2_ (446.8 µmol) and H_2_ (80.4 µmol) evolution half‐reactions within 5 h due to the altered potentials of the valence and conduction bands by Zr^4+^ doping.^[^ ^133^ ^]^ Introducing Zr^4+^ did not change the *E*_CB_ value compared to that of pristine BaTaO_2_N presumably due to the compensation given by the change of valence band (*E*_VB_) to more positive values. This is possible if it is considered that the incorporation of a dopant with a larger ionic radius (Zr^4+^) leads to a greater substitution of O^2−^ for N^3−^ to compensate ionic charge imbalance, justifying the change in the *E*_VB_ in the Zr‐doped BaTaO_2_N. Recently, Li et al.^[^ ^172^ ^]^ found that the incorporation of 1% Zr in BaTaO_2_N could enhance the photocatalytic H_2_ evolution activity to the greatest extent (≈500 µmol h^−1^) because of the extension of the lifetime of electrons and the promotion of electron injection into the Na‐Pt cocatalyst, while the higher doping levels reduced the activity. The Z‐scheme overall water splitting (ZOWS) system based on Cr_2_O_3_/Na–Pt/BaTaO_2_N:Zr0.01 as the HEP, CoO_x_/Au/BiVO_4_ as the OEP, and [Fe(CN)_6_]^3−/4−^ as redox ions evolved H_2_ and O_2_ under visible light up to 520 nm, and its STH conversion efficiency reached 0.022% and the apparent quantum yields at 420 nm and 520 nm under monochromatic light were 1.5% and 0.2%, respectively (Figure 15b).

### 8.5 Pentavalent Cation Doping

**Figure 16:**
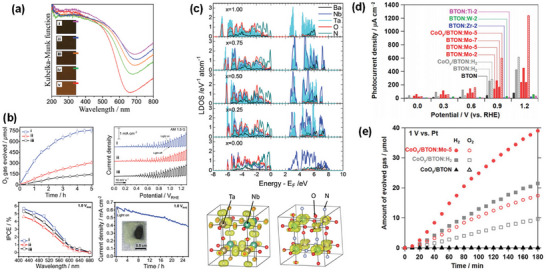
a) UV‐Vis diffuse reflectance spectra of i) BaNbO_2_N, ii) BaNb_0.75_Ta_0.25_O_2_N, iii) BaNb_0.50_Ta_0.50_O_2_N, iv) BaNb_0.25_Ta_0.75_O_2_N, and v) BaTaO_2_N crystals. b) Reaction time courses of photocatalytic O_2_ evolution, *I*–*E* curves, and wavelength dependence of IPCE of BaTaO_2_N crystals after flux growth (i), oxidation at 300 °C for 1 h and ammonolysis at 900 °C for 5 h (ii), and ammonolysis at 900 °C for 5 h (iii). Current density versus time of BaTaO_2_N crystals (iv). c) Local density of states (LDOS) of i) BaNbO_2_N, ii) BaNb_0.75_Ta_0.25_O_2_N, iii) BaNb_0.50_Ta_0.50_O_2_N, iv) BaNb_0.25_Ta_0.75_O_2_N, and v) BaTaO_2_N and partial electron density distribution around the valence band maximum and the conduction band minimum of BaNb_0.5_Ta_0.5_O_2_N. Reproduced with permission.^[^ ^177^ ^]^ Copyright 2016, The Royal Society of Chemistry. d) Influence of the cation‐doping on the oxidative photocurrent densities generated by the BaTaO_2_N/Ti and CoO_y_/BaTaO_2_N/Ti electrodes in an aqueous Na_2_SO_4_ solution (pH 6) under visible light irradiation. e) Reaction time courses of H_2_ and O_2_ evolution in a two‐electrode system composed of CoO_y_/BTON:Mo‐5/Ti, CoO_y_/BTON:H_2_/Ti, or CoO_y_/BTON/Ti electrode and Pt‐wire coated with Cr_2_O_3_ in phosphate buffer solution (pH 8) under visible light irradiation. Reproduced with permission.^[^ ^175^ ^]^ Copyright 2015, AIP Publishing LLC.

Although BaNbO_2_N can absorb visible light up to 740 nm, a partial substitution of pentavalent niobium for Ta^5+^ in BaTaO_2_N reduced the photocatalytic O_2_ evolution reaction rate from 331.1 µmol to 46.8 µmol in the first 1 h of the photocatalytic reaction despite an insignificant change in the local density of states (**Figure** **16**a,c).^[^ ^177^ ^]^ This is attributed to the presence of reduced niobium species and anion defects because Nb^5+^ is reduced more easily under a reducing NH_3_ atmosphere than Ta^5+^ because of the higher electronegativity of the former.^[^ ^178^ ^]^

### 8.6 Hexavalent Cation Doping

Hexavalent Mo and W species with the [Kr] 4d^5^5s^1^ and [Xe] 4f^14^5d^4^6s^2^ electronic configurations were partially substituted for Ta^5+^ to control the donor density in the bulk for improving the performance of photoelectrochemical water splitting on porous BaTaO_2_N photoanodes under visible light irradiation (Figure 16d,e).^[^ ^175^ ^]^ The Faradic efficiencies for H_2_ and O_2_ evolution were confirmed to be about 93% in the reaction. The partial substitution of Ta^5+^ in BaTaO_2_N by Mo^6+^ (up to 5%) was found to increase the donor density effectively, enhancing the photoelectrochemical performance. In contrast, the decreased photoelectrochemical performance in W‐doped BaTaO_2_N was noted due to the facilitated recombination rate through the redox cycle between W^4+^ and W^6+^ species.

In general, the water‐splitting performance may be decreased when transition metal cations with partly filled orbitals are doped in semiconductor‐based photocatalysts. The doped elements can form either a donor or acceptor level in the forbidden band of semiconductors. Despite the improved visible‐light absorption, dopants can also hinder a fast transfer of photo‐excited charge carriers at the surface and in the bulk. Therefore, it is necessary to gain deeper insights into the effect of dopants on the local crystal structure, anion ordering, electronic structure, optical properties, electrical conductivity, charge density, charge mobility, charge separation and transfer, and solar water splitting performance of perovskite BaTaO_2_N.

## 9 Solid Solutions with BaTaO_2_N

**Figure 17:**
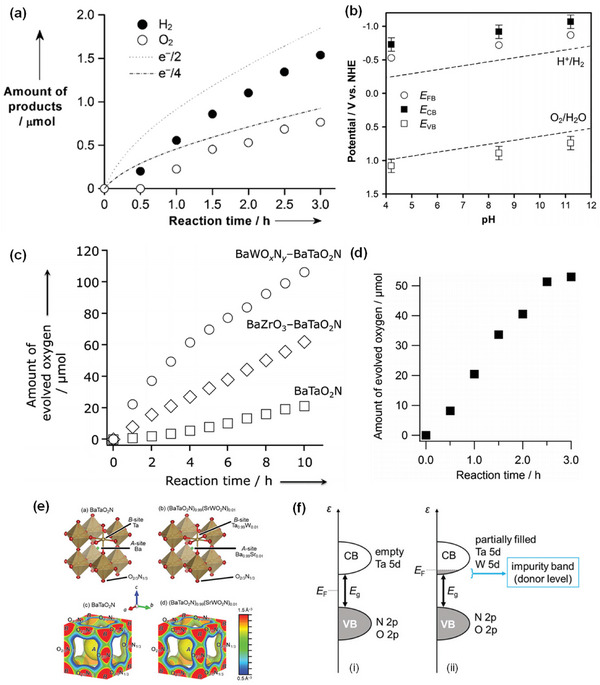
a) Reaction time courses of H_2_ and O_2_ evolution in photoelectrochemical water splitting at +1.0 V versus a Pt wire cathode under simulated sunlight in 0.1 m aqueous Na_2_SO_4_ solution (pH 5.9) using IrO_2_‐loaded TiO_2_/BaZrO_3_‐BaTaO_2_N electrode. Reproduced with permission.^[^ ^180^ ^]^ Copyright 2012, Wiley‐VCH Verlag GmbH & Co. KGaA. b) Dependence of conduction and valence band edge potentials for BaZrO_3_–BaTaO_2_N (Zr/Ta = 0.025) on pH of electrolyte. Reproduced with permission.^[^ ^115^ ^]^ Copyright 2014, Elsevier. c) Reaction time courses of O_2_ evolution on 1.5 wt% IrO_2_‐loaded BaTaO_2_N and BaWO_x_N_y_‐BaTaO_2_N (W/Ta = 0.005) under visible‐light irradiation. Reproduced with permission.^[^ ^181^ ^]^ Copyright 2013, Wiley‐VCH Verlag GmbH & Co. KGaA. d) Reaction time course of O_2_ evolution on 0.5 wt% IrO_2_ ‐loaded (BaTaO_2_N)_0.99_(SrWO_2_N)_0.01_ under visible‐light irradiation, e) refined crystal structures with thermal ellipsoids and MEM electron‐density distribution on the (100) planes with yellow equi‐density surface at 0.5 Å^−3^ of (BaTaO_2_N)_1−x_(SrWO_2_N)_x_ and f) schematic density of states for i) intrinsic semiconductor BaTaO_2_N and ii) *n*‐type semiconductor (BaTaO_2_N)_1−x_(SrWO_2_N)_x_. Reproduced with permission.^[^ ^182^ ^]^ Copyright 2017, The Royal Society of Chemistry.

**Figure 18:**
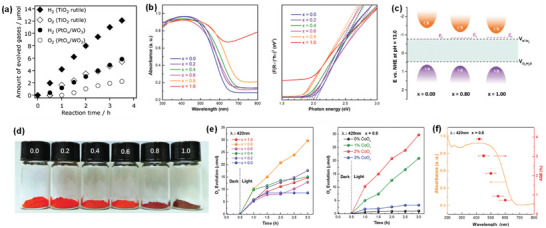
a) Reaction time courses of H_2_ and O_2_ evolution over a mixture of 0.3 wt% Pt/BaZrO_3_−BaTaO_2_N and PtO_x_/WO_3_. Reproduced with permission.^[^ ^184^ ^]^ Copyright 2013, American Chemical Society. b) UV–Vis absorption spectra and Tauc plots of La_1−x_Ba_x_TaO_1+y_N_2−y_ (0 ≤ x,y ≤ 1), c) schematic representation of band edge positions of LaTaON_2_ (*x* = 0.0), La_0.2_Ba_0.8_TaO_1+y_N_2−y_ (*x* = 0.8) and BaTaO_2_N (*x* = 1.0), d) digital photographs of La_1−x_Ba_x_TaO_1+y_N_2−y_ (0 ≤ x,y ≤ 1) powders, and e) reaction time courses of photocatalytic O_2_ evolution of La_1−x_Ba_x_TaO_1+y_N_2−y_ (0 ≤ x,y ≤ 1) and La_0.2_Ba_0.8_TaO_1+y_N_2−y_ (*x* = 0.8) loaded with different amounts of CoO_x_, and f) action spectrum of 2 wt% CoO*_x_*‐loaded La_0.2_Ba_0.8_TaO_1+y_N_2−y_ (*x* = 0.8) for O_2_ evolution. Reproduced with permission.^[^ ^185^ ^]^ Copyright 2021, The Royal Society of Chemistry.

Creating a solid solution with a wide‐band gap oxide semiconductor is beneficial for enhancing the photocatalytic activity of various (oxy)nitrides.^[^ ^179^ ^]^ The BaZrO_3_–BaTaO_2_N (0 ≤ Zr/Ta ≤ 0.1) solid solutions with band gaps of 1.7–1.8 eV showed increasing rates of photocatalytic HER and OER under visible light irradiation above 660 nm with increasing the Zr/Ta ratio and the IPCE of the IrO_2_/TiO_2_/BaZrO_3_‐BaTaO_2_N/FTO electrode for water oxidation was estimated to be ≈1.0% at 1.2 V versus RHE under 500 nm monochromatic light (**Figure** **17**a).^[^ ^180^ ^]^ This was the first report of a photocatalytic material that is capable of both reducing and oxidizing water even under irradiation above 660 nm. This is because the conduction band minimum and the valence band maximum of BaZrO_3_–BaTaO_2_N solid solution straddle the water‐splitting potential. The apparent quantum yields of H_2_ and O_2_ evolution were about 0.06% and 0.03% at 420 nm, respectively. A relatively low apparent quantum yield for water oxidation was obtained owing to a small energy difference (≈0.3 eV) between the water oxidation potential and the valence band maximum.^[^ ^115^ ^]^ The dependence of the conduction and valence band edge potentials of the IrO_2_/TiO_2_/BaZrO_3_–BaTaO_2_N/FTO electrode was also studied as a function of pH. It was found that the positions of the conduction band minimum and valence band maximum of BaZrO_3_–BaTaO_2_N were dependent on pH, which were satisfactory for water oxidation at all pH values against H_2_ evolution at the counter electrode (Figure 17b).^[^ ^115^ ^]^ The BaWO_x_N_y_‐BaTaO_2_N (0 < W/Ta < 0.05)^[^ ^181^ ^]^ and (BaTaO_2_N)_1–_ *_x_* (SrWO_2_N)*_x_* (*x* = 0.01)^[^ ^182^ ^]^ exhibited photocatalytic OER rates that were much higher than those of pristine BaTaO_2_N, SrWO_2_N, and BaZrO_3_‐BaTaO_2_N. In the BaWO*_x_* N*_y_*–BaTaO_2_N and (BaTaO_2_N)_1–_ *_x_* (SrWO_2_N)*_x_*, the introduced W^5+^ species formed a donor level just below the conduction band, promoting the *n*‐type semiconducting nature of BaTaO_2_N (Figure 17c–f). This led to the upward band bending and increased the density of *d* electrons originating from pentavalent W species. The photo‐excited holes in the valence band could easily migrate to the surface according to the upward band bending, enhancing the water oxidation activity. Because of an increased driving force for surface redox reactions and fewer defects in comparison to BaTaO_2_N, the BaZrO_3_‐BaTaO_2_N solid solution exhibited a six‐ to nine‐fold improvement in photocatalytic non‐sacrificial H_2_ evolution from water under visible light irradiation, and an apparent quantum yield for the reaction was calculated to be about 0.6% at 420–440 nm.^[^ ^183^ ^]^ Despite its small bandgap energy to drive water reduction and oxidation, the BaZrO_3_−BaTaO_2_N solid solution modified with Pt nanoparticles as water reduction promoters in combination with either PtO_x_/WO_3_ or rutile‐TiO_2_ as an O_2_ evolution photocatalyst in the presence of an IO_3_^−^/I^−^ shuttle redox mediator exhibited a solar‐driven Z‐scheme water splitting (**Figure** **18**a).^[^ ^184^ ^]^ On the other hand, it was difficult to achieve simultaneous H_2_ and O_2_ evolution in the presence of Fe^3+^/Fe^2+^ redox couple due to the competitive oxidation of Fe^2+^.

Low photocatalytic activity is often associated with high defect density, structural distortions, and unsuitable band edge alignments that hamper the transfer of photo‐excited charge carriers. In perovskite ATaO_2_N, structural characteristics (e.g., Ta–O/N distance, Ta–O/N–Ta angle, etc.) and optoelectronic properties (e.g., band structure, band gap, etc.) can be tuned by controlling the solid solution levels, which govern the photocatalytic performance. The La_1‐x_Ba_x_TaO_1+y_N_2–y_ (0 ≤ x,y ≤ 1) solid solutions were synthesized by a high‐temperature ammonolysis of oxide precursors obtained by a polymerized complex (PC) method.^[^ ^185^ ^]^ The solid solutions exhibited higher photocatalytic activity for water oxidation than their parent compounds. Among them, the La_0.2_Ba_0.8_TaO_1+y_N_2‐y_ (*x* = 0.8) solid solution showed the highest photocatalytic activity for water oxidation with an apparent quantum efficiency of 3.91% at 420 ± 20 nm. This is because its conduction band minimum is positioned positively than that of LaTaON_2_ and its valence band maximum is located negatively than that of BaTaO_2_N, resulting in a suitable overpotential for water oxidation and reduction (Figure 18b–f).

Various compounds containing transition‐metal cations with *d* ^0^ electron configuration (e.g., Nb^5+^) were explored to be active for solar water splitting. However, the lifetimes of the photo‐excited holes become shorter once electrons from the conduction band or mid‐gap states are available for recombination.^[^ ^186^ ^]^ As mentioned earlier, Nb^5+^ is reduced more easily than Ta^5+^ under a high‐temperature NH_3_ atmosphere, and the photo‐excited charge carriers in Nb‐based oxynitrides may not have sufficient reactivity for solar water splitting in comparison to Ta‐based counterparts.^[^ ^178^ ^]^ Thus, in the BaNb_1−_ *_x_* Ta*_x_* O_2_N (*x* = 0.25, 0.50, 0.75) solid solutions, the increased concentration of Ta^5+^ substituted for Nb^5+^ led to the reduction of crystal size and background absorption intensity and the monotonic increase in the photocatalytic OER rate due to a more positively positioned valence band maximum and a lowered density of mid‐gap states associated with defects.^[^ ^177^ ^]^

**Figure 19:**
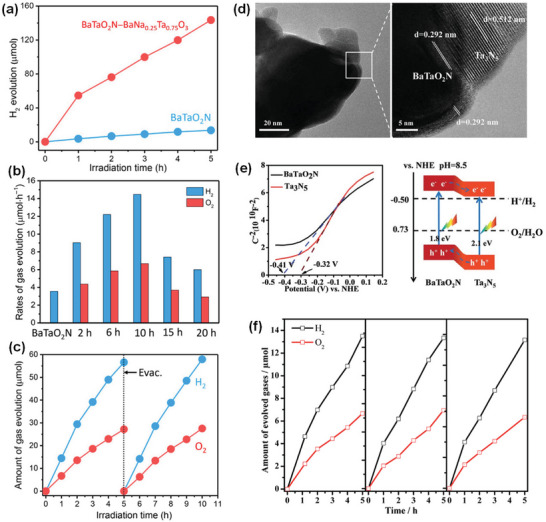
a) Reaction time courses of H_2_ evolution of BaTaO_2_N and BaTaO_2_N–BaNa_0.25_Ta_0.75_O_3_, b) H_2_ and O_2_ evolution rates during Z‐scheme overall water splitting reaction over BaTaO_2_N and BaTaO_2_N–BaNa_0.25_Ta_0.75_O_3_ synthesized by varying ammonolysis times, and c) reaction time courses of H_2_ and O_2_ evolution during Z‐scheme overall water splitting over BaTaO_2_N–BaNa_0.25_Ta_0.75_O_3_ synthesized by ammonolysis for 10 h for the stability test. Reproduced with permission.^[^ ^187^ ^]^ Copyright 2022, American Association for the Advancement of Science. d) TEM and HRTEM images of Ba(0.3)–Ta_3_N_5_ sample, e) Mott–Schottky plots and schematic representation of band structures of Ta_3_N_5_ and BaTaO_2_N, and f) multiple cycles of Z‐scheme overall water splitting with 0.5 wt% Pt/Ba(0.3)–Ta_3_N_5_ and 0.45 wt% PtO_x_/WO_3_. Reproduced with permission.^[^ ^192^ ^]^ Copyright 2017, The Royal Society of Chemistry.

The BaTaO_2_N–BaNa_0.25_Ta_0.75_O_3_ solid solution was synthesized by the ammonolysis of BaNa_0.25_Ta_0.75_O_3_ oxide precursor having a lattice‐matched crystal structure with BaTaO_2_N and containing volatile Na.^[^ ^187^ ^]^ During high‐temperature ammonolysis, the lattice matching decreased the thermodynamic barrier of atomic diffusion rearrangement, and the sublimation of Na facilitated the direct phase transition of BaNa_0.25_Ta_0.75_O_3_ to BaTaO_2_N. These processes significantly inhibited the formation of defects. Therefore, the BaTaO_2_N‐BaNa_0.25_Ta_0.75_O_3_ solid solution exhibited higher photocatalytic activity (54.62 µmol h^−1^) for H_2_ evolution than pristine BaTaO_2_N (3.56 µmol h^−1^; **Figure** **19**a–c). In Z‐scheme overall water splitting, Pt‐modified BaTaO_2_N–BaNa_0.25_Ta_0.75_O_3_ as the H_2_ evolution photocatalyst and surface‐treated WO_3_ as the O_2_ evolution photocatalyst, and IO_3_^−^/I^−^ as the electron mediator exhibited the highest photocatalytic activity, giving the H_2_ and O_2_ evolution rates of 14.46 and 6.68 µmol h^−1^, respectively. No solid solutions between CaTaO_2_N and BaTaO_2_N were formed because of a large disparity in cation sizes, while complete solid solutions between SrTaO_2_N and BaTaO_2_N were obtained, changing the cubic $Pm\bar{3}m$ BaTaO_2_N to the tetragonal *I*4/*mcm* SrTaO_2_N at about *x* = 0.5–0.6 in Ba*_x_* Sr_1‐_ *_x_* TaO_2_N.^[^ ^188^ ^]^ This is consistent with the earlier study on Ba*_x_* Sr_1‐_ *_x_* TaO_2_N, conducted by Pors et al.^[^ ^189^ ^]^ The *A*Zr*_x_* Ta_1‐_ *_x_* O_2+_ *_x_* N_1‐_ *_x_* (*A* = Ca, Sr, Ba) solid solutions were synthesized using Zr–Ta xerogels and ACO_3_.^[^ ^190^ ^]^ The unit cell parameters increased with *x* as the Ta^5+^ ions were substituted by larger Zr^4+^ ions. The CaZr*_x_* Ta_1‐_ *_x_* O_2+_ *_x_* N_1‐_ *_x_* and BaZr*_x_* Ta_1‐_ *_x_* O_2+_ *_x_* N_1‐_ *_x_* solid solutions were found to be orthorhombic and cubic for all *x* values, respectively, whereas the SrZr*_x_* Ta_1‐_ *_x_* O_2+_ *_x_* N_1‐_ *_x_* solid solutions were orthorhombic for *x* ≥ 0.60 and cubic for *x* ≤ 0.60. Both studies mainly emphasized the synthesis and structural analysis of solid solutions, and the photocatalytic performance of these solid solutions for solar water splitting was not investigated.

## 10 Heterostructures, Heterojunctions, and Photoelectrochemical Cells with BaTaO_2_N

The separation efficiency of photo‐excited charge carriers can also be improved by forming the heterostructures without losing their light absorption ability. The following criteria must be met in order to develop the efficient heterostructures: i) the matching band‐edge positions that thermodynamically allow a photocarrier transfer across their interfaces, ii) the maximized contact that allows multiple charge transfer pathways, and iii) the reduced structure mismatch across the interfaces that avoid nonstoichiometry and dangling bonds which may become trapping sites for photo‐excited charge carriers.^[^ ^191^ ^]^

**Figure 20:**
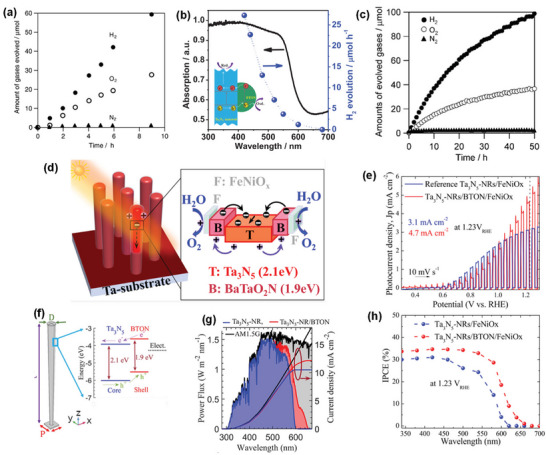
a) Reaction time courses of H_2_ and O_2_ evolution over a mixture of 0.3 wt% Pt–BaTaO_2_N and 0.5 wt% Pt–WO_3_ under visible light. Reproduced with permission.^[^ ^107^ ^]^ Copyright 2008, Elsevier. b) Cutoff wavelength dependence performance (blue circles) and UV–vis diffuse reflectance spectrum (black line) of the Ta_3_N_5_/BaTaO_2_N. Reproduced with permission.^[^ ^193^ ^]^ Copyright 2019, Wiley‐VCH Verlag GmbH & Co. KGaA. c) Reaction time courses of H_2_ and O_2_ evolution over a mixture of Pt‐BaTaO_2_N and Pt‐WO_3_ under visible light irradiation. Reproduced with permission.^[^ ^194^ ^]^ Copyright 2009, American Chemical Society. d) Schematic representation of charge separation and transfer and water oxidation of Ta_3_N_5_‐NRs/BaTaO_2_N/FeNiO_x_, e) photocurrent density versus voltage curves of Ta_3_N_5_‐NRs/FeNiO_x_ and Ta_3_N_5_‐NRs/BaTaO_2_N/FeNiO_x_ photoanodes, f) conceptual model and corresponding energy band alignment of Ta_3_N_5_‐NR and core−shell Ta_3_N_5_‐NR/BaTaO_2_N photoanodes, g) solar light absorption along with the integrated current density, and h) IPCE spectra of Ta_3_N_5_‐NRs/FeNiO_x_ and Ta_3_N_5_‐NRs/BaTaO_2_N/FeNiO_x_ photoanodes. Reproduced with permission.^[^ ^195^ ^]^ Copyright 2020, American Chemical Society.

The construction of a Ta_3_N_5_/BaTaO_2_N heterostructure by a one‐pot synthesis method decreased the defect density, improved the spatial charge separation efficiency, and enhanced the relatively poor photocatalytic proton reduction activity of Ta_3_N_5_ due to the formation of BaTaO_2_N on the surface of Ta_3_N_5_ leading to surface passivation.^[^ ^192^ ^]^ A visible‐light‐driven Z‐scheme overall water splitting system constructed by using Ta_3_N_5_/BaTaO_2_N heterostructure, PtO_x_/WO_3_, and IO_3_^−^/I^−^ pair as H_2_‐ and O_2_‐evolving photocatalysts and redox mediator, respectively, showed an apparent quantum efficiency of 0.1% at 420 nm (Figure 19d–f). Earlier, Domen and co‐workers^[^ ^107^ ^]^ first achieved overall water splitting under visible light beyond 600 nm for the combination of Pt–BaTaO_2_N as the H_2_‐evolving photocatalyst and Pt–WO_3_ as the O_2_‐evolving photocatalyst in the presence of IO_3_^−^/I^−^ as a shuttle redox‐mediator (**Figure** **20**a). This demonstrated the potential of a two‐step water‐splitting system in a broader region of the visible spectrum. The Z‐scheme overall water splitting was successfully achieved under visible light irradiation using barium‐modified Ta_3_N_5_, PtO_x_/WO_3_, and IO_3_^−^/I^−^as the H_2_‐ and O_2_‐evolving photocatalysts and a redox mediator, respectively. In the Ta_3_N_5_/BaTaO_2_N heterostructure composed of Ta_3_N_5_ nanorods (1D) and BaTaO_2_N nanoparticles (0D), the Ta_3_N_5_ nanorods could transfer electrons along the rod orientation direction, while the holes moved in the lateral direction. The intimate interface of the formed heterostructure was thought to stem from similar Ta‐containing octahedron units of Ta_3_N_5_ and BaTaO_2_N, which promoted a charge separation and enhanced the solar hydrogen production from water splitting one order of magnitude (Figure 20b).^[^ ^193^ ^]^ The BaMg_1/3_Ta_2/3_O_3‐x_N_y_/Ta_3_N_5_ heterostructure synthesized by a one‐pot ammonolysis strategy exhibited superior charge separation and transfer ability and 20 times higher photocatalytic activity for proton reduction with respect to the corresponding counterparts, and the apparent quantum efficiency of Pt‐loaded BaMg_1/3_Ta_2/3_O_3‐x_N_y_/Ta_3_N_5_ (0.4) was measured to be 0.1% at 420 nm as the H_2_‐evolving photocatalyst.^[^ ^194^ ^]^ Higashi et al.^[^ ^195^ ^]^ achieved overall water splitting into H_2_ and O_2_ under visible light with an apparent quantum efficiency of ≈0.1% at 420–440 nm by combining Pt–BaTaO_2_N and Pt–WO_3_ in the presence of ${\mathrm{IO}}_{3}^{-}$/I*^−^* as a shuttle redox mediator with (Figure 20c).

Pihosh et al.^[^ ^196^ ^]^ fabricated a core‐shell heterojunction photoanode of Ta_3_N_5_‐nanorods/BaTaO_2_N by combining glancing angle deposition and dip coating techniques. The heterojunction photoanode covered by a FeNiO_x_ cocatalyst (Ta_3_N_5_‐NRs/BaTaO_2_N/FeNiO_x_) generated a stable photocurrent density of ≈4.5 mA cm^−2^ at 1.23 V versus RHE under AM 1.5G simulated sunlight and 34%−35% IPCEs in the broad range of 380–540 nm were achieved. The BaTaO_2_N shell on Ta_3_N_5_ nanorods improved visible light harvesting, leading to the efficient generation and extraction of charge carriers and a stable evolution of stoichiometric O_2_ (36.2 µmol h^−1^ cm^−2^) and H_2_ (72.4 µmol h^−1^ cm^−2^) with Faradaic efficiencies near 96% (Figure 20d). Since Pt‐modified BaTaO_2_N–BaNa_0.25_Ta_0.75_O_3_ solid solution exhibited an enhanced photocatalytic activity for H_2_ evolution, Luo et al.^[^ ^187^ ^]^ further studied the Z‐scheme overall water splitting by involving the BaTaO_2_N–BaNa_0.25_Ta_0.75_O_3_ solid solution as the H_2_‐evolving photocatalyst, surface‐treated WO_3_ as the O_2_‐evolving photocatalyst, and IO_3_^−^/I^−^ as a redox mediator under visible light irradiation. The system prepared with the BaTaO_2_N–BaNa_0.25_Ta_0.75_O_3_ solid solution exhibited the highest photocatalytic activity with 14.46 µmol h^−1^ H_2_ and 6.68 µmol h^−1^ O_2_ evolution rates (Figure 19a). Castelli et al.^[^ ^197^ ^]^ computationally investigated the band gaps and optical properties of functional perovskites composed of the layers of two cubic perovskite semiconductors (BaSnO_3_ and BaTaO_2_N) and found that the different layers of perovskites can be used to design a direct band gap of functional perovskites between 2.3 and 1.2 eV. The stacking of BaSnO_3_ and BaTaO_2_N layers was described as a type‐II heterojunction, whereas the stacking of LaAlO_3_ and LaTiO_2_N layers was suggested for designing a type‐I heterojunction.

**Figure 21:**
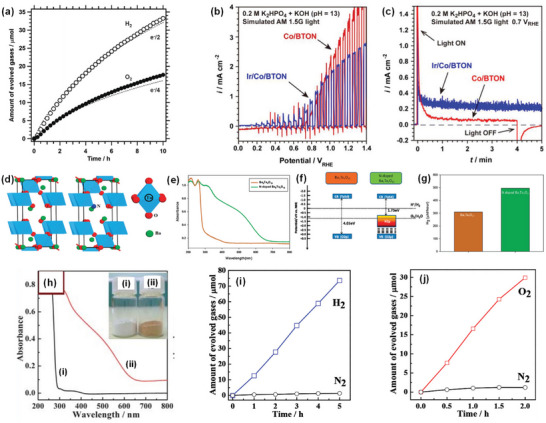
a) Unassisted PEC water splitting under visible light irradiation by a p/n PEC cell consisting of Pt/Al‐LTC_0.9_A_0.1_ (0.43 cm^2^) and Co/BaTaO_2_N (2.32 cm^2^) placed side by side. Reproduced with permission.^[^ ^198^ ^]^ Copyright 2015, The Royal Society of Chemistry. b) *I*–*E* curves and c) *I*–*t* curves recorded at 0.7 V versus RHE for Co/BaTaO_2_N and Ir/Co/BaTaO_2_N photoanodes in 0.2 m K_2_HPO_4_ aqueous solution (at pH 13 by KOH) under AM 1.5G simulated sunlight. Reproduced with permission.^[^ ^199^ ^]^ Copyright 2017, Wiley‐VCH Verlag GmbH & Co. KGaA. d) Structural models, e) UV‐Vis absorbance spectra, f) schematic representation of band structures, and g) H_2_ production rates of Ba_5_Ta_4_O_15_ and N‐doped Ba_5_Ta_4_O_15_ under simulated solar irradiation. Reproduced with permission.^[^ ^201^ ^]^ Copyright 2011, American Chemical Society. h) UV‐Vis diffuse reflectance spectra and digital photographs of Ba_5_Ta_4_O_15_ (i) and Ba_5_Ta_4_O_15‐x_N_x_ (ii) powders, i) reaction time course of H_2_ evolution of 0.3 wt% Pt/Ba_5_Ta_4_O_15‐x_N_x_, and j) reaction time course of O_2_ evolution of 1.0 wt% CoO_x_/Ba_5_Ta_4_O_15‐x_N_x_. Reproduced with permission.^[^ ^202^ ^]^ Copyright 2013, The Royal Society of Chemistry.

BaTaO_2_N was also involved as the O_2_‐evolving photocatalyst in the photoelectrochemical (PEC) overall water splitting. The *p*/*n* PEC cell, prepared by connecting cobalt species‐loaded BaTaO_2_N photoelectrodes and Pt‐loaded Al‐doped La_5_Ti_2_Cu_0.9_Ag_0.1_S_5_O_7_ solid solution, enabled to accomplish an unassisted PEC water splitting at a Faradaic efficiency of unity and an STH conversion efficiency of ≈0.1% under irradiation of up to 710 nm (**Figure** **21**a).^[^ ^198^ ^]^ Later, the surface modification of BaTaO_2_N with Ir and Co species led to the increased photocurrent density (0.26 mA cm^−2^) and an STH conversion efficiency (0.14%) at 0.7 V versus RHE, which were three times higher than those obtained for BaTaO_2_N modified only with Co species (Figure 21b,c).^[^ ^199^ ^]^ The PEC cell incorporating an Ir/Co‐BaTaO_2_N photoanode and Pt‐loaded TiO_2_/CdS‐modified Al‐doped La_5_Ti_2_Cu_0.9_Ag_0.1_S_5_O_7_ photocathode exhibited spontaneous overall water splitting with an STH conversion efficiency of 0.14% following a minute of AM 1.5G simulated sunlight. To achieve the targeted STH conversion efficiency, it is important to fabricate semi‐transparent front photoanodes for PEC tandem devices. Recently, a semi‐transparent three‐dimensional macroporous CaTaO_2_N photoanode was successfully fabricated on a GaN/Al_2_O_3_ substrate via a chemical route, which exhibited a high transmittance (>60%) in the wide solar spectrum, a photo‐response onset at −0.3 V versus RHE under simulated solar illumination, and a plateau photocurrent density of 0.21 mA cm^−2^ at 0.4 V versus RH, which is 50‐fold higher than that of particle‐based CaTaO_2_N/GaN/Al_2_O_3_, due to its efficient charge carrier separation and the reduced diffusion distance for minority carriers.^[^ ^200^ ^]^ Such strategies must be further optimized for the fabrication of semi‐transparent BaTaO_2_N photoanodes that can be used for the design of PEC tandem systems.

## 11 Other Compounds in the Ba‐Ta‐O‐N System

**Figure 22:**
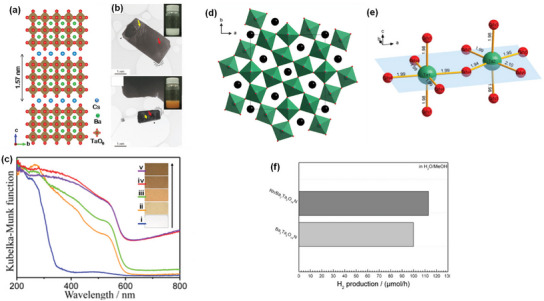
a) Crystal structure of CsBa_2_Ta_3_O_10_, b) TEM images of the fabricated nanosheets of CsBa_2_Ta_3_O_10_ and nitrided Ba_2_Ta_3_O_10_ crystals at 800 °C for 5 h, and c) UV−Vis diffuse reflectance spectra of CsBa_2_Ta_3_O_10_ (i) and nitrided CsBa_2_Ta_3_O_10_ crystals at 800 °C for 2 (ii), 5 (iii), 8 (iv), and 11 (v) h under an NH_3_ flow. Reproduced with permission.^[^ ^203^ ^]^ Copyright 2016, American Chemical Society. d) Unit cell of Ba_3_Ta_5_O_14_N and polyhedral representation of the crystal structure. Ta(O/N)_6_ octahedra – green; Ba – black balls. e) Ta(O/N)_6_ octahedra in Ba_3_Ta_5_O_14_N with the determined bond lengths (Å). f) Photocatalytic hydrogen evolution rates of Ba_3_Ta_5_O_14_N before and after reductive in situ photodeposition of 0.0125 wt% Rh. Reproduced with permission.^[^ ^204^ ^]^ Copyright 2016, Elsevier.

Various members of the quaternary Ba‐Ta‐O‐N system were also synthesized, and their solar water‐splitting performance was evaluated. For instance, Ba_2_TaO_3_N adopting the K_2_NiF_4_‐type structure with space group *I*4/*mmm* was synthesized by Clarke et al.^[^ ^112^ ^]^ using TaON and BaO at 1500 °C under 1 atm N_2_. It was found that O/N ordering dictated by the competition between the cations of different electronegativity for the anions was favored in the K_2_NiF_4_‐type structure, whereas O/N disorder was observed for perovskite structures. The (111)‐layered *B*‐site deficient hexagonal perovskite Ba_5_Ta_4_O_15_ was also doped with nitrogen.^[^ ^201^, ^202^ ^]^ The resulting Ba_5_Ta_4_O_15−_ *_x_* N*_x_* compounds showed a significantly enhanced visible light absorption due to the upward shift of the valence band maximum by N 2p states without affecting the conduction band minimum (Figure 21d–j). The unique layered structure provides intergallery spacings between the perovskite layers for the nitrogen dopant to diffuse easily, resulting in the uniform distribution of the nitrogen dopant. In the photocatalytic H_2_ (495 µmol h^−1^ in the presence of ethanol^[^ ^201^ ^]^ and 12 µmol h^−1^ in the presence of methanol^[^ ^202^ ^]^) and O_2_ (19.9 µmol h^−1[^ ^202^ ^]^) evolution reactions, the Ba_5_Ta_4_O_15−_ *_x_* N*_x_* compounds exhibited much higher photocatalytic activity with respect to pristine Ba_5_Ta_4_O_15_ (Figure 21h–j). New Dion−Jacobson phase three‐layer perovskite CsBa_2_Ta_3_O_10_ crystals were synthesized by a conventional solid‐state reaction, and the Ba_2_Ta_3_O_10‐x_N_x_ nanosheets with lateral sizes ranging from several hundred nanometers to a few micrometers and a thickness of about 2.3 nm were fabricated via the nitridation‐protonation‐intercalation‐exfoliation processes of CsBa_2_Ta_3_O_10_ (**Figure** **22**a,b).^[^ ^203^ ^]^ With increasing the ammonolysis time, the intensity of background absorption gradually became higher (Figure 22c). The two‐dimensional Ba_2_Ta_3_O_10‐x_N_x_ nanosheets can be applied to design novel systems for visible‐light‐driven water splitting. Ba_3_Ta_5_^V^O_14_N, which crystallizes isostructurally to Ba_3_Ta_4_^V^Ta^IV^O_15_, was synthesized by the ammonolysis of an amorphous ternary Ba‐Ta‐O phase under a mixture gas flow of NH_3_ and O_2_ at 850 °C for 24 h (Figure 22d,e).^[^ ^204^ ^]^ The synthesized Ba_3_Ta_5_^V^O_14_N exhibited a light‐yellow color, an optical bandgap energy of about 2.8 eV, and active surface sites for photocatalytic H_2_ generation with (115 µmol h^−1^ in the presence of Rh nanoparticles) and without (≈100 µmol h^−1^) any cocatalyst (Figure 22f). Using 2 m NaNO_2_ solution as an optical filter showed no significant activity in H_2_ generation because NaNO_2_ completely blocked the light with energies >3.05 eV, resulting in a low absorption window.

## 12 Effect of Cocatalyst on Water Splitting Performance of BaTaO_2_N

**Figure 23:**
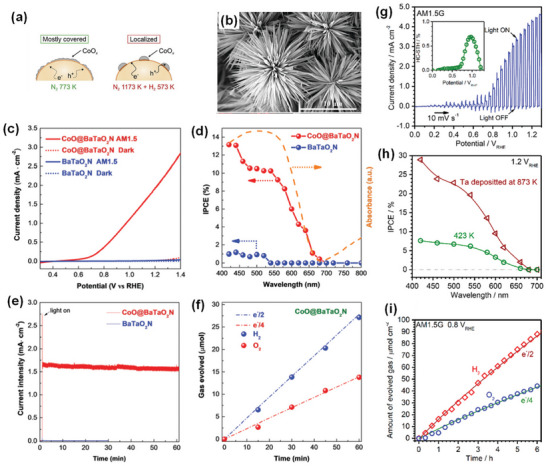
a) Comparison of CoO_x_/BaTaO_2_N photocatalysts annealed at 773 K under N_2_ flow and annealed at 1173 K under N_2_ flow and subsequently under H_2_ at 573 K. Reproduced with permission.^[^ ^208^ ^]^ Copyright 2020, Elsevier. b) FESEM image of CoO@BaTaO_2_N, c) LSV of BaTaO_2_N and CoO@BaTaO_2_N photoanodes, d) wavelength dependence of IPCE of BaTaO_2_N and CoO@BaTaO_2_N photoanodes with UV‐vis absorption spectrum of BaTaO_2_N, e) steady‐state photocurrents of BaTaO_2_N and CoO@BaTaO_2_N photoanodes, and f) temporal gas evolution from the PEC cell with CoO@BaTaO_2_N as the photoanode and Pt foil as the cathode. Reproduced with permission.^[^ ^209^ ^]^ Copyright 2021, The Royal Society of Chemistry. g) *I*−*E* curve of a Co/BaTaO_2_N/Ta/Ti photoelectrode under AM 1.5G simulated sunlight. A 0.2 m potassium phosphate aqueous solution adjusted to pH 13 by adding KOH was used as an electrolyte. h) Wavelength dependence of IPCE for Co/BaTaO_2_N/Ta/Ti electrodes and i) amounts of evolved hydrogen and oxygen of Co/BaTaO_2_N/Ta/Ti electrodes. A 0.2 m potassium phosphate aqueous solution (pH 13) and a CrO_x_‐coated Pt mesh were used as the electrolyte and counter electrode, respectively. Reproduced with permission.^[^ ^67^ ^]^ Copyright 2015, American Chemical Society.

**Figure 24:**
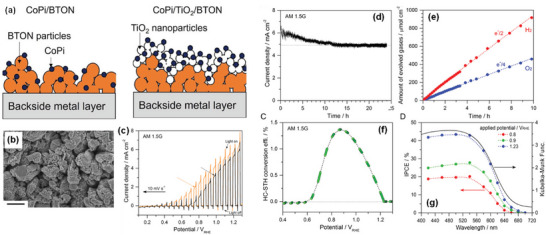
a) Schematic illustration of the assumed structures of CoPi/BaTaO_2_N and CoPi/TiO_2_/BaTaO_2_N. Reproduced with permission.^[^ ^210^ ^]^ Copyright 2021, AIP Publishing. b) SEM image of BaTaO_2_N powder, c) LSV data, d) chronoamperometry curve, e) reaction time courses of O_2_ and H_2_ generation, f) HC‐STH energy conversion efficiency, and g) IPCE values of Co(OH)_x_−FeO_y_/BaTaO_2_N photoanode at different applied potentials as functions of wavelength, with the UV−Vis DRS spectrum of BaTaO_2_N powder. Reproduced with permission.^[^ ^135^ ^]^ Copyright 2019, American Chemical Society.

**Figure 25:**
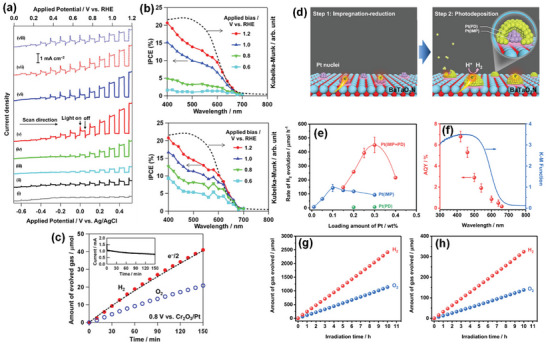
a) Current−potential curves in a phosphate buffer solution (pH 8) under visible light irradiation of i) BaTaO_2_N, ii) BaTaO_2_N(H_2_), iii) CoO_x_(3 wt%)/BaTaO_2_N, (iv) CoO_x_(3 wt%)/BaTaO_2_N(H_2_), v) post‐RhO_x_ (3 wt%)/CoO_x_ (0.5 wt%)/BaTaO_2_N(H_2_), vi) post‐IrO_x_(3 wt%)/CoO_x_(0.5 wt%)/BaTaO_2_N‐(H_2_), vii) post‐RhO_x_(3 wt%)/BaTaO_2_N(H_2_), and viii) post‐IrO_x_(3 wt%)/BaTaO_2_N(H_2_) electrodes. b) IPCE spectra of (*top*) CoO_x_(3 wt%)/BaTaO_2_N(H_2_) and (*bottom*) post‐RhO_x_(3wt%)/CoO_x_(0.5 wt%)/BaTaO_2_N(H_2_) electrodes with various applied potentials (phosphate buffer solution, pH 8), and absorption spectrum of BaTaO_2_N. c) Reaction time courses of H_2_ and O_2_ evolution in a two‐electrode system composed of post‐RhO_x_(3 wt%)/CoO_x_(0.5 wt%)/BaTaO_2_N(H_2_) electrode and Pt‐wire coated with Cr_2_O_3_ in a phosphate buffer solution (pH 8) under visible light irradiation. The inset indicates the change in the photocurrent. Reproduced with permission.^[^ ^65^ ^]^ Copyright 2013, American Chemical Society. d) Schematic representation of sequential Pt cocatalyst deposition on BaTaO_2_N, e) photocatalytic H_2_ evolution and f) apparent quantum yield of Pt‐modified BaTaO_2_N (RbCl), and (g,h) reaction time courses of H_2_ and O_2_ evolution during Z‐scheme water‐splitting reaction using Pt(0.1% IMP + 0.2% PD)/BaTaO_2_N. Reproduced with permission.^[^ ^211^ ^]^ Copyright 2021, Springer Nature AG & Co. KGaA.

Cocatalysts are often employed to activate and boost the photocatalytic and photoelectrochemical performance of semiconductor‐based photocatalysts because cocatalysts, their interfaces with semiconductors, dispersion, concentration, and size play an important role in improving the charge separation and transfer, and extending the lifetimes of photo‐excited states.^[^ ^205^, ^206^, ^207^ ^]^ Pt nanoparticles have been mostly used as the HER‐cocatalyst, whereas CoO_x_, Co(OH)_x_, Co_3_O_4_, IrO_2_, NiO, FeO_x_, RhO_x_, etc. have enhanced the photocatalytic and photoelectrochemical OER rate. Okamoto et al.^[^ ^208^ ^]^ successfully increased the photocatalytic O_2_ evolution rate three‐fold by annealing under an N_2_ flow after loading the Co species on the surfaces of BaTaO_2_N crystals in comparison to that prepared by annealing under an NH_3_ flow. The photocatalytic O_2_ evolution rate was further improved by a factor of two, yielding an apparent quantum efficiency of 0.55% at 420 nm, by subsequent annealing under an H_2_ atmosphere because the CoO_x_ particles were localized on the surface of BaTaO_2_N (**Figure** **23**a). An intimate contact formed between the CoO_x_ cocatalyst and BaTaO_2_N enabled an efficient transfer of photo‐excited electron‐hole pairs. Stable and efficient photoelectrochemical water splitting was achieved using a BaTaO_2_N photoanode decorated with CoO microflowers, which not only effectively collected the holes from BaTaO_2_N but also protected BaTaO_2_N against photocorrosion.^[^ ^209^ ^]^ The Faradaic efficiency of almost unity (99.2%) was recorded for OER, and the tips of the CoO microflowers were found to be the most active sites for OER (Figure 23b–f). Using a Co catalyst, a photoanode of particulate BaTaO_2_N fabricated by the particle transfer method using a Ta contact layer and a Ti conductor layer generated anodic photocurrent densities of 4.2 mA cm^−2^ at 1.2 V versus RHE under AM 1.5 G simulated sunlight and 25 mA cm^−2^ at 1.2 V versus RHE under visible light irradiation from 300 W Xe lamp (Figure 23g–i).^[^ ^67^ ^]^ The half‐cell solar‐to‐hydrogen conversion efficiency (HC‐STH) of the photoanode reached 0.7% at 1.0 V versus RHE, and the Faradaic efficiency for oxygen evolution was virtually 100% during the reaction for 6 h, indicating its robustness. A particulate BaTaO_2_N photoanode was modified with cobalt phosphate (CoPi)‐loaded TiO_2_ nanoparticles, resulting in an enhancement in the PEC‐OER performance.^[^ ^210^ ^]^ The TiO_2_ nanoparticles functioned as transparent and conductive support with a high surface area to immobilize CoPi nanoparticles on the photoanode surface (**Figure** **24**a). This led to improved reaction kinetics and increased electrochemically active surface area of the CoPi cocatalysts by a factor of 7.45. The addition of (Na)Rh/Cr_2_O_3_ and IrO_2_ as cocatalysts was essential to achieve a one‐step excitation overall water splitting of BaTaO_2_N:Mg under visible light.^[^ ^169^ ^]^ Without IrO_2_, the water oxidation reaction did not proceed on the bare BaTaO_2_N:Mg surface, and the net activity was reduced. In comparison to 1.5 wt% IrO_2_ loaded on BaTaO_2_N in the previous study,^[^ ^181^ ^]^ 0.3 wt% IrO_2_ in this study was found to be suitable with 6 wt% Rh and 6 wt% Cr to drive a one‐step excitation overall water splitting of BaTaO_2_N:Mg. Seo et al.^[^ ^135^ ^]^ achieved a photocurrent density of 6.5 mA cm^−2^ at 1.23 V versus RHE for BaTaO_2_N, which was activated by annealing in Ar, loaded with Co(OH)_x_‐FeO_y_ cocatalyst during solar water splitting, corresponding to a maximum STH conversion efficiency of 1.4% at 0.88 V versus RHE (Figure 24b–e). The Na–Pt cocatalyst was involved along with Cr_2_O_3_, which was employed to suppress reverse reactions, over BaTaO_2_N:Zr0.01.^[^ ^172^ ^]^ The Cr_2_O_3_/Na–Pt/BaTaO_2_N:Zr0.01 was almost inactive in aqueous solutions containing I^−^ or Fe^2+^ shuttle ions, while it exhibited a decent activity when [Fe(CN)_6_]^4−^ was employed as the electron donor. The Z‐scheme system based on Cr_2_O_3_/Na–Pt/BaTaO_2_N:Zr0.01 as the H_2_‐evoling photocatalyst, CoO*_x_*/Au/BiVO_4_ as the O_2_‐evolving photocatalyst, and [Fe(CN)_6_]^3−/4−^ as redox ions evolved H_2_ and O_2_ under visible light up to 520 nm, with the STH conversion efficiency of 0.022%. Compared with an individual loading, the combination of pre‐loading of CoO_x_ on the BaTaO_2_N particles and post‐loading of RhO_x_ was found to be highly efficient in improving both the photocurrent efficiency and stability under visible light irradiation and shifted the onset potential for water oxidation negatively (≈300 mV) (**Figure** **25**a–c).^[^ ^65^ ^]^ A stepwise loading of a Pt cocatalyst by impregnation–reduction and subsequent photo‐deposition remarkably enhanced the photocatalytic H_2_ evolution activity of single‐crystalline particulate BaTaO_2_N (Figure 25d–h).^[^ ^211^ ^]^ The photocatalytic H_2_ evolution rate of BaTaO_2_N was dependent on the amount of deposited Pt nanoparticles, among which the deposition of 0.5 wt% Pt cocatalyst exhibited the optimal activity.^[^ ^118^ ^]^ The sequential decoration method produced highly dispersed and uniformly sized Pt active sites firmly on BaTaO_2_N, enabling the rapid transfer of photo‐excited electrons across the interface and active H_2_ evolution reaction on the surface. As an outcome, Pt‐loaded BaTaO_2_N exhibited an apparent quantum yield of 6.8 ± 0.5% at 420 nm for photocatalytic H_2_ evolution from a sacrificial methanol aqueous solution, and an apparent quantum yield of 4.0% at 420 nm and an STH conversion efficiency of 0.24% in Z‐scheme water splitting. Recently, one‐step‐excitation overall water splitting using pristine BaTaO_2_N, which was synthesized by the direct ammonolysis of a mixture of amorphous Ta_2_O_5_ (Ta_2_O_5_·3H_2_O) nanoparticles and BaCO_3_, was achieved for the first time by modification with Rh (or Ru), Cr_2_O_3_, and IrO_2_ as cocatalysts.^[^ ^212^ ^]^ It was found that the photocatalytic activity of pristine BaTaO_2_N was influenced by the concentrations of the chosen cocatalysts, and the optimal amounts of Rh (or Ru), Cr_2_O_3_, and IrO_2_ were determined to be 4 wt% (2 wt%), 1 wt%, and 0.3 wt%, respectively. The apparent quantum yield and STH conversion efficiency of Rh/Cr_2_O_3_/IrO_2_‐loaded BaTaO_2_N reached 0.1% at 400 nm and 5 × 10^−4^%, respectively. Dual cocatalysts loaded by photodeposition can promote simultaneous oxidation and reduction reactions of photocatalysts. However, it is still challenging to photodeposit oxygen evolution cocatalysts because of weak driving forces for oxidation reaction. Recently, Kobayashi et al.^[^ ^213^ ^]^ successfully loaded the FeO_x_ cocatalyst onto a Mg‐doped BaTaO_2_N photocatalyst by an oxidative photodeposition technique, and BaTaO_2_N coloaded with Pt and FeO_x_ exhibited an apparent quantum yield of 1.2% at 420 nm during the oxygen evolution reaction. It is noteworthy that this photodeposition does not require a further heat treatment, which is advantageous for the design of novel cocatalyst‐loaded photocatalysts that are prone to thermal decomposition at elevated temperature.

## 13 Photocatalytic and Photoelectrochemical Performance of BaTaO_2_N

The photocatalytic and photoelectrochemical performance of BaTaO_2_N for the water‐splitting reaction is presented in the previous sections with respect to its synthesis methods, film fabrication, doping, solid solution, heterostructures, heterojunctions, and cocatalysts. This analysis also includes the effect of morphology, particle size, porosity, and surface chemistry. As a final objective, most of the studies seek to efficiently generate H_2_ from water splitting using BaTaO_2_N under simulated AM 1.5G solar illumination. It is emphasized that all these strategies are based on the requirement that the valence band potential of semiconductor must be more positive than that required for the oxidation of water, while the conduction band potential must be more negative than that required for the reduction of water. The generated H_2_ flux, apparent quantum yield (AQY), and rate constants are often used to compare the photocatalytic performance. Quantitative approaches toward the evaluation of the photocatalytic water splitting were previously recommended by Takanabe^[^ ^93^ ^]^ and Kisch and Bahnemann.^[^ ^214^ ^]^

In the realm of BaTaO_2_N‐based photocatalysis, the photocatalytic performance has been reported to be closely dependent on the synthesis method, indicating a number of general aspects that allow exploiting the water decomposition reaction using BaTaO_2_N‐based materials. Some of these general aspects include synthesis methods,^[^ ^124^, ^171^ ^]^ anion doping,^[^ ^160^ ^]^ and cation doping^[^ ^65^, ^133^, ^163^ ^]^ to improve overall photocatalytic performance, combination with other materials for solid‐state Z‐scheme photocatalytic water splitting,^[^ ^118^, ^172^, ^187^, ^192^, ^194^, ^211^ ^]^ and the use of co‐catalysts to promote OER (Pt,^[^ ^107^, ^131^, ^132^, ^141^, ^164^, ^183^, ^184^, ^194^ ^]^ Rh,^[^ ^169^, ^172^ ^]^ CrO_3_^[^ ^169^, ^172^, ^211^ ^]^) and HER (PtO_x_,^[^ ^118^, ^192^, ^193^, ^194^ ^]^ CoO_x_,^[^ ^117^, ^129^, ^137^, ^165^, ^168^, ^177^, ^185^, ^207^ ^]^ and IrO_x_^[^ ^115^, ^181^, ^211^ ^]^). **Table** **2** summarizes the photocatalytic performance of BaTaO_2_N‐based photocatalysts.

**Table 2:** Photocatalytic water splitting activity of BaTaO_2_N‐based photocatalysts.

		Photocatalytic water splitting half‐reaction		Overall water splitting reaction			
HEP (*cocatalyst*)	OEP (*cocatalyst*)	Reaction conditions	Activity	Reaction conditions	Activity	Reference	Year
BaTaO_2_N (*0.3 wt% Pt*)	WO_3_ (*0.5 wt% Pt*)	5 mM NaI aqueous solution under visible light (300 W Xe lamp), 100 mg of HEP	≈3 µmol H_2_ evolved in 7 hours	5 mM NaI aqueous solution under visible light (300 W Xe lamp), 100 mg of HEP and 100 mg OEP	AQY = 0.1% at 420–440 nm in overall water splitting	[107]	2008
BaTaO_2_N (*0.3 wt% Pt*)	WO_3_ (*0.5 wt% Pt*)	‐	‐	5 mM NaI aqueous solution under visible light (300 W Xe lamp), 100 mg of HEP and 100 mg OEP	AQY = 0.1% at 420–440 nm in two‐step overall water splitting	[195]	2009
BaZrO_3_–BaTaO_2_N (*0.3 wt% Pt*)	‐	4 mM NaI aqueous solution under visible light (300 W Xe lamp), 50 mg of HEP	>3 µmol H_2_ evolved in 4 hours	‐	‐	[183]	2011
BaZrO_3_–BaTaO_2_N (*0.3 wt% Pt*)	WO_3_ (*0.5 wt% Pt*)	‐	‐	1 mM NaI aqueous solution under visible light (300 W Xe lamp), 50 mg of HEP and 100 mg of OEP	AQY = 0.6% at 420–440 nm in overall water splitting	[183]	2011
‐	BaWO_x_N_y_‐BaTaO_2_N (*1.5 wt% IrO_2_*)	10 mM AgNO_3_ aqueous solution and 0.1 g of La_2_O_3_ for O_2_ evolution under visible light (300 W Xe lamp), 100 mg of OEP	>100 µmol O_2_ evolved in 10 hours	‐	‐	[181]	2013
BaZrO_3_–BaTaO_2_N (*0.3 wt% Pt*)	WO_3_ (*0.5 wt% Pt*) or rutile‐TiO_2_	‐	‐	1 mM NaI aqueous solution under simulated sunlight (AM 1.5G, 50 mg of HEP and 100 mg of OEP	STH energy conversion efficiencies of 0.0067% and 0.014% for Pt/BaZrO_3_‐BaTaO_2_N + Pt/WO_3_ and Pt/BaZrO_3_‐BaTaO_2_N + rutile‐TiO_2_, respectively, in solar‐driven Z‐scheme water splitting	[184]	2013
BaZrO_3_–BaTaO_2_N (*0.3 wt% Pt*)	BaZrO_3_–BaTaO_2_N (*1.5% IrO_2_*)	10 vol.% methanol solution for H_2_ evolution; 10 mM AgNO_3_ aqueous solution and 100 mg of La_2_O_3_ for O_2_ evolution; under visible light (300 W Xe lamp), 100 mg of HEP and 100 mg of OEP	AQYs = 0.06% and 0.03% at 420 nm in H_2_ and O_2_ evolution, respectively	‐	‐	[115]	2014
BaTaO_2_N (*0.3 wt% Pt*)	BaTaO_2_N (*2 wt% CoO_x_*)	10 vol.% methanol solution for H_2_ evolution and 10 mM AgNO_3_ aqueous solution and 0.1 g of La_2_O_3_ for O_2_ evolution under visible light (300 W Xe lamp), 100 mg of HEP and 100 mg of OEP	>16 µmol H_2_ and >20 µmol O_2_ evolved in 14 and 6 hours, respectively	‐	‐	[129]	2015
‐	BaTaO_2_N (*2 wt% CoO_x_*)	10 mM AgNO_3_ aqueous solution and 200 mg of La_2_O_3_ for O_2_ evolution; under visible light (300 W Xe lamp), 100 mg of OEP	AQY = 0.24% at 420 nm in O_2_ evolution; 331.1 µmol h^−1^ O_2_ evolution rate in the first 1 h	‐	‐	[177]	2016
BaTaO_2_N (*0.5 wt% Pt*)	WO_3_ (*0.45 wt% PtO_x_*)	20 vol.% methanol solution for H_2_ evolution under visible light (300 W Xe lamp), 100 mg of HEP	16.0 µmol h^−1^ H_2_ (≈60 µmol in total in 5 hours)	2 mM NaI aqueous solution under visible light (300 W Xe lamp), 50 mg of HEP and 100 mg of OEP	3.1 µmol h^−1^ H_2_ and 1.55 µmol h^−1^ O_2_ were evolved in Z‐scheme overall water splitting; AQY = 0.06% at 420 nm in overall water splitting	[118]	2017
Ta_3_N_5_/BaTaO_2_N (*0.5 wt% Pt*)	WO_3_ (*0.45 wt% PtO_x_*)	‐	‐	1 mM NaI aqueous solution under visible light (300 W Xe lamp), 50 mg of HEP and 50 mg of OEP	AQY = 0.1% at 420 nm in Z‐scheme overall water splitting	[192]	2017
BaTaO_2_N (*0.3 wt% Pt*)	BaTaO_2_N (*2 wt% CoO_x_*)	20 vol.% methanol solution for H_2_ evolution and 0.01 m AgNO_3_ aqueous solution and 0.1 g of La_2_O_3_ for O_2_ evolution under visible light (300 W Xe lamp), 100 mg of HEP and 100 mg of OEP	>0.25 µmol H_2_ and >2.0 µmol O_2_ evolved in 5 hours	‐	‐	[137]	2017
BaTaO_2_N (*0.3 wt% Pt*)	‐	20 vol.% methanol solution for H_2_ evolution under visible light (300 W Xe lamp), 100 mg of HEP	about 20 µmol H_2_ evolved in 5 hours	‐	‐	[141]	2017
‐	Ca‐doped BaTaO_2_N (*2 wt% CoO_z_*)	0.01 m AgNO_3_ aqueous solution and 200 mg of La_2_O_3_ for O_2_ evolution; under visible light (300 W Xe lamp), 100 mg of OEP	AQY = 2.1% at 420 ± 20 nm in O_2_ evolution	‐	‐	[162]	2018
‐	BaTaO_2_N (*2 wt% CoO_x_*)	10 mM AgNO_3_ aqueous solution and 200 mg of La_2_O_3_ for O_2_ evolution; under visible light (300 W Xe lamp), 100 mg of OEP	134.64 µmol h^−1^ (404 µmol in total) O_2_ evolved in 3 hours	‐	‐	[117]	2019
1D Ta_3_N_5_ nanorod/BaTaO_2_N nanoparticle (*0.3 wt% Pt*)	WO_3_ (*0.45 wt% PtO_x_*)	20 vol.% methanol solution for H_2_ evolution under visible light (300 W Xe lamp), 150 mg of HEP	27.3 µmol h^−1^ H_2_ evolved	2.0 × 10^−3^ m NaI aqueous solution for overall water splitting under visible light (300 W Xe lamp), 50 mg of HEP and 100 mg of OEP	4.8 µmol h^−1^ H_2_ and 2.4 µmol h^−1^ O_2_ evolved	[193]	2019
BaMg_1_ *_/_*_3_Ta_2_ *_/_*_3_O_3‐_ *_x_* N*_y_*/Ta_3_N_5_ (*0.5 wt% Pt*)	WO_3_ (*0.45 wt% PtO_x_*)	‐	‐	0.8 mM NaI aqueous solution for Z‐scheme overall water splitting under visible light (300 W Xe lamp), 50 mg of HEP and 100 mg of OEP	AQY = 0.1% at 420 nm in Z‐scheme overall water splitting	[194]	2019
BaTaO_2_N (*0.1 wt% Pt*)	‐	10 vol.% methanol solution for H_2_ evolution under visible light (300 W Xe lamp), 100 mg of HEP	BaTaO_2_N with {100} and {110} facets showed a 10‐fold increase in H_2_ evolution than BaTaO_2_N crystals with only {100} facets about 10 µmol H_2_ evolved in 6 hours (≈2 µmol h^−1^)	‐	‐	[131]	2019
‐	BaTaO_2_N (*2 wt% CoO_x_*)	10 mM AgNO_3_ aqueous solution and 100 mg of La_2_O_3_ for O_2_ evolution; under visible light (300 W Xe lamp), 100 mg of OEP	AQYs = 11.9% and 0.09% at 420 nm and 640 nm in O_2_ evolution, respectively; 699±13 µmol h^−1^ O_2_ evolution rate	‐	‐	[165]	2020
‐	Mg‐doped BaTaO_2_N (*2 wt% CoO_x_*)	0.05 m AgNO_3_ aqueous solution and 100 mg of La_2_O_3_ for O_2_ evolution; under visible light (300 W Xe lamp), 100 mg of OEP	AQY = 2.59% at 420 ± 20 nm in O_2_ evolution	‐	‐	[168]	2020
‐	BaTaO_2_N (0.5 *wt% CoO_x_*)	20 mM AgNO_3_ aqueous solution and 0.1 g of La_2_O_3_ for O_2_ evolution; under visible light (300 W Xe lamp), 100 mg of OEP	AQY = 0.55% at 420 nm in O_2_ evolution	‐	‐	[208]	2020
BaTaO_2_N (*0.1 wt% Pt*)	‐	10 vol.% methanol solution for H_2_ evolution under visible light (300 W Xe lamp), 100 mg of HEP	3.65 µmol h^−1^ H_2_ evolved	‐	‐	[132]	2020
BaTaO_2_N (*Na‐free 0.3 wt% Pt*)	WO_3_ (*H^+^–Cs^+^–0.5 wt% PtO_x_*)	‐	‐	2 mM NaI aqueous solution under visible light (300 W Xe lamp), 100 mg of HEP and 200 mg of OEP	stoichiometric amounts of H_2_ and O_2_ evolved, and a minor decrease in activity was observed after 12 hours	[164]	2021
BaTaO_2_N (*0.28 wt% Pt and 0.23 wt% Na*)	‐	15 vol.% methanol solution for H_2_ evolution under visible light (300 W Xe lamp), 100 mg of HEP	AQY = 1% at 420 nm in H_2_ evolution	‐	‐	[164]	2021
‐	LaTaON_2_–BaTaO_2_N (*2 wt% CoO_x_*)	0.01 m AgNO_3_ aqueous solution and 0.2 g of La_2_O_3_ for O_2_ evolution; under visible light (300 W Xe lamp), 100 mg of OEP	AQY = 3.91% at 420 ± 20 nm in O_2_ evolution	‐	‐	[185]	2021
BaTaO_2_N (*Pt(0.1%IMP + 0.2%PD)*)	‐	10 vol.% methanol solution for H_2_ evolution under visible light (300 W Xe lamp), 100 mg of HEP	AQYs = 6.8 ± 0.5% at 420 nm (±25 nm), 2.9 ± 0.4% at 500 nm (±25 nm), and 0.8 ± 0.3% at 600 nm (±25 nm) in H_2_ evolution >1000 µmol H_2_ evolved in 5 hours	‐	‐	[211]	2021
BaTaO_2_N (*Pt(0.1%IMP + 0.2%PD)*)	WO_3_ (*0.5 wt% Pt*)	‐	‐	3 mM NaI aqueous solution under AM 1.5G simulated sunlight, 100 mg of HEP and 150 mg of OEP	AQY = 4.0% at 420 nm and STH energy conversion efficiency of 0.24% in Z‐scheme water splitting	[211]	2021
5% Al‐, Mg‐ or Zr‐doped BaTaO_2_N (*0.5 wt% Pt*)	5% Al‐, Mg‐ or Zr‐doped BaTaO_2_N (*2 wt% CoO_x_*)	10 mM AgNO_3_ aqueous solution and 200 mg of La_2_O_3_ for O_2_ evolution; under visible light (300 W Xe lamp), 100 mg of HEP and 100 mg of OER	503.6 µmol O_2_ evolved on Mg‐doped BaTaO_2_N and 117.4 µmol H_2_ evolved on Al‐doped BaTaO_2_N, while both 446.8 µmol O_2_ and 80.4 µmol H_2_ evolved on Zr‐doped BaTaO_2_N in 5 hours	‐	‐	[133]	2022
Mg‐doped BaTaO_2_N (*Cr_2_O_3_ (6 wt % Cr)/Na (0.7 wt %) – Rh (6 wt %)*)	Mg‐doped BaTaO_2_N (*0.3 wt% IrO_2_*)	‐	‐	Ultrapure water under 300 W xenon lamp (>420 nm) or AM 1.5G simulated sunlight, 50 mg of HEP‐OER	AQY = 0.08% at 420 nm in overall water splitting and STH energy conversion efficiency of 4 × 10^−4^%	[169]	2022
1% Zr‐doped BaTaO_2_N (*Cr_2_O_3_ (0.9 wt% Cr)/0.23 wt% Na‐0.3 wt% Pt*)	‐	6 mM K_4_[Fe(CN)_6_] aqueous solution for H_2_ evolution under visible light (300 W Xe lamp), 100 mg of HEP	AQY = 0.4% at 420 nm in H_2_ evolution	‐	‐	[172]	2023
1% Zr‐doped BaTaO_2_N (*0.9 wt% Cr_2_O_3_/0.23 wt% Na 0.3 wt% P*t)	BiVO_4_ (*0.5 wt% CoO_x_/0.2 wt% Au*)	‐	‐	25 mM sodium phosphate buffer solution (pH 6) containing 6 mM K_4_[Fe(CN)_6_] (redox mediator) under AM 1.5G simulated sunlight, 70 mg of HEP and 100 mg of OEP	AQYs = 1.5% and 0.2% at 420 nm and 520 nm, respectively, and STH energy conversion efficiency of 0.022% in Z‐scheme overall water splitting	[172]	2023
BaTaO_2_N (*2 wt% Rh, 1 wt% Cr_2_O_3_ and 0.3 wt% IrO_2_*)		‐	‐	Ultrapure water under visible light (300 W Xe lamp) and AM 1.5G simulated sunlight, 200 mg of HEP‐OER	AQY = 0.1% at 420 nm and STH energy conversion efficiency of 5 × 10^−4^% in one‐step‐excitation overall water splitting	[212]	2023
‐	BaTaO_2_N:Mg (0.1 *wt% FeO_x_ and* 0.1 wt% Pt)	10 mM AgNO_3_ aqueous solution and 100 mg of La_2_O_3_ for O_2_ evolution; under visible light (300 W Xe lamp), 200 mg of OEP	AQY = 1.2% at 420 nm in O_2_ evolution	‐	‐	[213]	2023

The photoelectrochemical studies provide important information to the understanding of the performance of the photocatalysts in the water‐splitting reaction. Since BaTaO_2_N is an *n*‐type semiconductor, thin films of BaTaO_2_N can be fabricated on conductive substrates and used as photoanodes for photoelectrochemical characterization. Under simulated AM 1.5G sunlight, a photocurrent density of ≈18 mA cm^−2[^ ^67^ ^]^ and a solar‐to‐hydrogen (STH) conversion efficiency of about 24%^[^ ^10^ ^]^ can be theoretically achieved for BaTaO_2_N. However, the experimentally obtained results are much lower than the predicted values due to unsuitable surface states, photocorrosion, and various types of defects formed during the synthesis process. Bandgap engineering through mono and dual substitution,^[^ ^133^, ^174^ ^]^ solid solutions,^[^ ^177^, ^185^ ^]^ defect density control,^[^ ^124^, ^129^, ^150^, ^169^ ^]^ thin film fabrication,^[^ ^117^, ^151^ ^]^ and the optimization of exposed surfaces, particle morphology, porosity, and size^[^ ^131^, ^134^ ^]^ have been explored to enhance the photoelectrochemical performance of BaTaO_2_N.

**Figure 26:**
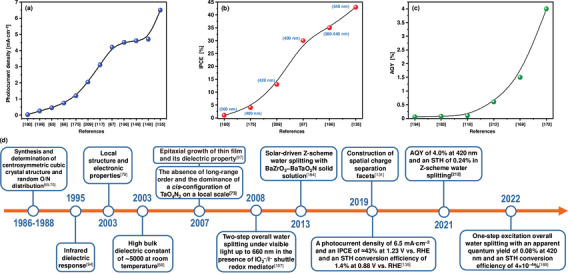
Photocurrent densities (a), incident photon‐to‐current conversion efficiencies (b), and apparent quantum yields (c) achieved with BaTaO_2_N. (d) Timeline of significant scientific advances on BaTaO_2_N.

Remarkable advances have been made, progressively increasing the photocurrent densities for BaTaO_2_N to ≈0.03 mA cm^−2^,^[^ ^180^ ^]^ >1.2 mA cm^−2^,^[^ ^175^ ^]^ 2.05 mA cm^−2^,^[^ ^209^ ^]^ 4.2 mA cm^−2^,^[^ ^67^ ^]^ ≈4.5 mA cm^−2^,^[^ ^196^ ^]^ and 6.5 mA cm^−2[^ ^135^ ^]^ at 1.2 V versus RHE (**Table** **3**, **Figure** **26**a). Similarly, the incident photon to current conversion efficiency (IPCE) has been steadily improved with values of 1% at 500 nm,^[^ ^180^ ^]^ >4% at 400 nm,^[^ ^175^ ^]^ 13% at 420 nm,^[^ ^209^ ^]^ ≈30% at 400 nm,^[^ ^67^ ^]^ 34–35% at 380–540 nm^[^ ^196^ ^]^ and ≈43% at 540 nm^[^ ^135^ ^]^ at 1.2 V versus RHE (Table 3, Figure 26b). The apparent quantum yield (AQY) for overall water splitting over BaTaO_2_N sequentially reached 0.06%,^[^ ^118^ ^]^ 0.08%,^[^ ^169^ ^]^ 0.1%,^[^ ^194^ ^]^ 0.6%,^[^ ^183^ ^]^ 1.5%,^[^ ^172^ ^]^ and 4.0%^[^ ^211^ ^]^ at 420 nm (Table 2, Figure 26c). However, the half‐cell solar‐to‐hydrogen conversion efficiency (HC‐STH) of BaTaO_2_N has only reached 1.4% at 0.88 V versus RHE.^[^ ^135^ ^]^ This indicates the existence of more room for further improvements in order to meet the current requirements for practical application.

**Table 3:** Photoelectrochemical performance of BaTaO_2_N‐based photoanodes.

Electrode (*fabrication method*)	Reaction conditions	Performance				Reference	Year
		Photocurrent density	IPCE/ABPE	H_2_ and O_2_ evolution	STH/HC‐STH		
IrO_2_/TiO_2_/BaZrO_3_‐BaTaO_2_N (*electrophoretic deposition*)	*Reference electrode*: Ag/AgCl; *Electrolyte*: Na_2_SO_4_ solution (pH 5.9); *Light source*: 300 W Xe lamp	≈0.03 mA cm^−2^ at 1.23 V vs RHE	1.0% IPCE at 1.2 V vs RHE at 500 nm	0.5 µmol h^−1^ H_2_ and 0.25 µmol h^−1^ O_2_	0.0011%	[180]	2012
RhO_x_/CoO_x_/BaTaO_2_N/Ti (*electrophoretic deposition and post‐necking method*)	*Reference electrode*: Ag/AgCl; *Electrolyte*: 0.1 m Na_2_HPO_4_ and 0.1 m NaH_2_PO_4_ (pH 8); *Light source*: 300 W Xe lamp and AM 1.5G simulated sunlight	0.46 mA cm^−2^ at 1.2 V vs RHE	10% IPCE at 1.2 V vs RHE at 600 nm	41.0 µmol H_2_ and 20.9 µmol O_2_ were evolved in a two‐electrode system within 150 min with an applied bias of 0.8 V vs counter electrode	‐	[65]	2013
Co/BaTaO_2_N/Ta/Ti (*particle transfer method*)	*Reference electrode*: Ag/AgCl; *Electrolyte*: 0.2 m potassium phosphate (pH 13 by KOH); *Light source*: 300 W Xe lamp and AM 1.5G simulated sunlight	4.2 mA cm^−2^ at 1.2 V vs RHE under AM 1.5 G simulated sunlight and 25 mA cm^−2^ at 1.2 V vs RHE under light irradiation from 300 W Xe lamp	≈30% IPCE at 1.2 V vs RHE at 400 nm	O_2_ evolved within 6 hours with a Faradaic efficiency of virtually 100% without significant deactivation	0.7% at 1.0 V vs RHE	[67]	2015
BaTaO_2_N:Mo‐5%/Ti (*electrophoretic deposition*)	*Reference electrode*: Ag/AgCl; *Electrolyte*: 0.5 m Na_2_SO_4_ (pH 6) and a phosphate buffer solution (pH 8); *Light source*: 300 W Xe lamp	>1.2 mA cm^−2^ at 1.23 V vs RHE	>4% IPCE at 1.2 V vs RHE at 400 nm	39.0 µmol H_2_ and 17.4 µmol O_2_ were evolved in two‐electrode system with a Faradaic efficiency of about 93% in both reactions	‐	[175]	2015
A p/n PEC cell, prepared by connecting cobalt species‐loaded BaTaO_2_N (2.32 cm^2^) and Pt‐loaded Al‐doped La_5_Ti_2_Cu_0.9_Ag_0.1_S_5_O_7_ solid solution (0.43 cm^2^) (*particle transfer method*)	*Reference electrode*: Ag/AgCl; *Electrolyte*: 0.1‐0.5 m Na_2_SO_4_ at pH 10–11 (NaOH adjusted); *Light source*: 300 W Xe lamp and AM 1.5G simulated sunlight	‐	‐	Unassisted stoichiometric O_2_ and H_2_ evolved with a Faradaic efficiency of unity	0.1%	[198]	2015
CoPi/BaTaO_2_N (*Hydrothermal synthesis of oxide precursor and ammonolysis*)	*Reference electrode*: Ag/AgCl; *Electrolyte*: 0.5 m potassium phosphate solution (pH 13); *Light source*: 300 W Xe lamp and AM 1.5G simulated sunlight	≈0.75 mA cm^−2^ at 1.23 V vs RHE	8% IPCE at 1.2 V vs RHE at 400 nm	56.0 µmol H_2_ and 27 µmol O_2_ were evolved in two‐electrode system within 5 hours. O_2_ evolved for 5 h with a Faradaic efficiency of >90% without significant deactivation	‐	[66]	2016
BaTaO_2_N (flux grown) (*particle transfer method*)	*Reference electrode*: Ag/AgCl; *Electrolyte*: 0.2 m K_2_HPO_4_ solution at pH 13 (KOH adjusted); *Light source*: 300 W Xe lamp and AM 1.5G simulated sunlight	>0.85 mA cm^−2^ at 1.23 V vs RHE	5% IPCE at 1.0 V vs RHE at 420 nm	‐	‐	[177]	2016
Ir/Co‐BaTaO_2_N (*particle transfer*)	*Reference electrode*: Ag/AgCl; *Electrolyte*: 0.1 m Na_2_HPO_4_ at pH 13 (NaOH adjusted); *Light source*: 300 W Xe lamp	0.26 mA cm^−2^ at 0.7 V vs RHE	‐	‐	0.14%	[199]	2017
A PEC cell, prepared by incorporating an Ir/Co‐BaTaO_2_N (2.15 cm^2^) and Pt‐loaded TiO_2_/CdS‐modified Al‐doped La_5_Ti_2_Cu_0.9_Ag_0.1_S_5_O_7_ (1.26 cm^2^) (*particle transfer*)	*Reference electrode*: Ag/AgCl; *Electrolyte*: aqueous phosphate solution at pH 13; *Light source*: 300 W Xe lamp and AM 1.5G simulated sunlight	0.11 mA cm^−2^ after 1 min	‐	1.1 µmol cm^−2^ h^−1^ H_2_ and 0.55 µmol cm^−2^ h^−1^ O_2_ evolved with a Faradaic efficiency of about 100%	0.14%	[199]	2017
CoO_z_/BaCa_0.10_Ta_0.90_O_2.27_N_0.73_ (*electrophoretic deposition*)	*Reference electrode*: Ag/AgCl; *Electrolyte*: 0.1 m K_3_PO_4_/K_2_HPO_4_ aqueous solution at pH 7.95; *Light source*: 300 W Xe lamp	≈0.88 mA cm^−2^ at 1.23 V vs RHE	‐	‐	‐	[162]	2018
Co(OH)_x_‐FeO_y_/BaTaO_2_N (*particle transfer method*)	*Reference electrode*: Ag/AgCl; *Electrolyte*: 0.5 m potassium borate (pH 13); *Light source*: 300 W Xe lamp and AM 1.5G simulated sunlight	6.5 mA cm^−2^ at 1.23 V vs RHE	≈43, 20, and 20% IPCE at 540 nm at 1.23, 0.9, and 0.8 V vs RHE, respectively	H_2_ and O_2_ at the stoichiometric ratio of 2:1 were evolved with a Faradaic efficiency of nearly 100%. H_2_ at a rate of 93 µmol h^−1^ cm^−2^	1.4% at 0.88 V vs RHE	[135]	2019
CoO_x_‐BaTaO_2_N (*particle transfer*)	*Reference electrode*: Ag/AgCl; *Electrolyte*: 0.2 m K_2_HPO_4_ at pH 13 (KOH adjusted); *Light source*: 300 W Xe lamp and AM 1.5G simulated sunlight	3.11 mA cm^−2^ at 1.2 V vs RHE	‐	‐	‐	[117]	2019
CoO_x_‐BaTaO_2_N/Ta_2_N/Ta (*RF sputtering and ammonolysis*)	*Reference electrode*: Hg/HgO; *Electrolyte*: 0.1 m potassium phosphate electrolyte (pH 13); *Light source*: 300 W Xe lamp and AM 1.5G simulated sunlight	4.6 mA cm^−2^ at 1.23 V vs RHE	9% IPCE at 1.2 V vs RHE at 600 nm	Both H_2_ and O_2_ with a close stoichiometric ratio were produced with a Faradaic efficiency of almost 100%	‐	[149]	2020
Ta_3_N_5_‐NRs/BaTaO_2_N/FeNiO_x_ (*glancing angle deposition and dip‐coating*)	*Reference electrode*: Ag/AgCl; *Electrolyte*: 0.5 m K_2_HPO_4_ at pH 13 (KOH adjusted); *Light source*: 300 W Xe lamp and AM 1.5G simulated sunlight	≈4.5 mA cm^−2^ at 1.23 V vs RHE	34‐35% IPCE at 1.23 V vs RHE at 380–540 nm	Stoichiometric O_2_ (36.2 µmol h^−1^ cm^−2^) and H_2_ (72.4 µmol h^−1^ cm^−2^) evolved with Faradaic efficiencies of about 96%	0.45% at 1.01 V vs RHE	[196]	2020
CoO_x_/BaTa_0.95_Mg_0.05_O_2+x_N_1‐y_ (*electrophoretic deposition*)	*Reference electrode*: Ag/AgCl; *Electrolyte*: 0.1 m K_3_PO_4_/K_2_HPO_4_ aqueous solution at pH 12.66; *Light source*: 300 W Xe lamp and AM 1.5G simulated sunlight	≈1.0 mA cm^−2^ at 1.0 V vs RHE	≈3% IPCE at 1.23 V vs RHE at 420 nm	‐	‐	[168]	2020
CoO/BaTaO_2_N (*electrophoretic deposition*)	*Reference electrode*: Ag/AgCl; *Electrolyte*: 0.1 m KOH aqueous solution at pH 13; *Light source*: 300 W Xe lamp and AM 1.5G simulated sunlight	2.05 mA cm^−2^ at 1.23 V vs RHE	13% IPCE at 1.23 V vs RHE at 420 nm	Stoichiometric O_2_ and H_2_ evolved, and O_2_ evolved with a Faraday efficiency of 99.2%	‐	[209]	2021
CoPi/TiO_2_/BaTaO_2_N/Ti (*particle transfer method*)	*Reference electrode*: Ag/AgCl; *Electrolyte*: 1.0 m K_2_HPO_4_ at pH 13 (KOH adjusted); *Light source*: 300 W Xe lamp and AM 1.5G simulated sunlight	1.26 mA cm^−2^ at 1.23 V vs RHE	‐	‐	‐	[210]	2021
BaTaO_2_N nanoparticles (*dual‐source electron beam deposition*)	*Reference electrode*: Hg/HgO; *Electrolyte*: 1 m KOH (pH 13.6); *Light source*: 300 W Xe lamp and AM 1.5G simulated sunlight	4.7 mA cm^−2^ at 1.23 V vs RHE	1.18% ABPE at 1.23 V vs RHE and >30% at 1.23 V vs RHE at 475 nm	O_2_ evolved with a Faradaic efficiency of >95%	‐	[148]	2022

The photoelectrochemical studies have enabled the resolution of trends in visible‐light‐assisted water oxidation based on BaTaO_2_N photoanodes, revealing an inverse relationship between recombination and charge transfer phenomena.^[^ ^124^ ^]^ At low overpotentials, recombination governs the photo‐oxidation of water, resulting in relatively positive onset potentials at the studied interfaces.^[^ ^65^, ^66^, ^67^, ^124^ ^]^ The deactivation process under open‐circuit conditions highlights differences in the electronic properties of the modified BaTaO_2_N photocatalyst, leading to longer lifetimes of charge carriers for photo‐electrodes. By utilizing co‐catalysts and dopants, the photocurrent collection increases even at low overpotentials. Studies on current transients under cycles of light and dark revealed interesting behavior in BaTaO_2_N‐based materials, particularly regarding the balance between electron transfer and recombination. Generally, the current signal is higher during light exposure than in the dark, showing a transient behavior characterized by a current spike at the beginning of illumination. When the light is turned off, the photocurrent drops, exhibiting a negative overshoot. All these features are commonly observed at the semiconductor‐electrolyte interfaces, where recombination plays a significant role.^[^ ^215^ ^]^ Similar phenomena have been reported for various photoanodes, including TiN‐modified *Imma*‐LaTiO_2_N^[^ ^216^ ^]^ and BaTaO_2_N/carbonaceous materials.^[^ ^217^ ^]^ As a consequence of the photoelectrochemical behavior described, the carrier deactivation processes (recombination) limit the PEC response and can be improved by applying state‐of‐the‐art approaches.

## 14 Conclusions and Perspectives

In this review, the basic principles of photoelectrochemical water splitting and the merits and demerits of oxide‐based photocatalysts researched for photoelectrochemical oxygen evolution reactions were introduced. As one of the most intensively explored representatives of the transition metal (oxy)nitrides family, perovskite BaTaO_2_N has emerged as a promising photocatalyst for solar water splitting due to its capability to absorb visible light up to 660 nm, suitable band‐edge potentials for overall water splitting in the absence of an external bias, and theoretical solar‐to‐hydrogen energy conversion efficiency of ≈24% under AM 1.5G simulated sunlight. Over the past three decades, the crystal structure, anion ordering, electronic structure, dielectric, ferroelectric, and piezoelectric properties of BaTaO_2_N were investigated. The apparent quantum yield, photocurrent density, incident photon‐to‐current conversion efficiency, and solar‐to‐hydrogen conversion efficiency of BaTaO_2_N have been progressively improved. However, its solar‐to‐hydrogen conversion efficiency lags behind the benchmark efficiency required for practical application. This review presented various strategies applied for achieving enhanced solar water‐splitting efficiency. Namely, high‐ and low‐temperature synthesis techniques, advanced synthesis approaches, film fabrication, surface and bulk defect density controlling, crystal facet engineering, and particle morphology, size, and porosity tailoring were overviewed. Also, the impacts of cation doping (mono‐, di‐, tri‐, tetra‐, penta‐, and hexavalent cations), creating the solid solutions, forming the heterostructures and heterojunctions, designing the photoelectrochemical cells, and loading suitable cocatalysts were discussed. Other members of the Ba‐Ta‐O‐N system were highlighted for solar water splitting.

Despite significant progress made in the past decades (Figure 26), several challenges still remain in the realm of BaTaO_2_N for solar water splitting. These challenges encompass the precise control of various factors, such as homogeneity (e.g., oxygen‐nitrogen ratio), crystallinity, particle morphology/size, and defect density. Furthermore, there is a pressing need to identify a suitable deposition technique for BaTaO_2_N particles, which can preserve their physicochemical and optoelectronic properties. The ability to maintain a long‐term water‐splitting efficiency relies on a comprehensive understanding of surface properties and stability during photocatalytic and photoelectrochemical reactions. Scaling up the photoelectrochemical cells and photocatalytic reactors beyond the laboratory scale is yet another facet of the challenge.

Similar to other transition metal (oxy)nitrides, perovskite BaTaO_2_N is known to exhibit defects that have a substantial impact on its properties and water‐splitting performance. Hence, it is imperative to gain a deep understanding of the defect chemistry, including anion and cation vacancies, within BaTaO_2_N. This is crucial for the development of high‐efficiency BaTaO_2_N for future solar water‐splitting applications.

Addressing these challenges with a rigorous approach involves understanding the intricate structure‐property relationships and unraveling the nature of the photocatalyst‐cocatalyst interface at the atomic level. Furthermore, the continued exploration of the underlying kinetics and mechanisms, using operando/in‐situ characterization along with theoretical studies, may pave the way for further development of BaTaO_2_N as a promising material for solar water splitting. Also, in‐depth photoelectrochemical studies can provide valuable information on the interplay between electron transfer dynamics and recombination phenomena. Machine learning and advanced synthesis techniques have enormous potential for the discovery of new photocatalytic materials within the Ba‐Ta‐O‐N system. The optimization of photocatalyst‐electrocatalyst systems is also of utmost importance. Ongoing scientific efforts to integrate transition metal borides, carbides, nitrides, and chalcogenides in water oxidation as electrocatalysts/cocatalysts are very promising.

To achieve high solar‐to‐hydrogen (STH) conversion efficiency, the challenges related to the fabrication of semi‐transparent photoanodes need to be addressed for the development of tandem systems. To overcome these limitations and scale up BaTaO_2_N‐based photoelectrochemical systems, it is crucial to directly observe Fermi level pinning or unpinning, mitigate photocorrosion and recombination processes, and minimize energy losses during PEC reactor design, construction, and operation.

## Conflict of Interest

The authors declare no conflict of interest.
